# A Mechano-Feedback Loop Orchestrated by SUN1/2 Governs Cellular Mechanoadaptation via Lamina-Associated Domain Remodeling

**DOI:** 10.34133/research.1259

**Published:** 2026-05-14

**Authors:** Yafan Xie, Zhaoyan Zuo, Chenfei Lu, Yanjing Zhao, Liping Guo, Wei Xu, Fuhai Liu, Robert Guidoin, Haoyue Zhang, Juhui Qiu, Guixue Wang, Qin Peng

**Affiliations:** ^1^ Key Laboratory for Biorheological Science and Technology of Ministry of Education, State and Local Joint Engineering Laboratory for Vascular Implants, Bioengineering College, Chongqing University, Chongqing 400030, China.; ^2^ Institute of Systems and Physical Biology, Shenzhen Bay Laboratory, Shenzhen 518132, China.; ^3^ School of Life Science and Technology, Harbin Institute of Technology, Harbin 150001, China.; ^4^ College of Life Sciences, University of Chinese Academy of Sciences, Beijing 101408, China.; ^5^ Institute of Molecular Physiology, Shenzhen Bay Laboratory, Shenzhen 518132, China.; ^6^ Faculty of Medicine, Université Laval and CHU de Québec Research Centre, Quebec G1V 0A6, Canada.; ^7^ Institute of Panvascular Biology, JinFeng Laboratory, Chongqing 401329, China.

## Abstract

SUN1/2, core components of the linker of nucleoskeleton and cytoskeleton complex, transmit extracellular mechanical forces to nuclear lamina and chromatin. However, their role in regulating peripheral chromatin in mechanosensing and mechanoadaptation remains unclear. Using CRISPR/Cas9-mediated knockout of *Sun1* or *Sun2* in myoblasts, we identified a SUN1/2-dependent mechano-feedback loop. SUN1/2 depletion down-regulates genes for cell adhesion (e.g., integrin alpha-4) and for mechanotransduction (e.g., cell division cycle 42 and Ras homolog family member A). The primary mechanism involves redistribution of heterochromatin from nuclear periphery to the nucleoplasm and remodeling of lamina-associated domains (LADs), as an adaptive response to the loss of SUN proteins. Furthermore, lamin A/C acts as a key downstream effector, consistently modulating adhesion-related gene expression through the remodeling of LADs. Functionally, knockout of either *Sun1/2* or *Lmna* aggravates differentiation defects in C2C12 myoblasts and abolishes adaptive responses to mechanical cues. This study provides proof of concept that nuclear mechanotransduction proteins can modulate cellular mechanoadaptation via a mechano-feedback loop, which coordinates LAD reorganization with the expression of upstream mechanotransduction genes.

## Introduction

Cells are constantly exposed to dynamic mechanical cues in their microenvironment—such as shear stress, compression, extracellular matrix stiffness, and physical confinement—and they actively sense and adapt to these stimuli to maintain biochemical and biomechanical homeostasis [1–4]. This process, known as mechanoadaptation, is mediated by a coordinated mechanotransduction machinery: it begins with upstream force sensing through cell adhesion molecules like integrins, continues through midstream cytoskeletal remodeling and actomyosin-mediated force generation, and culminates in downstream nuclear deformation and alterations in chromatin organization. These mechanical signals are ultimately translated into changes in gene expression and cell fate decisions [5–9]. Our prior research demonstrates that stress granules respond to shear stress and modulate integrin and inflammation gene expression [10,11]. Recent advances have elucidated mechanoadaptive networks involving actin, microtubule, and calcium-mediated signaling that shape nuclear stability and transcriptional responses in mechanically challenged cells [12–15]. The nucleus functions not simply as a passive mechanosensing entity but as a central processing hub—integrating extracellular forces into chromatin reorganization and transcriptional control [16–18]. This process, often termed mechanical feedback, involves mechanosensitive transcription factors that regulate gene expression to help cells adapt to their mechanical microenvironment [2,19,20]. However, how nuclear mechanics directly calibrates gene expression through mechanical feedback remains elusive.

A bidirectional mechanical bridge between extracellular forces and the nucleus is mediated by the linker of nucleoskeleton and cytoskeleton (LINC) complex [21]—comprising SUN (Sad1/UNC-84)-domain proteins in the inner nuclear membrane and KASH-domain proteins (e.g., Nesprins) in the outer membrane [22]. This complex mechanically couples the cytoskeleton to the nuclear lamina [23,24], enabling force transmission and mechanotransduction. Importantly, genetic modulation of SUN1/2 influences nuclear biophysical properties, including size, stiffness, and polarization, underscoring their functional importance in mechanosensitive cell behaviors [25]. For example, SUN2 suppression has been shown to soften the nucleus and delay cellular senescence induced by mechanical stress [25]. Additionally, SUN1 contributes to the regulation of cytoskeletal force generation and promotes focal adhesion (FA) maturation [26]. Nevertheless, how SUN1/2-driven mechanotransduction regulates gene expression for cellular adaptation remains unclear.

Gene expression under mechanical stimulation is highly dependent on chromatin architecture and epigenetic modifications [27–29]. A key feature of genome organization is the enrichment of transcriptionally repressive heterochromatin at the nuclear periphery, where it associates with nuclear lamina through regions known as lamina-associated domains (LADs), and genes located in LADs are typically silenced, contributing to cell-type-specific transcriptional programs [30]. The nuclear scaffold protein lamin A/C plays a central role in modulating chromatin structure and stabilizing lamina–chromatin interactions [31,32]. Besides, remodeling cytoskeleton proteins such as F-actin can also alter lamina–chromatin engagement and promote gene expression [33]. Given these findings, a critical question emerges: how do SUN1/2 contribute to genome organization—particularly in regulating the dynamics of LAD in response to mechanical cues? We propose mechanical feedback that in adherent cells, the adhesion machinery, cytoskeleton, and nucleus form a mechanically balanced state. The loss of SUN1 or SUN2 disrupts force transmission into the nucleus, and this deficit feeds back to the system, leading to reduced expression of adhesion and cytoskeletal proteins. This nuclear mechanical feedback may serve as a rebalancing mechanism, representing a form of cellular adaptation.

In this study, we thus investigated the role of SUN1 and SUN2 in nuclear mechanotransduction and gene expression through an integrated multi-omics approach. Our results demonstrate that SUN1/2 depletion induces nuclear enlargement and weakens cellular adhesion, which—through altered lamina–chromatin interactions—down-regulates adhesion-related genes (such as integrin alpha-4 [ITGA4]) and mechanotransduction genes. Consequently, reduced nuclear mechanosensitivity establishes a self-reinforcing feedback loop that further adaptively diminishes nuclear mechanical responsiveness. This loop unveils a fundamental mechanism by which the nucleus dynamically calibrates cellular mechanical responses through SUN-mediated transcriptional control of adhesion and mechanosensing programs.

## Results

### Knockout *Sun1* or *Sun2* decreases mechanotransduction-related gene expression in myoblasts

**Fig. 1.**
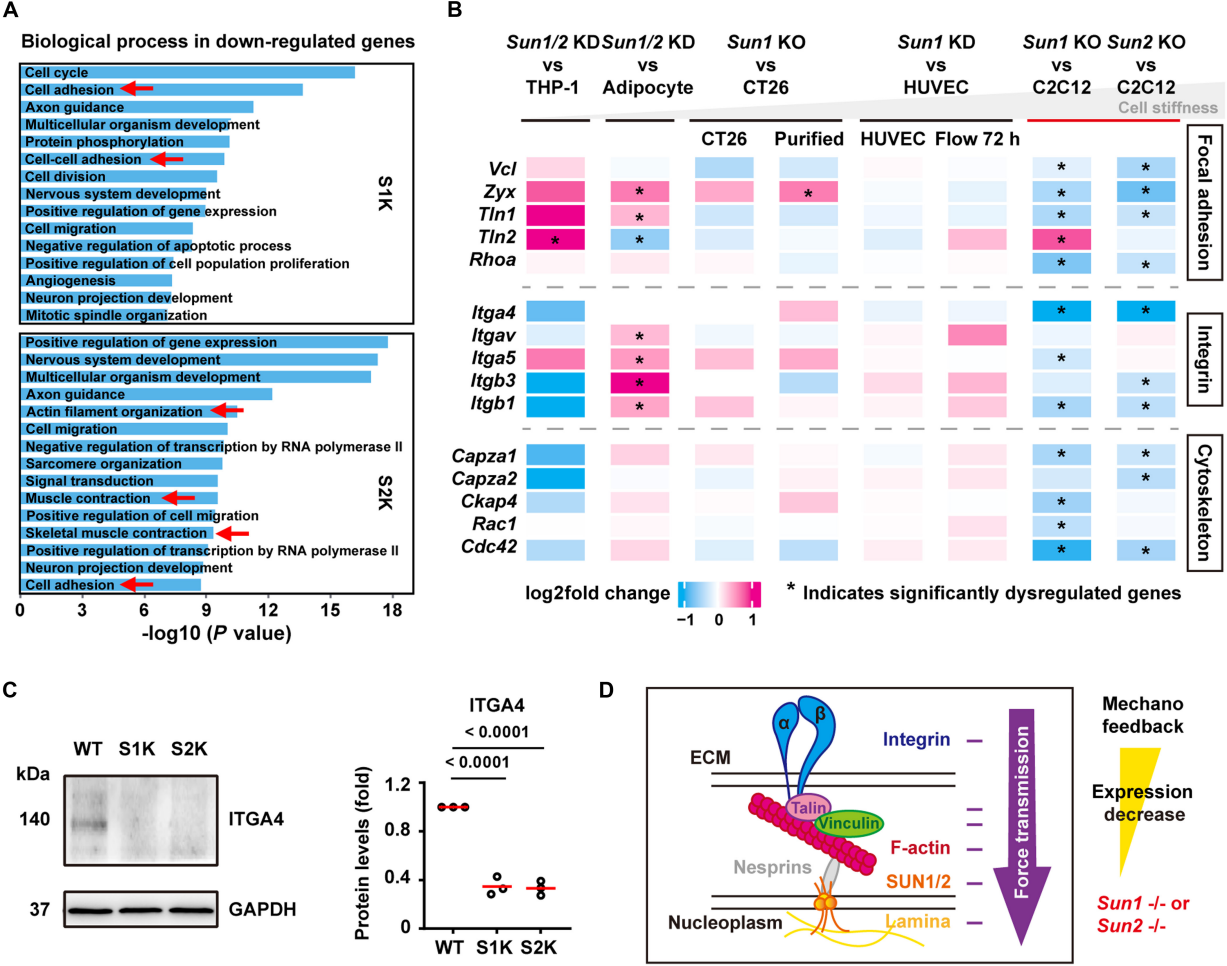
Loss of SUN1 or SUN2 suppresses the upstream mechanotransduction genes expression. (A) Gene ontology (GO) functional enrichment terms of significantly down-regulated genes after knocking out *Sun1* or *Sun2*. Only the top 15 biological process terms are shown for each group. Red arrows indicate cell adhesion and cell force-related terms. (B) The heatmap shows the expression change levels of focal adhesion-related, integrin-related, and cytoskeleton-related genes. Asterisks indicate significantly dysregulated genes (FDR < 0.05 and fold change ≥ 1.2). The cell stiffness from left to right is greater (gray; THP-1 [65], CT26 [66], HUVEC [67], Adipocyte < C2C12 [39]). (C) WB analysis of the representative down-regulated gene of ITGA4 protein levels in *Sun1* or *Sun2* knockout group (3 biological replicates per group). Quantitative data to the right are presented as the mean with SD. One-way analysis of variance (ANOVA) and Tukey’s HSD test. WT, wild-type C2C12 cells; S1K, *Sun1* KO C2C12 cells; S2K, *Sun2* KO C2C12 cells. (D) Schematic model of the nuclear mechano-feedback pathway from the extracellular matrix to the nucleus. In this model, knockout of *Sun1* or *Sun2* inhibits the expression of upstream mechanotransduction genes, and the pathway involves integrins, talin, vinculin, F-actin, nesprins, SUN proteins, and lamins.

The classical model of cellular mechanosensing and mechanotransduction, centered on the integrin–FAK–actin–LINC–lamina axis, represents a fundamental pathway through which cells perceive and adapt to external mechanical stress [21,34,35]. To elucidate the essential roles of SUN1 and SUN2 as mechanical conduits in force transmission to chromatin and their regulatory functions in mechanotransduction, we employed C2C12 myoblasts as a model system. Using CRISPR/Cas9-mediated gene editing, we successfully generated stable *Sun1* knockout (KO) and *Sun2* KO C2C12 cell lines (Fig. S1A and B). RNA sequencing (RNA-seq) analysis performed on wild-type (WT), *Sun1* KO, and *Sun2* KO cells revealed that depletion of either *Sun1* or *Sun2* had substantial alterations in the transcriptome (Fig. S2A, B, G and H). Specifically, gene ontology (GO) analysis indicated significant down-regulation of terms associated with cell adhesion and muscle contraction (Fig. 1A). Notably, *Sun2* KO resulted in down-regulation of pathways related to actin filament organization, muscle contraction, and skeletal muscle contraction.

FA, the dynamic structure formed via specific molecular interactions between cells and the extracellular matrix, is the core structure for cell mechanical sensing and transduction [36,37]. These connections are mainly mediated by integrins, which bridge extracellular matrix proteins and the intracellular cytoskeleton [38]. Among the cell types analyzed, C2C12 myoblasts exhibit relatively high cellular stiffness as reported in previous studies based on atomic force microscopy (AFM) measurements of the Young’s modulus [39]. Consistent with this mechanical property, cellular deformability assessed using a microfluidic constriction device (Fig. S3A to C) revealed that C2C12 WT cells traversed the channels significantly more slowly than *Sun1* KO and *Sun2* KO cells (Fig. S3D), indicating increased deformability upon loss of SUN1/2. In C2C12 cells, KO of *Sun1* or *Sun2* markedly reduces the expression of genes encoding integrins (e.g., *Itga4*, *Itga5*, *Itgb3*, and *Itgb1*), adhesion complex components (e.g., *Vcl*, *Zyx*, *Tln1*, and *Rhoa*), and cytoskeleton-regulated factors (e.g., *Capza1*, *Capza2*, *Ckap4*, *Rac1*, and *Cdc42*) (Fig. 1B). Quantitative reverse transcription PCR (RT-qPCR) validation of the key genes *Itga4*, *Zyx*, *Rhoa*, and *Cdc42* yielded results highly consistent with RNA-seq data (Fig. S2C to F). Among these, ITGA4 exhibited the most reduction at the protein level (Fig. 1C). Crucially, to validate this SUN-dependent adhesion mechanism, we examined primary mouse mesenchymal stem cells (MSCs) with high hardness [39]. Consistent with our findings in C2C12 myoblasts, siRNA-mediated knockdown of *Sun1* or *Sun2* (Fig. S4A to D) similarly resulted in significant down-regulation of key adhesion-related genes (*Itga4* and *Cdc42*) in MSCs (Fig. S5A and B). To establish causality and exclude potential off-target effects, we performed rescue experiments by overexpressing *Sun1* in *Sun1* KO cells and *Sun2* in *Sun2* KO cells, respectively (Fig. S6A to F). This re-expression rescued the down-regulation of adhesion genes, including *Rhoa*, *Cdc42*, and *Itga4* (Fig. S6G to L).

In contrast, in cell types reported to exhibit lower cellular stiffness or higher deformability, such as the circulating THP-1 cells [40], adipogenic cells [9], HUVEC [41], and CT26 [42], depletion or reduction of SUN1 or SUN2 resulted in minimal changes in adhesion-related gene expression, and certain adhesive genes were even up-regulated in soft adipocytes (Fig. 1B). These observations suggest that SUN1/2-mediated nuclear mechanotransduction may attenuate the expression of upstream mechanosensing and mechanotransduction genes via a feedback mechanism, particularly in stiffer cellular contexts like C2C12 myoblasts, thereby recalibrating nuclear mechanical force homeostasis (Fig. 1D).

**Fig. 2.**
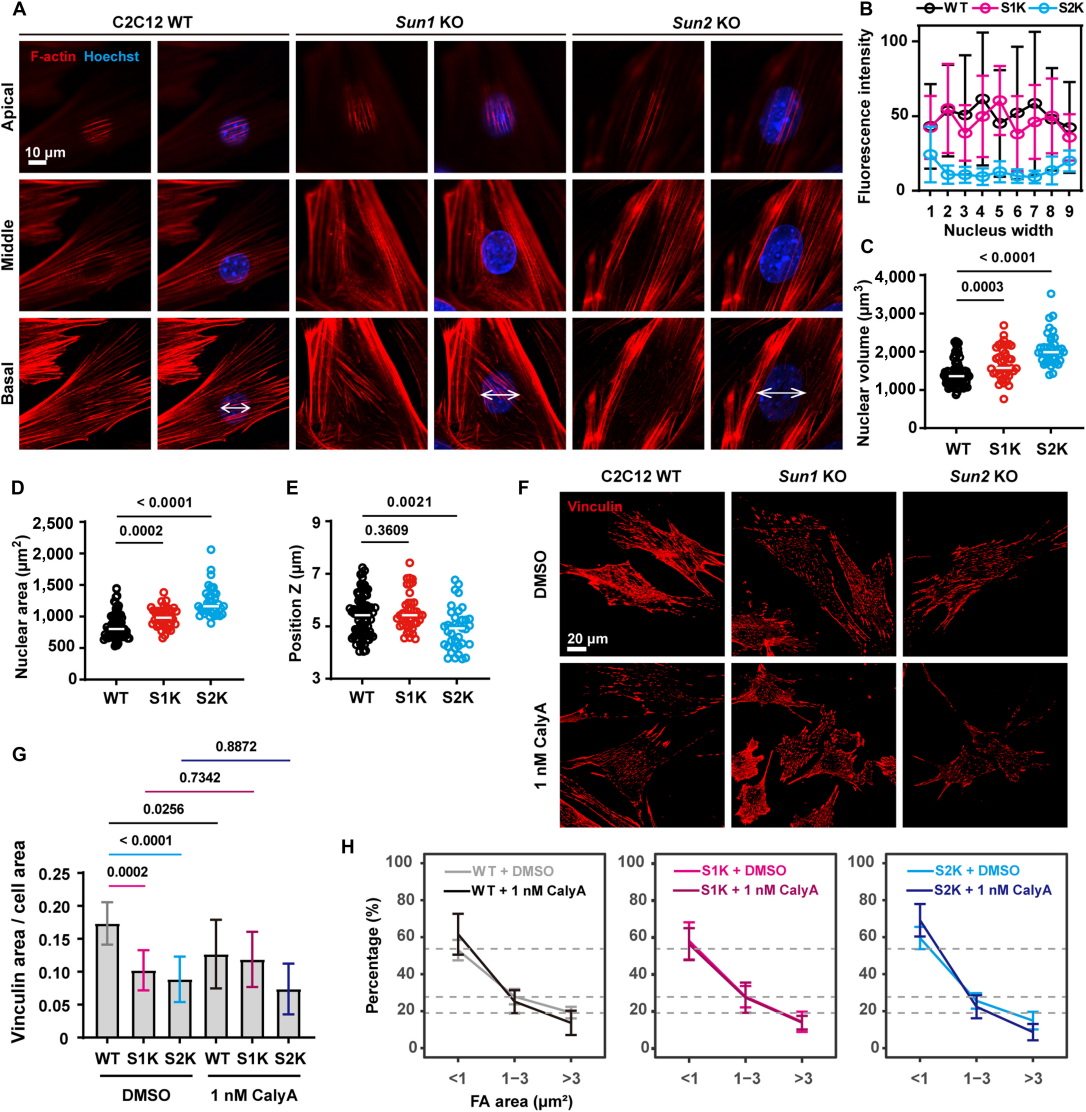
SUN1 or SUN2 loss disrupts actin and inhibits focal adhesion (FA). (A) Immunofluorescence staining and imaging of nuclei and F-actin in C2C12 WT, *Sun1* knockout (KO), and *Sun2* KO cells (nuclei, *n* = 81, 44, and 37 cells; F-actin, *n* = 7, 7, and 7 cells). Images were taken from the apical, middle, and basal planes of the cells. Scale bar, 10 μm. (B) Line graph showing the quantitative distribution of F-actin fluorescence intensity within the white line region in (A). The *x*-axis represents 9 equal divisions of the measured region. Data are presented as the mean with SD. (C to E) Quantitative analysis of nuclear volume (C), nuclear area (D), and nuclear height (E). Each dot represents an individual cell, and the white line segment indicates the mean value. One-way ANOVA and Tukey’s HSD test. (F) Immunofluorescence staining and imaging of vinculin in C2C12 WT, *Sun1* KO, and *Sun2* KO cells (*n* = 9, 17, and 10 cells) and cells treated with 1 nM Calyculin A for 20 min (*n* = 19, 18, and 18 cells). Scale bar, 20 μm. (G) Quantitative analysis of density of the FA, i.e., the total area of the vinculin divided by the cell area in each cell. Data are presented as the mean with SD. One-way ANOVA and Tukey’s HSD test. (H) Quantitative analysis of the proportion of FA with different volumes (<1, 1 to 3, and >3 μm^2^) in each cell. Data are presented as the mean with SD. Gray dashed lines in 3 plots indicate the mean percentage of FA area in the WT dimethyl sulfoxide (DMSO) group. S1K represents the *Sun1* KO cell, and S2K represents the *Sun2* KO cell in all the figures.

To further validate the mechanical feedback phenomenon, we performed immunofluorescence staining for F-actin and chromatin DNA to visualize the cytoskeleton and nuclei in C2C12 WT, *Sun1* KO, and *Sun*2 KO cells. The distribution of perinuclear F-actin was examined across distinct cellular layers: the apical, middle, and basal regions. In WT cells, perinuclear F-actin exhibited a high-density, parallel arrangement, while *Sun1* KO cells displayed disorganized perinuclear F-actin in the basal layer, which appeared misaligned relative to other layers. *Sun2* KO cells showed a more severe phenotype, with nearly absent perinuclear F-actin structures (Fig. 2A). To quantitatively assess these cytoskeletal alterations, we divided the basal view of the nucleus into 9 equal segments and analyzed the perinuclear distribution of F-actin (Fig. 2B). Our results revealed a substantial reduction in basal perinuclear F-actin density in *Sun2* KO cells, accompanied by increased distance between F-actin and the nuclear envelope, which were corroborated through orthogonal views along the *x*–*z* and *y*–*z* axes (Fig. S7). We also observed that KO of either *Sun1* or *Sun2* led to significant increases in nuclear volume and area (Fig. 2C and D), and *Sun2* KO resulted in noticeable nucleus flattening (Fig. 2E).

We next investigated the impact of SUN1/2 depletion on FA by examining vinculin localization via immunofluorescence (Fig. 2F). Quantitative analysis revealed a decrease in the density of vinculin in both *Sun1* KO and *Sun2* KO cells compared to WT controls (Fig. 2G), consistent with decreased adhesion (Fig. S8). Additionally, SUN1/2 deficiency increased the proportion of small vinculin-positive foci (<1 μm^2^) (Fig. 2H). To probe the functional contractile response, we treated WT, *Sun1* KO, and *Sun2* KO cells with 1 nM Calyculin A (CalyA)—an inhibitor of protein phosphatases PP1 and PP2A that induces actomyosin contraction through myosin light chain (MLC) phosphorylation [43]. After 20 min of treatment, vinculin staining was used to evaluate FA density and size distribution (Fig. 2F to H). While CalyA markedly reduced FA density and increased the abundance of small FAs (<1 μm^2^) in WT cells, neither *Sun1* KO nor *Sun2* KO cells exhibited notable changes in FA morphology following treatment, indicating an impaired contractile response.

Collectively, these results demonstrate that SUN1/2 deficiency disrupts FA organization, cytoskeletal architecture, and nuclear morphology, underscoring its essential role in mechanotransduction and mechanical feedback regulation.

### SUN1 and SUN2 regulate perinuclear chromatin dynamic distribution

**Fig. 3.**
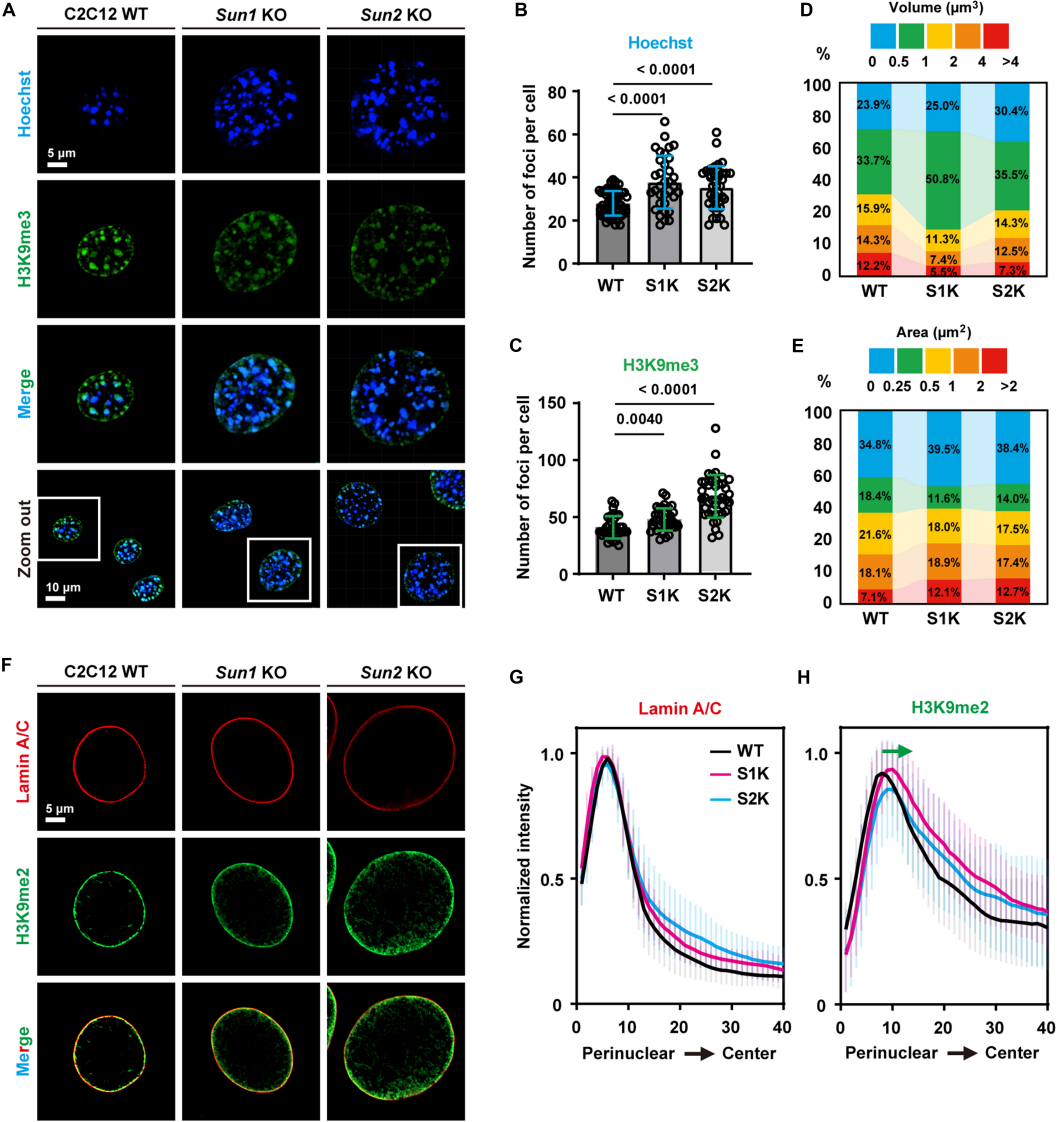
*Sun1* or *Sun2* knockout regulates heterochromatic distribution. (A) Immunofluorescence staining and imaging of the nuclei and H3K9me3 in C2C12 WT, *Sun1* KO, and *Sun2* KO cells (nuclei, *n* = 58, 34, and 39 cells; H3K9me3, *n* = 34, 38, and 41 cells). Scale bars, 5 and 10 μm. (B and C) Quantitative analysis of the number of Hoechst-foci in 3D (B) and H3K9me3-foci in 2D (C) per cell. Each dot represents an individual cell. Data are presented as the mean with SD. One-way ANOVA and Tukey’s HSD test. (D and E) Quantitative analysis of the proportion of Hoechst-foci (D) and H3K9me3-foci (E) with different volumes or areas in each cell. (F) Immunofluorescence staining and imaging of the lamin A/C and H3K9me2 in C2C12 WT, *Sun1* KO, and *Sun2* KO cells (lamin A/C, *n* = 50, 40, and 45 cells; H3K9me2, *n* = 50, 40, and 45 cells). Scale bar, 5 μm. (G and H) Line graph showing the distribution of lamin A/C (G) and H3K9me2 (H) fluorescence intensity across the nuclear periphery. Using the lamin A/C as the nuclear periphery boundary, the H3K9me2 displacement in *Sun1/2* KO cells was determined. The *x*-axis divides the segmented nuclear periphery to center (40 pixels). Data are presented as the mean with SD. The green arrow indicates the displacement phenomen. S1K represents the *Sun1* KO cell, and S2K represents the *Sun2* KO cell in all the figures.

Given the significant alterations in nuclear morphology and structure observed in *Sun1* KO and *Sun2* KO cells, we hypothesized that these changes might be associated with concomitant alterations in chromatin organization and histone modification. The trimethylation of histone H3 at lysine 9 (H3K9me3) serves as a well-established marker of heterochromatin, which often colocalizes with dense DAPI-stained regions, typically enriched at the nucleus periphery and around nucleoli [44]. To investigate this, we performed immunofluorescence staining for H3K9me3 and analyzed the resulting distribution patterns. The results showed that compared with C2C12 WT, the number of heterochromatic foci significantly increased in *Sun2* KO cells (Fig. 3A to C), while the size of heterochromatic foci was reduced in *Sun1* KO cells, with a predominant distribution in the 0.5 to 1 μm^3^ or 0 to 0.25 μm^2^ range (Fig. 3D and E). These findings indicate that the loss of SUN1 or SUN2 disrupts the stability and nuclear organization of H3K9me3-marked heterochromatin, leading to its dissociation or fragmentation. Furthermore, we observed a notable redistribution of H3K9me2, another heterochromatin-associated mark, from the nuclear periphery toward the interior following *Sun1/2* depletion (Fig. 3F to H). This suggests a broader disruption of peripheral heterochromatin architecture. Consistent with these morphological changes, expression analysis of key enzymes regulating H3K9 methylation states showed a down-regulation of genes encoding H3K9 methyltransferases and an up-regulation of those involved in H3K9 demethylation (Fig. S9A and B), further supporting the conclusion that SUN1/2 deficiency induces heterochromatic reorganization globally.

**Fig. 4.**
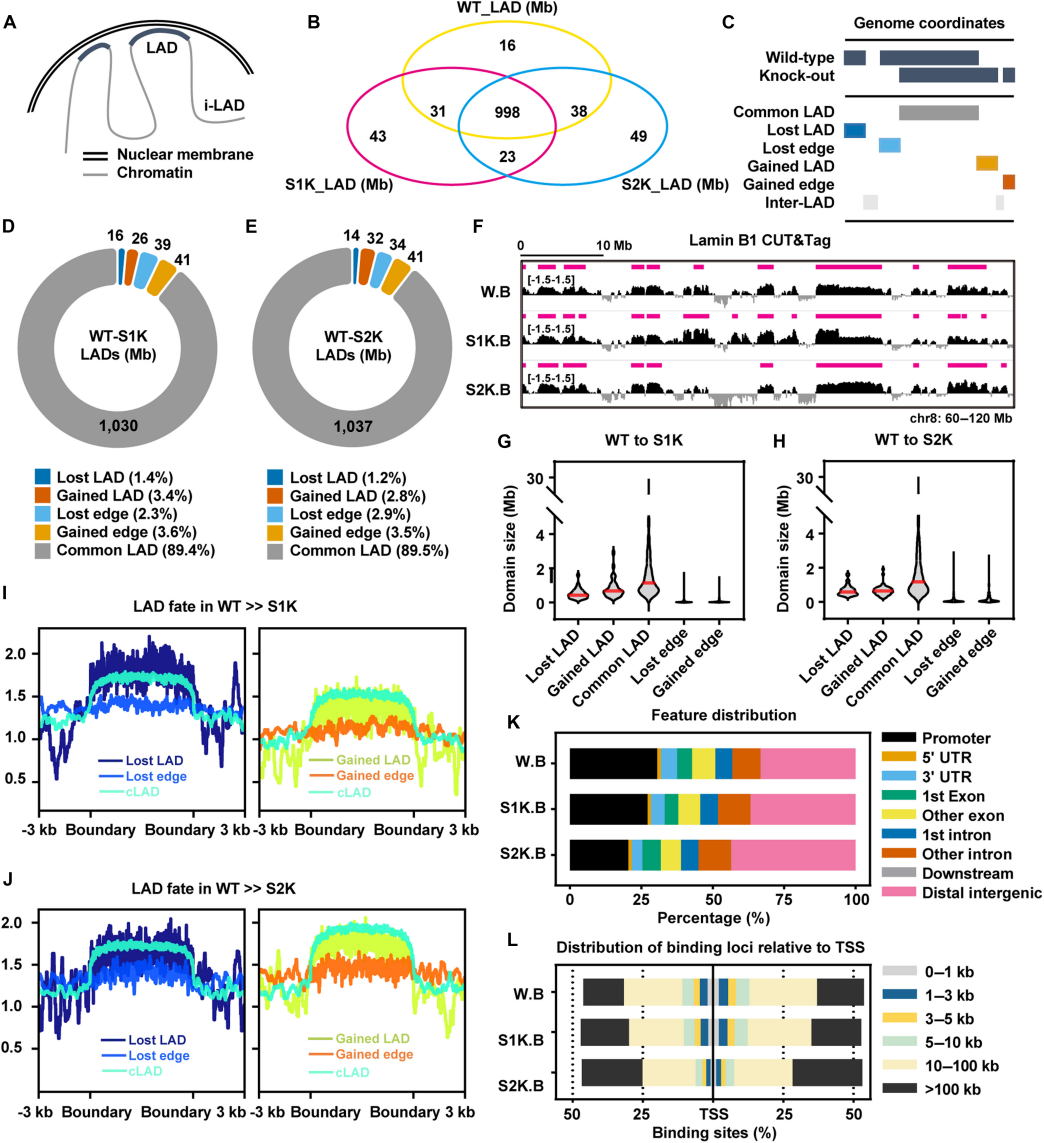
LAD features after *Sun1* or *Sun2* knockout. (A) Schematic representation of the distribution of LAD and i-LAD around the nuclear periphery. (B) Venn diagram of genome coverage by LADs in C2C12 WT cells, *Sun1* KO cells, and *Sun2* KO cells. The unit is Mb. (C) Definitions of 5 categories of LAD and i-LAD. (D and E) Changes in the total length of 5 types of LADs, compared to C2C12 WT cells, in *Sun1* KO cells (WT-S1K) (D) and in *Sun2* KO cells (WT-S2K) (E). The unit is Mb, and the percentage in the legend represents the corresponding proportion of change. (F) Gene views showing lamin B1 signals at chromosome 8 positions 60 to 120 Mb in C2C12 WT cells, *Sun1* KO cells, and *Sun2* KO cells. Red bars indicate identified LAD. (G and H) Violin plots showing the domain size of 5 categories of LAD, comparing C2C12 WT cells to *Sun1* KO cells (WT to S1K) (G) and comparing C2C12 WT cells to *Sun2* KO cells (WT to S2K) (H). Red line segments represent the mean values. (I and J) Distribution of lamin B1 signals within LAD and their boundaries (±3 kb upstream/downstream) in (left) C2C12 WT cells and (right) *Sun1* KO cells (H) and in (left) C2C12 WT cells and (right) *Sun2* KO cells (I). (K) Percentage distribution of gene features associated with lamin B1 binding sites in C2C12 WT cells, *Sun1* KO cells, and *Sun2* KO cells. Gene features include promoters, UTRs, exons, introns, and distal intergenic regions. (L) Distribution of distances between lamin B1 binding sites and the nearest transcription start site. The genomic direction is 5′→3′. W.B represents lamin B1 binding sites in C2C12 WT cells, S1K.B represents lamin B1 binding sites in *Sun1* KO cells, and S2K.B represents lamin B1 binding sites in *Sun2* KO cells in all the figures.

Heterochromatin always interacts with the nuclear lamina proteins, forming structures known as LADs. Genes embedded within LADs are typically transcriptionally repressed due to the peripheral localization and repressive chromatin environment [30], while other regions outside LADs, referred to as inter-LADs (i-LADs), are generally associated with a more open chromatin state and active transcription [45] (Fig. 4A). To elucidate the role of SUN1/2 in genome organization, we performed lamin B1 CUT&Tag (cleavage under targets and tagmentation) sequencing in C2C12 WT, *Sun1* KO, and *Sun2* KO cells. LADs were identified in each group from the merged domains between 2 replicates and the intersection of LADs reveals 998 megabases (Mb) of constitutive LADs, and some variable LADs were unique to *Sun1* KO and *Sun2* KO cells (Fig. 4B). We then categorized the changes in LAD architecture following *Sun1/2* depletion into 5 distinct classes: Common LAD (cLAD), Lost LAD, Lost Edge, Gained LAD, and Gained Edge (Fig. 4C). The results showed that *Sun1* KO led to an increase of 26 Mb in LAD and 41 Mb in LAD edge, while *Sun2* KO resulted in a gain of 32 Mb in LAD and 41 Mb in LAD edge. However, the loss of LAD and LAD edge was relatively less marked: a reduction of 16 Mb in LAD and 39 Mb in LAD edge was observed after *Sun1* KO, and a decrease of 14 Mb in LAD and 34 Mb in LAD edge after *Sun2* KO. Therefore, knocking out *Sun1* or *Sun2* resulted in a change in LAD composition and the acquisition of more LAD (Fig. 4D to F). We further analyze the length characteristics of the 5 types of LADs in each cell line. Compared with cLAD, the acquired or lost type of whole LAD or edge type LAD are smaller, indicating that they are more prone to unstable interactions with the nuclear lamina [46]. Notably, compared to *Sun1* KO, *Sun2* KO resulted in smaller gained LAD yet larger gained and lost edges (Fig. 4G and H), highlighting isoform-specific regulatory roles in domain stability and boundary definition.

We next examined lamin B1 binding intensity across the different LAD categories. In *Sun1* KO cells, lost LADs exhibited a more convex binding profile, indicating pronounced disruption. Although cLAD is a positional invariant LAD, we observed a substantial decrease in lamin B1 affinity for chromatin on cLAD, and lamin B1 enriched on the gained LAD or gained edge is less than that on the lost LAD or lost edge after the loss of SUN1, while loss of SUN2 is the opposite (Fig. 4I and J). The above results indicate that *Sun1* KO and *Sun2* KO have different characteristics of LAD composition changes. To understand the functional implications of these structural changes, we analyzed the genomic features of LADs. After knocking out *Sun1*/*2*, LADs showed a decreased association with promoter regions and were redistributed to more distal genomic areas (Fig. 4K). Specifically, we detected a loss of sequences within 10 kb of transcription start sites (TSS) and a concomitant gain of sequences over 100 kb away from TSSs (Fig. 4L). We then zoomed in on the genomic locus of the key mechanosensing genes *Rhoa* and *Cdc42*, and we observed a gain of lamin B1 signal specifically covering the gene regulatory elements in *Sun1* or *Sun2* KO cells (Fig. S10); this result is consistent with the low expression of *Rhoa* and *Cdc42* in Fig. S2. Consistent with these locus-specific observations, enrichment analysis revealed that down-regulated genes are significantly more likely to gain lamin B1 association compared with nondifferentially expressed genes (Fisher’s exact test, *P* < 0.05, Fig. S11A and B). These findings demonstrate that SUN1 and SUN2 are critical regulators of LAD genomic positioning and connectivity, thereby influencing long-range chromatin organization and gene regulation.

### Lamin A/C acts as the key regulator in adhesion gene expression via LAD reorganization

**Fig. 5.**
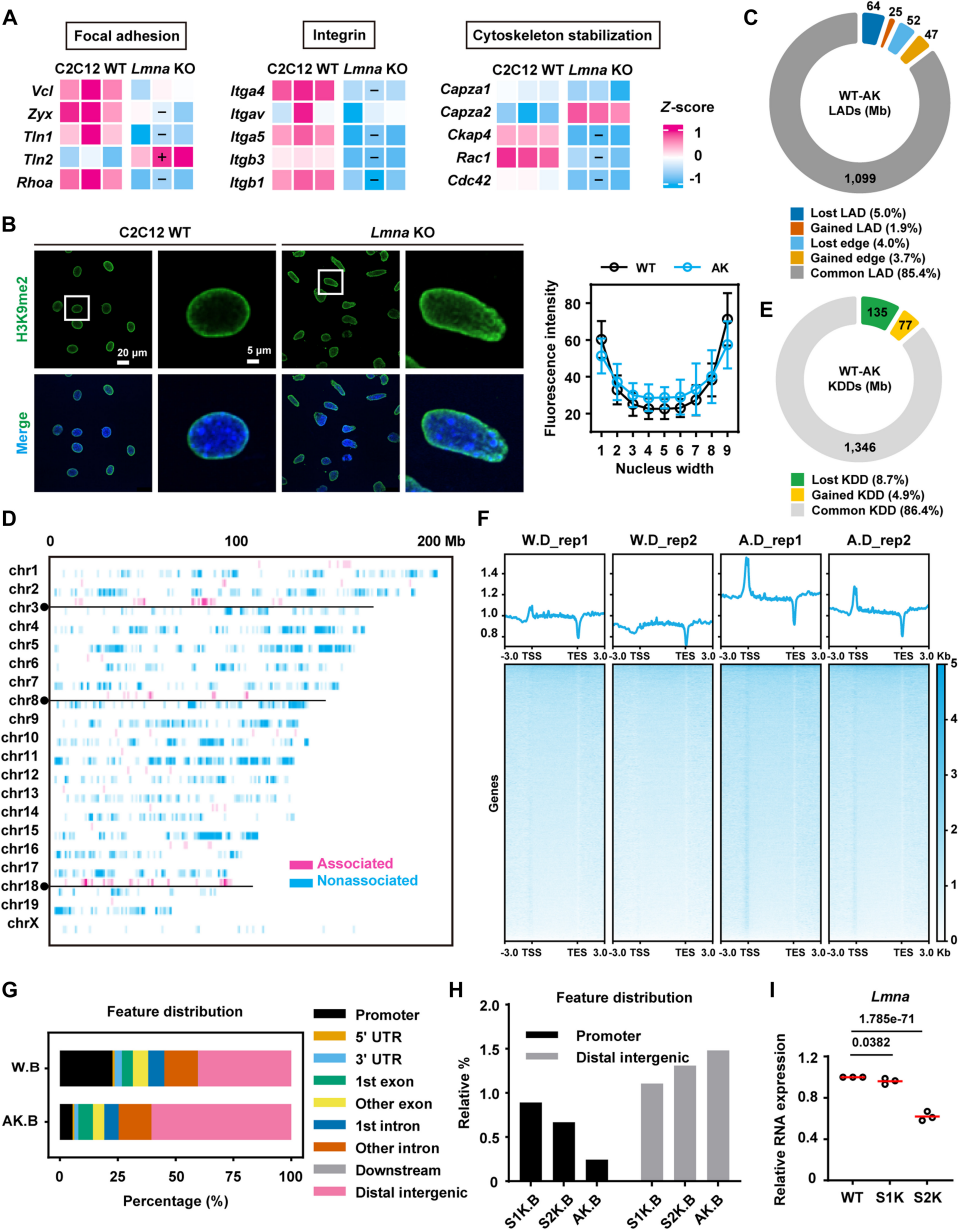
*Lmna* knockout decreases cell adhesion genes expression and LAD regulation. (A) The heatmap shows the relative expression levels of focal adhesion-related, integrin-related, and cytoskeleton-related genes in C2C12 WT, and *Lmna* KO cells (3 biological replicates per group). Each row is standardized using the *Z*-score method. “+” indicates significantly up-regulated genes, and “−” indicates significantly down-regulated genes. (B) Immunofluorescence staining and imaging of the nucleus and H3K9me2 in C2C12 WT and *Lmna* KO cells (H3K9me3, *n* = 23 and 26 cells). Scale bars, 20 μm (left) and 5 μm (right). Line graph showing H3K9me2 fluorescence distribution within the nucleus. The *x*-axis divides the nuclear diameter into 9 equal parts. Data are presented as the mean with SD. (C) Compared to C2C12 WT, the total length of 5 categories of LAD in *Lmna* KO cells (WT-AK) has changed, measured in Mb. The percentages on the legend represent the corresponding proportional changes. (D) After knocking out *Lmna*, a heatmap shows significant changes in lamin B1 binding affinity at chromosomal locations. Red indicates regions with significantly increased binding affinity, while blue indicates regions with significantly decreased binding affinity. (E) Compared to C2C12 WT, the total length of 3 classes of KDD in *Lmna* KO cells (WT-AK). (F) The line graph above shows the total signal distribution of H3K9me2 across the entire genome and its up/downstream 3 kb regions in C2C12 WT and *Lmna* KO cells (2 replicates per group). Below, the heatmap displays the H3K9me2 signal values around each gene, sorted from highest to lowest. (G) Percentage distribution of gene characteristics associated with lamin B1 binding sites in C2C12 WT and *Lmna* KO cells. Gene characteristics include promoters, UTRs, exons, introns, and distal intergenic regions, among others. (H) Relative percentage distribution of gene characteristics associated with lamin B1 binding sites in *Sun1* KO, *Sun2* KO, and *Lmna* KO cells compared to C2C12 WT cells. The most altered gene characteristics were displayed including promoters and distal intergenic regions. (I) Quantification of *Lmna* RNA transcription levels across the 3 cell groups in RNA-seq data. Each dot represents a biological replicate, and red line segments indicate the mean values. W.D represents H3K9me2 binding sites in C2C12 WT cells, AK.B represents lamin B1 binding sites in *Lmna* KO cells, S1K.B represents lamin B1 binding sites in *Sun1* KO cells, and S2K.B represents lamin B1 binding sites in *Sun2* KO cells in all the figures.

Since SUN1/2 engages with lamin A through its N-terminal domain [22], we sought to determine whether lamin A deficiency impairs mechanotransduction and mechanical feedback regulation. To further elucidate the role of lamin A/C in LAD regulation, we performed transcriptomic analysis and observed significant down-regulation of cell adhesion-related genes after knocking out *Lmna* (Fig. 5A and Fig. S12) [47]. Given that heterochromatin histone modification H3K9me2 is associated with transcriptional repression [48], active histone modification H3K4me1 marks active gene expression [49]. Thus, we hypothesized that lamin A/C may influence LAD-driven gene expression by modulating local chromatin states. Using immunofluorescence, we examined the distribution of H3K9me2 and H3K4me1 in C2C12 WT and *Lmna* KO cells. *Lmna* depletion led to a redistribution of H3K9me2 from nuclear periphery toward the interior (Fig. 5B), suggesting disrupted heterochromatin anchoring. In contrast, the average fluorescence intensity of H3K4me1 remained unchanged. These epigenetic alterations were accompanied by severe nuclear deformation and loss of perinuclear lamin B1 staining after knocking out *Lmna* (Fig. S13A and B), indicating broad compromise of nuclear envelope integrity and chromatin organization in the absence of lamin A/C.

To further investigate the hierarchical regulation of the SUN1/2-lamin A/C-LAD axis, we performed lamin B1 CUT&Tag sequencing in C2C12 WT and *Lmna* KO cells. The results revealed a substantial reorganization of LAD architecture: *Lmna* KO led to a loss of 64 Mb in LAD and 52 Mb in LAD edge regions, while also triggering a gain of 25 Mb in LAD and 47 Mb in LAD edge (Fig. 5C). Consistent with this structural shift, lamin B1 enrichment across chromatin was dramatically reduced in *Lmna* KO cells compared to WT controls (Fig. 5D), indicating that lamin A/C deficiency disrupts LAD integrity and promotes LAD loss.

We further analyzed the epigenetic and genomic features of LADs. In *Lmna* KO cells, 525 loci with significantly increased lamin B1 affinity were unrelated to genes, while 1,104 and 925 loci were linked to one or multiple genes, respectively, mainly distributed > 50 kb from TSS (Fig. S14A and B). Conversely, 8,413 loci with reduced lamin B1 affinity were gene-associated primarily situated within 5 kb of TSS (Fig. S14C and D). These findings indicate that *Lmna* depletion shifts LADs from promoter-proximal to distal genomic regions and diminishes gene-associated sequences near TSS, paralleling phenotypes observed in *Sun1/2* KO models. Although genes within LADs consistently exhibited low expression levels in both WT and *Lmna* KO cells (Fig. S14E), consistent with the repressive nature of LADs reported by previous studies [32], the overall correlation between LAD positioning and differential gene expression was negative but not strictly deterministic (Fig. S14F and G) [30,46], suggesting that additional epigenetic mechanisms may also be involved in influencing transcriptional output.

To assess the role of histone modifications, we performed CUT&Tag for H3K9me2 (defining heterochromatic KDD domains) and H3K4me1 (marking active KMD domains) in WT and *Lmna* KO cells. *Lmna* KO resulted in a loss of KDD (135 Mb) (Fig. 5E), yet increased global H3K9me2 levels and slightly elevated its promoter binding (Fig. 5F and Fig. S15). Conversely, KMD sites increased (6,677 loci), but H3K4me1 modification at promoters decreased (Fig. S16A to C). Principal component analysis (PCA) revealed that upon *Lmna* KO, repressive KDD domains diverged from LADs, while active KMD domains converged toward them (Fig. S17). We zoomed in on the genomic locus of *Rhoa* and *Cdc42* and observe that these loci gain repressive H3K9me2 and lose active H3K4me1 (Fig. S18). This reconfiguration with elevated H3K9me2 and reduced H3K4me1 aligns with the overall down-regulation of gene expression, underscoring the role of histone modifications in mediating transcriptional responses to lamin A loss.

Further, we compared the LAD genomic positioning among WT, *Sun1* KO, *Sun2* KO, and *Lmna* KO cells, and identified the similarity of their distribution in promoter regions and distal genomic areas; the LADs in *Sun2* KO and *Lmna* KO cells showed more similar changes in the decreased association with promoter regions and more distal intergenic areas (Fig. 5G and H), and *Sun2* KO dramatically reduced lamin A expression, but only had a minor effect in the *Sun1* KO group (Fig. 5I), indicating that there are more functional correlations between SUN2 and lamin A, implying their hierarchical regulation of the SUN2–lamin A/C–LAD axis.

### SUN1/2 and their direct downstream protein lamin A/C prime myogenic differentiation

**Fig. 6.**
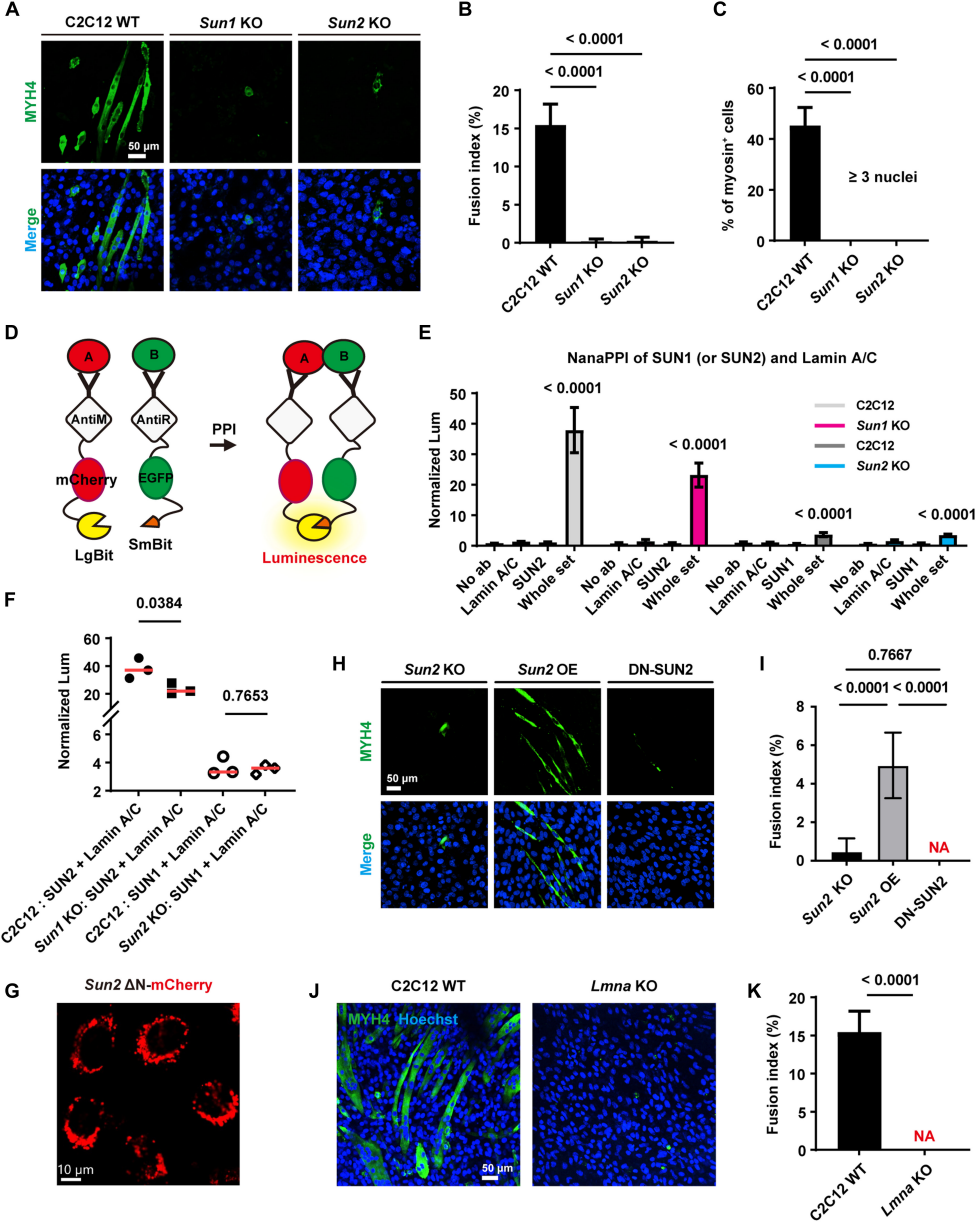
Loss of cell differentiation function after knocking out of *Sun1*, *Sun2*, or the downstream *Lmna*. (A) Immunofluorescence staining and imaging of nuclei and MYH4 in C2C12 WT, *Sun1* KO, and *Sun2* KO cells (*n* = 5, 5, and 5 views). Scale bar, 50 μm. (B) Quantitative analysis of the ratio of fused myotube nuclei to total nuclei in each group. Data are presented as the mean with SD. One-way ANOVA and Tukey’s HSD test. (C) Quantitative analysis of the ratio of nuclei within individual myotubes (nuclei count > 3) to total nuclei across all myotubes. Data are presented as the mean with SD. One-way ANOVA and Tukey’s HSD test. (D) Schematic diagram of the NanaPPI design. (E) Quantitative analysis of the interaction between lamin A/C and SUN1 or SUN2 protein, after *Sun2* or *Sun1* knockout, respectively. Three biological replicates per group. Data are presented as the mean with SD. Two-tailed unpaired Student *t* tests. (F) Quantitative analysis of differences in protein interactions. The *x*-axis represents different PPI pairs across cells. Each dot represents a biological replicate; the red line segment represents the mean value. Two-tailed unpaired Student *t* tests. (G) Representative fluorescence images of *Sun2* KO cells stably expressing the mCherry-tagged dominant-negative mutant SUN2 (201-731, DN-SUN2) via viral infection. Scale bar, 10 μm. (H) Immunofluorescence staining and imaging of nuclei and MYH4 in *Sun2* KO, *Sun2* OE, and DN-SUN2 cells (*n* = 5, 5, and 5 views). Scale bar, 50 μm. (I) Quantitative analysis of the ratio of fused myotube nuclei to total nuclei in each group. Data are presented as the mean with SD. One-way ANOVA and Tukey’s HSD test. (J) Immunofluorescence staining and imaging of nuclei and MYH4 in C2C12 WT and *Lmna* KO cells (*n* = 5 and 5 views). Scale bar, 50 μm. (K) Quantitative analysis of the proportion of nuclei in fused myotubes relative to total nuclei across each group. Data are presented as the mean with SD. Two-tailed unpaired Student *t* tests.

During the differentiation process from myoblasts to mature muscle fibers [50], cells need to respond to various mechanical cues such as intercellular traction and substrate stiffness to successfully form multinucleated myotubes [51], and the force transmission into the nucleus is critical. To investigate the role of nuclear mechanotransductor proteins SUN1 and SUN2 in muscle cell fate determination, we induced differentiation in C2C12 cells for 6 days and evaluated myotube formation efficiency via heavy chain staining of MYH4 (Fig. 6A). WT C2C12 showed regular myosin striped structures (myotubes), and on average, approximately 15.45% of the total cell population participated in fusion events to form myotubes (Fig. 6B). Among these, multinucleated myotubes (containing more than 3 nuclei) accounted for 45.27% of the fused structures (Fig. 6C). KO of either *Sun1* or *Sun2* severely impaired myogenic fusion. The average proportion of fused myotubes dropped dramatically to 0.17% and 0.26%, respectively, and the formation of multinucleated myotubes was completely abolished. These results demonstrate that SUN1 and SUN2 are essential for myotube differentiation in C2C12 cells, and their loss abrogates the capacity of myoblasts to undergo myogenesis.

Because myogenic differentiation is known to depend on mechanical cues from the extracellular environment, we next examined whether substrate stiffness influences this process and whether SUN proteins are involved in this response. Cells were cultured on tunable polyacrylamide hydrogels to mimic soft tissue (0.2 kPa) or muscle-like stiffness (10 kPa) [52]. As expected, differentiation was strongly dependent on substrate stiffness. WT cells differentiated efficiently on the 10-kPa substrates but showed markedly reduced differentiation on the soft 0.2-kPa gels (Fig. S19A to C). Under the permissive 10-kPa condition, *Sun2* KO cells displayed significantly impaired differentiation, whereas *Sun1* KO cells showed almost complete loss of myotube formation. On soft substrates, however, myotube formation was largely suppressed in all cell lines, including WT cells. Together, these findings indicate that myogenic differentiation is dependent on substrate stiffness and that SUN1 and SUN2 are required for efficient differentiation under mechanically permissive conditions, supporting a role for SUN proteins in cellular responses to matrix mechanical cues.

Given that SUN proteins are known to interact with lamin A/C via their N-terminal domains [22], and considering that lamin A/C directly associates with LADs to influence chromatin organization and gene expression [53], we proposed that lamin A/C may serve as a critical downstream mediator through which SUN proteins exert regulatory control over LAD organization. To investigate whether lamin A/C participates in the hierarchical regulation axis of SUN1/2-LAD, we first performed immunofluorescence staining for lamin A/C in C2C12 WT, *Sun1* KO, and *Sun2* KO cells. We observed a redistribution of lamin A/C away from the nuclear periphery following the KO of either *Sun1* or *Sun2* (Fig. S20A to C), but there was no significant change in total protein levels of lamin A, lamin C, or total lamin A/C (Fig. S20D to G), suggesting that SUN1/2 deficiency specifically disrupts the subnuclear localization of lamin A/C rather than its expression.

Then, using NanaPPI (nanobody-assisted nanoluciferase fragment complementation for in situ measurement and visualization of endogenous protein–protein interaction) technology [54] to quantitatively measure protein–protein interaction, the verification results showed that the physical interaction between SUN2 and lamin A/C is greater than that between SUN1 and lamin A/C (Fig. 6D and E). Moreover, SUN1 depletion markedly weakened the SUN2–lamin A/C interaction, whereas SUN2 deficiency had no substantial effect on the SUN1–lamin A/C interaction (Fig. 6F), revealing an asymmetric functional dependency within this mechanotransductory axis. To functionally test whether the specific SUN2–lamin A/C interaction is required for myogenic differentiation, we conducted a functional rescue experiment. We expressed a dominant-negative SUN2 mutant lacking the N-terminal lamin A/C-binding domain (Sun2 ΔN) [55] in *Sun2* KO cells (Fig. S21 and Fig. 6G). Notably, re-expression of WT SUN2 (*Sun2* OE, Fig. S6B) robustly rescued myotube formation, whereas the SUN2 ΔN mutant (DN-SUN2) failed to do so (Fig. 6H and I). The differentiation defect in DN-SUN2 cells was as severe as in the *Sun2* KO cells, with myoblast fusion nearly abolished. These results demonstrate that the direct physical interaction between SUN2 and lamin A/C is necessary for the myogenic differentiation program. To further investigate the effect of the nuclear skeleton protein lamin A/C on the fate of myoblasts, we induced *Lmna* KO C2C12 cells into myogenic differentiation for 6 days. Compared to WT cells, *Lmna* KO cells completely failed to form multinucleated myotubes (Fig. 6J and K), indicating a total loss of myogenic differentiation capacity. This underscores the essential role of lamin A/C in mediating myoblast fusion and fate determination.

## Discussion

**Fig. 7.**
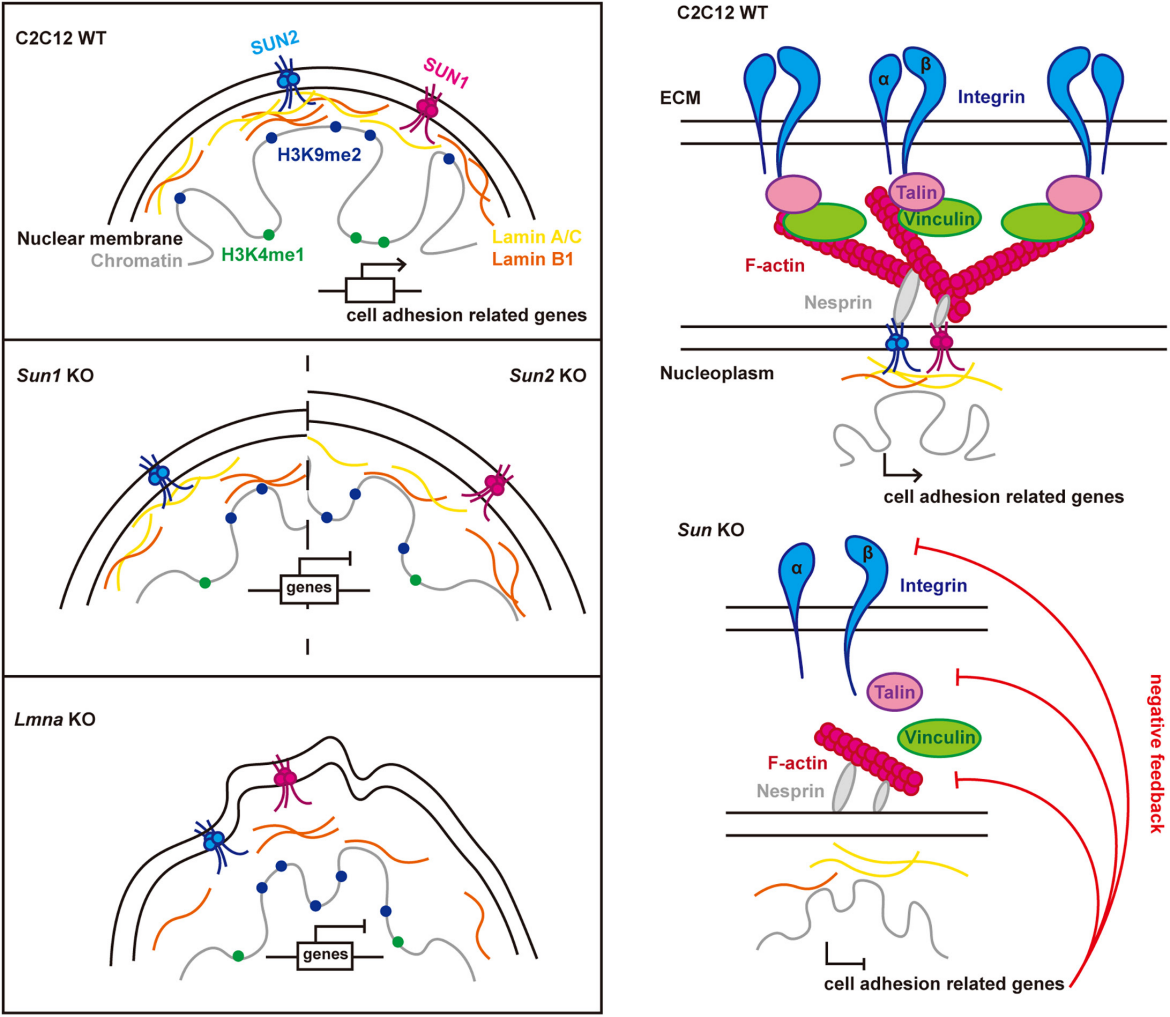
Proposed model for how the nuclear core mechanical protein SUN1/2 and lamin A/C alter cell mechanosensing and mechanotransduction via mechano-feedback and mediate LAD and chromatin reorganization. Knockout of *Sun1*, *Sun2* or *Lmna* alters LAD formation and heterochromatin distribution, thereby inhibiting the gene expression of mechanosensing genes, such as integrin, and mechanotransduction genes, such as focal adhesion (RHOA) and small G protein (CDC42), and then inhibit cell adhesion and fate.

To elucidate whether regulating nuclear mechanotransduction modulates upstream mechanical signaling, this study reveals the role of SUN proteins in nuclear mechanoadaptation: reducing SUN proteins inhibit upstream mechanosensitive gene expression through LAD reorganization and chromatin remodeling, establishing a nuclear mechanical feedback loop (Fig. 7). We discovered that (a) SUN deficiency more severely disrupts mechanosensing and mechanotransduction in high-stiffness cells, e.g., C2C12 cells; (b) SUN1/2 is essential for maintaining nuclear–cytoskeletal integrity; and (c) SUN2 and lamin A/C cooperatively regulate adhesion-related genes via LAD dynamics and histone modifications.

Mechanical feedback is defined as regulatory loops where mechanical cues modulate gene expression to facilitate adaptation to mechanical microenvironment [2,19,20]; however, the core mechanotransduction axis mediating this process remains poorly characterized. In this study, by integrating RNA-seq data with functional observations, we demonstrate that stiffer C2C12 cells exhibit more severe mechanosensing deficits upon SUN1/2 depletion. We also delineate distinct roles for SUN1 and SUN2: SUN1 deficiency causes disordered F-actin alignment, while SUN2 loss leads to a reduction in F-actin content, suggesting roles in stabilizing versus dynamically responding to mechanical inputs. Recent studies have shown that SUN1 is essential for proper cytoskeletal force generation and FA maturation, linking the nucleus to mechanosensing (via integrin beta-1, vinculin, and zyxin) [26,56]. Destruction of actin results in a complete and irreversible disassembly of FA [57], consistent with the decomposition of FA after *Sun1/2* KO. Additionally, SUN1 and SUN2 are vital for RHOA/ROCK-mediated actomyosin activity in vascular smooth muscle cells, indicating a potential feedback circuit between cytoskeletal tension and nuclear components [58]. Collectively, our findings provide a novel paradigm: nuclear mechanotransduction proteins (SUN1/2) modulate cellular mechanoadaptation by establishing a mechano-feedback loop that transcriptionally regulates upstream mechanosensitive genes.

Compared with controls, CalyA-treated C2C12 cells exhibited reduced both cell and nuclear area, along with a significant decrease in FA size. These findings suggest that CalyA enhances actomyosin contractility, accelerating FA turnover and resulting in smaller FAs [59], likely dependent on CalyA treatment concentration and duration [60]. While cells typically maintain mechanical homeostasis via feedback mechanisms that preserve force equilibrium under perturbation [58], SUN-deficient cells fail to respond to actin contractility, indicating loss of adaptive capacity.

Mechanical signaling extends beyond cytoskeletal remodeling into the spatial regulation of chromatin and LADs [53,61]. Our results align with studies showing that perturbation of nuclear envelope proteins disrupts LAD organization and heterochromatin distribution [46,62]. This study leverages the distinct roles of lamin isoforms: using lamin B1 to map the structure of LADs and focusing on lamin A/C as the key mechanoresponsive effector. Critically, we demonstrate that the LADs in *Sun2* KO and *Lmna* KO cells showed more similar changes in the decreased association with promoter regions and more distal intergenic areas, and *Sun2* KO reduced lamin A expression significantly but only had a minor effect in *Sun1* KO cells, which is consistent with our NanaPPI data showing that SUN2 exhibits stronger interaction with lamin A/C than SUN1. While SUN1 deficiency results in “moderate” phenotypes compared to SUN2, our data suggest that it is not redundant. A key finding shows that the interaction between SUN2 and lamin A/C is significantly weakened upon SUN1 KO. This indicates that SUN1 is required to stabilize the SUN–lamina interaction complex. Together, these findings identify a SUN1/2–lamin A–LAD axis as a nuclear mechanical feedback pathway tuning adhesion gene expression. SUN2 acts as the core effector of the feedback loop, while SUN1 serves as an essential stabilizer that facilitates the binding of SUN2 to the lamina.

SUN1/2 deficiency led to a dissociation and fragmentation of peripheral heterochromatin, evidenced by altered H3K9me3 foci number/size and the inward redistribution of H3K9me2. This morphological disintegration was accompanied by a transcriptional shift in chromatin-modifying enzymes. This structural failure, initiated by lamin A/C mislocalization, precipitates the malpositioning of chromatin, including the aberrant “gain” of genomic regions into LADs. Concurrently, these anomalous regions undergo epigenetic reprogramming to adapt to their repressive nuclear localization, as exemplified by the gain of H3K9me2 and loss of H3K4me1 at loci like *Rhoa* and *Cdc42* in *Lmna* KO cells. This combined spatial and epigenetic remodeling results in the stable silencing of genes like *Rhoa* and *Cdc42*. Therefore, we propose that SUN1/2 act as key organizers of the nuclear mechanical landscape. Their loss initiates a cascade: structural collapse (lamin A/C mislocalization) → spatial chromatin mis-organization (LAD gain and heterochromatin fragmentation) and epigenetic adaptation (H3K9me2/3 redistribution and modifier expression changes) → gene silencing.

In summary, SUN1/2 are critical regulators of nuclear mechanotransduction and cellular mechanical adaptation. By maintaining nuclear–cytoskeletal connectivity, SUN1/2 preserve LAD organization and adhesion gene expression, enabling dynamic mechanosensitivity adjustment to extracellular forces. The SUN1/2-dependent mechano-feedback loop highlights how the nucleus itself calibrates cellular responses to mechanical stress, preventing maladaptive outcomes such as adhesion loss and impaired mechanosensitivity. These insights not only expand our understanding of nuclear mechanics but also provide a framework to investigate how dysregulated nuclear feedback contributes to pathological states, including laminopathies and muscle disorders. Targeting SUN1/2-mediated nuclear feedback may represent a promising strategy for restoring mechanical resilience in disease contexts.

## Materials and Methods

### C2C12 KO cell lines and culture

C2C12 were grown in Dulbecco’s Modified Eagle’s Medium (DMEM) (Gibco) supplemented with 10% fetal bovine serum (Sigma) and 1% penicillin–streptomycin (Gibco). For *Sun1*, *Sun2*, and *Lmna* KO cell lines, 2 single guide RNAs (sgRNAs) targeting gene exons (*Sun1*: GTCCCGCCGCAGCTTGCGTCTGG and TAATGTGCGAATCAATAGCCTGG; *Sun2*: CACAGACTCGCTGTAGTAGGAGG and GGACAGGTGCTTCATGTTGCTGG; *Lmna*: GCGAGCTCCATGACCTGCGG and TCTCAGTGAGAAGCGCACAT) were designed using CCTop Web (https://crispr.cos.uni-heidelberg.de/) and cloned into the pX458-Cas9-GFP (green fluorescent protein) vector (Addgene #48138). C2C12 cells were transfected with the sgRNA plasmids using Lipofectamine 3000 (Thermo Scientific). GFP-positive cells were sorted by Fluorescence-Activated Cell Sorting (FACS) at 48 h posttransfection and single cells were expanded clonally. KO was verified by Sanger sequencing, Western blot, and immunofluorescence.

MSCs were grown in α-MEM supplemented with 10% fetal bovine serum and 1% penicillin–streptomycin.

### siRNA transfection

MSCs were transfected at 70% confluency using Lipofectamine RNAiMAX Reagent (Invitrogen) as per the manufacturer’s protocol (Publication No. MAN0007825 Rev.1.0) with the siRNAs. The siRNA targeting sequences used were as follows: mouse *Sun1*: Forward 5′-GGCUAUUGAUUCGCACAUUTT-3′, Reverse 5′-AAUGUGCGAAUCAAUAGCCTT-3′; mouse *Sun2*: Forward 5′-CUCUCAGGAUGAUAACGAUTT-3′, Reverse 5′-AUCGUUAUCAUCCUGAGAGTT-3′.

### Construction of plasmids for SUN1, SUN2, and the SUN2 Δ1–200 mutant

The coding sequences of SUN1 and SUN2 were synthesized by Genewiz. An N-terminally truncated SUN2 mutant lacking amino acids 1 to 200 (SUN2 Δ1–200) was amplified using the synthesized SUN2 as a template, while mCherry was amplified from a previously constructed mCherry-containing plasmid. Full-length SUN1, full-length SUN2, and the N-terminally truncated SUN2 mutant (SUN2 Δ1–200) were each cloned together with mCherry into the pSin vector using the ClonExpress Ultra One Step Cloning Kit (Vazyme C115-01). All final constructs were verified by Sanger sequencing performed by Genewiz.

### Microfluidic device fabrication

To fabricate the microfluidic structures, a 2-step replica molding process was employed using a custom-produced silicon master with defined micropatterns (BYMICROFAB Corporation). The master served as a template for generating polydimethylsiloxane (PDMS, Sylgard 184, Dow Corning) replicas, as schematically depicted in Fig. S3. Briefly, a base-to-curing agent mixture of PDMS at a 10:1 weight ratio was degassed for 30 min under vacuum, cast onto the silicon master, and cured at 60 °C for 3 h to form a negative replica. After peeling, this replica was exposed to oxygen plasma and subsequently silanized overnight with 3-(triethoxysilyl)-1-propanamine under vacuum conditions. To obtain the final positive PDMS structure, the same PDMS mixture was poured onto the silanized negative template, degassed, and cured overnight at 60 °C, followed by careful detachment. For device assembly, both the positive PDMS replica and a glass coverslip were treated with oxygen plasma (45 W, 60 s) to render their surfaces hydrophilic and enable irreversible bonding upon conformal contact. The assembled devices were then baked at 60 °C for 2 h to enhance adhesion. This bonding protocol was consistently applied, including for devices initially released from silicon substrates.

### Preparation of polyacrylamide gels

Polyacrylamide (PAA) gels were prepared as described previously [63]. In brief, gels with tunable stiffness (0.2 and 10 kPa) were obtained by adjusting the acrylamide–to–bis-acrylamide ratio (Sangon Biotech A100486). Polymerization was initiated by adding 10% ammonium persulfate (APS) and catalyzed with *N*,*N*,*N*′,*N*′-tetramethylethylenediamine (Sangon Biotech A610508). The precursor solution was placed between 2 glass slides and allowed to polymerize completely under 365-nm ultraviolet light for 30 min. Subsequently, the gels were coated with 20 μg/ml fibronectin overnight at 4 °C. After washing with PBST, cells were seeded on the gels. Experiments were conducted between 1 and 6 days postseeding.

### Myogenic differentiation

Cells were cultured upon reaching 90% confluency and induced to differentiate by the addition of DMEM with 2% horse serum (Gibco #26050088). Differentiation media was replaced every 24 h for the first 72 h, and subsequently every 12 h thereafter. After 6 days of differentiation, immunofluorescence staining was performed to evaluate myosin expression and localization.

### CalyA treatment

Cells were cultured for 12 h and CalyA (1 nM in dimethyl sulfoxide, Cell Signaling 9902) was added for 20 min. Immunofluorescence staining was performed to evaluate Vinculin density.

### Immunofluorescence

Cells were fixed with 4% paraformaldehyde for 15 min at room temperature, permeabilized with 0.25% Triton X-100 for 30 min, and blocked in PBST containing 5% bovine serum albumin (BSA) for 2 h. Cells were then incubated with primary antibodies diluted in PBST containing 1% BSA overnight at 4 °C, followed by washing 3 times with PBST. Cells were then incubated with secondary antibodies for 1 h at room temperature, followed by washing 3 times with PBST. Samples were incubated with Hoechst 33342 (1:1,000, Cell Signaling 4082S) for 10 min to stain the nucleus. After washing twice with PBST, confocal imaging was performed in a Dragonfly Confocal Microscopy System (Seven-laser).

Primary antibodies included the following: TRITC Phalloidin (1:200, Biosharp BL1189A), H3K9me3 (1:500, Abcam ab8898), H3K9me2 (1:500, Active Motif 39239), H3K4me1 (1:500, Abcam ab8895), Lamin B1 (1:500, Santa Cruz sc-374015), Lamin A/C (1:500, Cell Signaling 4777), Myosin 4 (1:100, eBioscience 14-6503-82), and Vinculin (1:200, Sigma-Aldrich V9264). Secondary antibodies included the following: anti-Rabbit Alexa 488 (1:2,000, Cell Signaling 4412S), anti-Mouse Alexa 594 (1:2,000, Cell Signaling 8890S), and anti-Mouse Alexa 488 (1:2,000, Cell Signaling 4408S).

### Nanobody-assisted nanoluciferase fragment complementation for in situ measurement and visualization of endogenous protein–protein interaction

A 96-well plate was coated with fibronectin and incubated at 37 °C for 4 h. Cells were seeded at 20,000 cells per well and cultured for 12 h. Cells were fixed with 4% PFA for 30 min. Permeabilization was carried out using 0.25% Triton X-100 for 30 min. Cells were then blocked with 5% BSA in PBST for 2 h. Primary antibody diluted in 1% BSA was added and incubated overnight at 4 °C. Then, cells were washed with PBST 3 times and incubated with secondary antibody (50 nM in 1% BSA with 1 mM DTT) for 1 h. Cells were washed with PBST and stained with 0.1% propidium iodide (PI) for 10 min. After washing twice with PBST, the PI intensity was read by a microplate reader (Ex/Em = 535/617 nm, BioTek Synergy H1). Prediluted substrates (Nano-Glo Live Cell Assay System) were prepared in advance and added per well for luminescence detection (BioTek Synergy H1).

Primary antibodies included the following: Lamin A/C (1:500, Active Motif 39287), SUN1 (1:200, Abcam ab10302), and SUN2 (1:300, Abcam ab124916). Secondary antibodies included the following: anti-Rabbit SB128-GFP-IgG + anti-Mouse LG-mCherry-IgG2a [54].

### Western blot

Protein extraction was performed using RIPA lysis buffer. Then, the samples were separated by 10% SDS-PAGE and transferred to an ImmobilonFL PVDF membrane (Millipore #IPVH00010). Membranes were blocked with 5% nonfat milk in PBST for 2 h at room temperature and incubated with primary antibodies (Integrin alpha 4, 1:1,000, Cell Signaling 8440S; Lamin A/C, 1:1,000, Cell Signaling 4777; GAPDH, 1:5,000, Cell Signaling 2118) overnight at 4 °C. After washing with PBST, membranes were incubated with corresponding horseradish peroxidase-conjugated secondary antibodies (anti-Mouse, 1:5,000, Cell Signaling 7076; anti-Rabbit, 1:10,000, Cell Signaling 7074) for 1 h at room temperature. Finally, the signals were visualized using the Omni-ECL Femto Light Chemiluminescence Kit (Epizyme) and a ChemiDoc MP Imaging System (Bio-Rad). ImageJ software was utilized to analyze Western blot data.

### Quantitative real-time PCR

Total RNA was extracted with the Total RNA Kit I (Omega R6834) in accordance with the manufacturer’s protocol. Subsequently, 500 ng of RNA was reverse-transcribed into cDNA per reaction using random primers and Reverse Transcriptase (Takara RR036A). PCR was conducted on an MX3000P Stratagene system with Hieff qPCR SYBR Green Master Mix (Yeasen 11204ES08) and gene-specific primers. Gene expression levels were normalized to GAPDH as an endogenous control. The primer sequences used were as follows: GAPDH: Forward 5′-CAGAAGACTGTGGATGGCCC-3′, Reverse 5′-ATCCACGACGGACACATTGG-3′; *Itga4*: Forward 5′-GGTCCCAGGCTACATCGTTTT-3′, Reverse 5′-GGGGTAAGGATGTCTCGCAC-3′; *Zyx*: Forward 5′-TGTTACAAGTGTGAGGACTGTGG-3′, Reverse 5′-CTCTAGACTCACGGCTTCCTT-3′; *Rhoa*: Forward 5′-CCGTATTATTGGAGATTGTCCTTG-3′, Reverse 5′-CTCTGGGAACTGGTCCTTGC-3′; *Cdc42*: Forward 5′-GGCGGAGAAGTGAGGAC-3′, Reverse 5′-TGCATAGTTGTCAAAAACAGTTGGT3′; *Sun1*: Forward 5′-GCAGGAGATGGGCACCATAG-3′, Reverse 5′-CTGCTCTTCATCCCT-GCTGC-3′; *Sun2*: Forward 5′-TCCAGGTTAAGGAGTGCAGC-3′, Reverse 5′-AAGTGCCTGGACCTGGTTAG-3′.

### RNA-seq and analysis

Total RNA was isolated from C2C12, *Sun1* KO, *Sun2* KO, and *Lmna* KO cells using the Total RNA Kit I and processed for Illumina library preparation. The mRNA samples were sequenced on DNBSEQ-T7 platform with 150 bp paired-end. Raw reads were first preprocessed using Ktrim v1.3.0 to remove sequencing adapters and low-quality reads. Then, clean reads were aligned to mm10 reference genome using STAR v2.7.3a. Transcript abundance was estimated using featureCounts tools from Subread v2.0.1 against ENSEMBL gene annotation v102. Differential expression gene (FDR < 0.05 and fold change ≥ 1.2) was determined using DESeq2 v1.30.0. Functional annotation was performed using the DAVID website.

Data of THP-1, HUVEC, CT26, and adipogenic cells were downloaded from the Gene Expression Omnibus (GSE85022, GSE213099, GSE250602, and GSE193505).

### Cleavage under targets and tagmentation

*Sun1* KO, *Sun2* KO, or *Lmna* KO C2C12 cells were subjected to CUT&Tag assay as previously described [64] by Hieff NGS G-Type In-Situ DNA Binding Profiling Library Prep Kit for Illumina (Yeasen 12598ES12) according to the manufacturer’s instructions. In brief, 100,000 cells were harvested and washed twice, then incubated with activated Concanavalin A-coated magnetic beads. Primary antibody (Lamin B1, Abcam ab10648; H3K9me2, Active Motif 39239; H3K4me1, Abcam ab8895) diluted 1:50 in blocking buffer was added and incubated on a rotating platform at 4 °C overnight. Then, Guinea Pig anti-Rabbit secondary antibody (ABIN101961) diluted 1:50 in blocking buffer was added and incubated for 1 h. After washing, cells were segmented with pA/G-Tn5 transposase at 37 °C for 1 h. After 30-min incubation with proteinase K at 55 °C, DNA was extracted and the libraries were prepared through PCR. Libraries were quantified, quality assessed, and sequenced by Qubit 4.0, Agilent 4200 Bioanalyzer and Illumina NovaSeq X platform.

### CUT&Tag analysis

Raw reads were aligned to mm10 genome using Bowtie2 v0.7.17 and then converted to sam format. Sam files were then filtered using samtools v1.7, sorted, and converted to bam format. PCR duplicates were removed using the Mark-Duplicates program in Picard tools. Replicate bigwig coverage tracks were made using Deeptools v3.1.1 bamCoverage. Log2-transformed tracks are shown using a genome browser (Integrative Genomic Viewer). The top 1% peaks were called using SEACR with default settings. Samtools merge was used to combine biological replicate bam files. LAD, KDD, and KMD were called using EDD v1.1.18 with paired merged CUT&Tag and IgG bam files. LAD/KDD/KMD PCA was performed using the princomp function in R’s stats package. Resulting peak files, along with paired replicate bam files, were input into DiffBind for differential binding analysis using FDR < 0.1 as significance threshold and annotated using ChIPseeker v1.36.0.

### Statistical analysis

For image processing, images were analyzed using Imaris v9.9. Fluorescence intensity was measured using Image-Pro Plus, NIS-Elements, or ImageJ software. Data are presented as mean ± SD and were calculated using GraphPad Prism v9.5. Between-group differences were assessed using unpaired 2-tailed Student *t* tests or one-way analysis of variance, and the post-hoc test was applied for multiple comparisons, such as Tukey’s HSD test, with a *P* value < 0.05 considered statistically significant. A contingency table was constructed comparing the frequency of LAD gain between down-regulated genes and nondifferentially expressed genes. Statistical significance was evaluated using Fisher’s exact test, and enrichment was quantified using odds ratios. Statistical methods are indicated in the figure legends.

## Data Availability

The data that support the findings of this study are available from the corresponding authors upon reasonable request.

## References

[1] Lv J, Liu X, Zhou Y, Cheng F, Chen H, Li S, Wang D, Zhou L, Wang Z, Zhou N, Chen J, Huang B. YAP inactivation by soft mechanotransduction relieves MAFG for tumor cell dedifferentiation. *Research*. 2023;6. 10.34133/research.0215 PMC10443527 37614365

[2] Wu M, Yang H, Liu S, Jiang L, Liang T, Wang Y, Zhu M, Song X, Liu H, Shen J, et al. Enhanced engraftment of human haematopoietic stem cells via mechanical remodelling mediated by the corticotropin-releasing hormone. Nat Biomed Eng. 2025;9(5):754–771. 39715892 10.1038/s41551-024-01316-1

[3] Alisafaei F, Shakiba D, Hong Y, Ramahdita G, Huang Y, Iannucci LE, Davidson MD, Jafari M, Qian J, Qu C, et al. Tension anisotropy drives fibroblast phenotypic transition by self-reinforcing cell-extracellular matrix mechanical feedback. Nat Mater. 2025;24(6):955–965. 40128624 10.1038/s41563-025-02162-5 PMC12368865

[4] Nava MM, Miroshnikova YA, Biggs LC, Whitefield DB, Metge F, Boucas J, Vihinen H, Jokitalo E, Li X, García Arcos JM, et al. Heterochromatin-driven nuclear softening protects the genome against mechanical stress-induced damage. Cell. 2020;181(4):800–817.e22. 32302590 10.1016/j.cell.2020.03.052 PMC7237863

[5] Ouyang M, Hu Y, Chen W, Li H, Ji Y, Qiu L, Zhu L, Ji B, Bu B, Deng L. Cell mechanics regulates the dynamic anisotropic remodeling of fibril matrix at large scale. *Research*. 2023;6. 10.34133/research.0270 PMC11776286 39882542

[6] Echarri A. A multisensory network drives nuclear Mechanoadaptation. Biomolecules. 2022;12(3): Article 404. 35327596 10.3390/biom12030404 PMC8945967

[7] Park JE, Jo J, Xu K, Lee SA, Han SB, Lee YJ, Cho WK, Li B, Kim SH, Kim DH. Attenuated nuclear tension regulates progerin-induced mechanosensitive nuclear wrinkling and chromatin remodeling. Adv Sci. 2025;12(31): Article e2502375. 10.1002/advs.202502375 PMC12376529 40344643

[8] Krshnan L, Siu WS, van de Weijer M, Hayward D, Guerrero EN, Gruneberg U, Carvalho P. Regulated degradation of the inner nuclear membrane protein SUN2 maintains nuclear envelope architecture and function. eLife. 2022;11: Article e81573. 36318477 10.7554/eLife.81573 PMC9662817

[9] Goelzer M, Howard S, Zavala AG, Conway D, Rubin J, van Wijnen AJ, Uzer G. Depletion of SUN1/2 induces heterochromatin accrual in mesenchymal stem cells during adipogenesis. Commun Biol. 2025;8(1):428. 40082539 10.1038/s42003-025-07832-3 PMC11906923

[10] Li T, Qiu J, Jia T, Liang Y, Zhang K, Yan W, Hou Z, Yang S, Liu L, Xiong W, et al. G3BP2 regulates oscillatory shear stress-induced endothelial dysfunction. Genes Dis. 2022;9(6):1701–1715. 36157502 10.1016/j.gendis.2021.11.003 PMC9485288

[11] Li T, Safitri M, Zhang K, Wang Y, Huang L, Zhu Y, Daniel R, Wu LJ, Qiu J, Wang G. Downregulation of G3BP2 reduces atherosclerotic lesions in ApoE(-/-) mice. Atherosclerosis. 2020;310:64–74. 32919187 10.1016/j.atherosclerosis.2020.08.003

[12] Wei F, Xu X, Zhang C, Liao Y, Ji B, Wang N. Stress fiber anisotropy contributes to force-mode dependent chromatin stretching and gene upregulation in living cells. Nat Commun. 2020;11(1):4902. 32994402 10.1038/s41467-020-18584-5 PMC7524734

[13] Hoffman LM, Smith MA, Jensen CC, Yoshigi M, Blankman E, Ullman KS, Beckerle MC. Mechanical stress triggers nuclear remodeling and the formation of transmembrane actin nuclear lines with associated nuclear pore complexes. Mol Biol Cell. 2020;31(16):1774–1787. 31967947 10.1091/mbc.E19-01-0027 PMC7521858

[14] Mu X, Tseng C, Hambright WS, Matre P, Lin CY, Chanda P, Chen W, Gu J, Ravuri S, Cui Y, et al. Cytoskeleton stiffness regulates cellular senescence and innate immune response in Hutchinson-Gilford progeria syndrome. Aging Cell. 2020;19(8): Article e13152. 32710480 10.1111/acel.13152 PMC7431831

[15] Lu C, Huang G, Zuo Z, Xu F, Zhao C, Wang G, Peng Q, Qiu J. A comprehensive review of nuclear mechanics: Advances, disease relevance, methodologies, and AI applications. Cell Biochem Biophys. 2025;84:525–547. 41342991 10.1007/s12013-025-01964-3

[16] Harada T, Swift J, Irianto J, Shin JW, Spinler KR, Athirasala A, Diegmiller R, Dingal PCDP, Ivanovska IL, Discher DE. Nuclear lamin stiffness is a barrier to 3D migration, but softness can limit survival. J Cell Biol. 2014;204(5):669–682. 24567359 10.1083/jcb.201308029 PMC3941057

[17] Janota CS, Calero-Cuenca FJ, Gomes ER. The role of the cell nucleus in mechanotransduction. Curr Opin Cell Biol. 2020;63:204–211. 32361559 10.1016/j.ceb.2020.03.001

[18] Walker CJ, Crocini C, Ramirez D, Killaars AR, Grim JC, Aguado BA, Clark K, Allen MA, Dowell RD, Leinwand LA, et al. Nuclear mechanosensing drives chromatin remodelling in persistently activated fibroblasts. Nat Biomed Eng. 2021;5(12):1485–1499. 33875841 10.1038/s41551-021-00709-w PMC9102466

[19] Dang Y, Lattner J, Lahola-Chomiak AA, Afonso DA, Ulbricht E, Taubenberger A, Rulands S, Tabler JM. Self-propagating wave drives morphogenesis of skull bones in vivo. Nat Commun. 2025;16(1):4330. 40346043 10.1038/s41467-025-59164-9 PMC12064835

[20] Hallou A, He R, Simons BD, Dumitrascu B. A computational pipeline for spatial mechano-transcriptomics. Nat Methods. 2025;22(4):737–750. 40097810 10.1038/s41592-025-02618-1 PMC11978512

[21] Li Mow Chee F, Beernaert B, Griffith BGC, Loftus AEP, Kumar Y, Wills JC, Lee M, Valli J, Wheeler AP, Armstrong JD, et al. Mena regulates nesprin-2 to control actin-nuclear lamina associations, trans-nuclear membrane signalling and gene expression. Nat Commun. 2023;14(1):1602. 36959177 10.1038/s41467-023-37021-x PMC10036544

[22] Bougaran P, Bautch VL. Life at the crossroads: The nuclear LINC complex and vascular mechanotransduction. Front Physiol. 2024;15:1411995. 38831796 10.3389/fphys.2024.1411995 PMC11144885

[23] Dupont S, Wickström SA. Mechanical regulation of chromatin and transcription. Nat Rev Genet. 2022;23(10):624–643. 35606569 10.1038/s41576-022-00493-6

[24] Kalukula Y, Stephens AD, Lammerding J, Gabriele S. Mechanics and functional consequences of nuclear deformations. Nat Rev Mol Cell Biol. 2022;23(9):583–602. 35513718 10.1038/s41580-022-00480-z PMC9902167

[25] Yue X, Cui J, Sun Z, Liu L, Li Y, Shao L, Feng Q, Wang Z, Hambright WS, Cui Y, et al. Nuclear softening mediated by Sun2 suppression delays mechanical stress-induced cellular senescence. Cell Death Discov. 2023;9(1):167. 37198162 10.1038/s41420-023-01467-1 PMC10192198

[26] Ueda N, Maekawa M, Matsui TS, Deguchi S, Takata T, Katahira J, Higashiyama S, Hieda M. Inner nuclear membrane protein, SUN1, is required for cytoskeletal force generation and focal adhesion maturation. Front Cell Dev Biol. 2022;10: Article 885859. 35663386 10.3389/fcell.2022.885859 PMC9157646

[27] Shiraishi K, Shah PP, Morley MP, Loebel C, Santini GT, Katzen J, Basil MC, Lin SM, Planer JD, Cantu E, et al. Biophysical forces mediated by respiration maintain lung alveolar epithelial cell fate. Cell. 2023;186(7):1478–92.e15. 36870331 10.1016/j.cell.2023.02.010 PMC10065960

[28] Dhankhar M, Guo Z, Kant A, Basir R, Joshi R, Vinayak V, Heo SC, Mauck RL, Lakadamyali M, Shenoy VB. Revealing the biophysics of lamina-associated domain formation by integrating theoretical modeling and high-resolution imaging. Nat Commun. 2025;16(1):7909. 40854894 10.1038/s41467-025-63244-1 PMC12378204

[29] Heo SJ, Thakur S, Chen X, Loebel C, Xia B, McBeath R, Burdick JA, Shenoy VB, Mauck RL, Lakadamyali M. Aberrant chromatin reorganization in cells from diseased fibrous connective tissue in response to altered chemomechanical cues. Nat Biomed Eng. 2023;7(2):177–191. 35996026 10.1038/s41551-022-00910-5 PMC10053755

[30] Pal M, Schauer T, Burton A, Nakatani T, Pecori F, Hernández-Giménez A, Nadelson I, Marti-Renom MA, Torres-Padilla ME. The establishment of nuclear organization in mouse embryos is orchestrated by multiple epigenetic pathways. Cell. 2025;188(13):3583–3602.e21. 40273908 10.1016/j.cell.2025.03.044

[31] Wang Y, Elsherbiny A, Kessler L, Cordero J, Shi H, Serke H, Lityagina O, Trogisch FA, Mohammadi MM, el-Battrawy I, et al. Lamin A/C-dependent chromatin architecture safeguards naïve pluripotency to prevent aberrant cardiovascular cell fate and function. Nat Commun. 2022;13(1):6663. 36333314 10.1038/s41467-022-34366-7 PMC9636150

[32] Shah PP, Lv W, Rhoades JH, Poleshko A, Abbey D, Caporizzo MA, Linares-Saldana R, Heffler JG, Sayed N, Thomas D, et al. Pathogenic LMNA variants disrupt cardiac lamina-chromatin interactions and de-repress alternative fate genes. Cell Stem Cell. 2021;28(5):938–954.e9. 33529599 10.1016/j.stem.2020.12.016 PMC8106635

[33] Sen B, Xie Z, Thomas MD, Pattenden SG, Howard S, McGrath C, Styner M, Uzer G, Furey TS, Rubin J. Nuclear actin structure regulates chromatin accessibility. Nat Commun. 2024;15(1):4095. 38750021 10.1038/s41467-024-48580-y PMC11096319

[34] Guo M, Wong IY, Moore AS, Medalia O, Lippincott-Schwartz J, Weitz DA, Goldman RD. Vimentin intermediate filaments as structural and mechanical coordinators of mesenchymal cells. Nat Cell Biol. 2025;27(8):1210–1218. 40764390 10.1038/s41556-025-01713-x

[35] Kirby TJ, Lammerding J. Emerging views of the nucleus as a cellular mechanosensor. Nat Cell Biol. 2018;20(4):373–381. 29467443 10.1038/s41556-018-0038-y PMC6440800

[36] Chastney MR, Kaivola J, Leppänen VM, Ivaska J. The role and regulation of integrins in cell migration and invasion. Nat Rev Mol Cell Biol. 2025;26(2):147–167. 39349749 10.1038/s41580-024-00777-1

[37] Wang H, Said R, Nguyen-Vigouroux C, Henriot V, Gebhardt P, Pernier J, Grosse R, le Clainche C. Talin and vinculin combine their activities to trigger actin assembly. Nat Commun. 2024;15(1):9497. 39489770 10.1038/s41467-024-53859-1 PMC11532549

[38] Schwartz MA. The force is with us. Science. 2009;323(5914):588–589. 19179515 10.1126/science.1169414

[39] Swift J, Ivanovska IL, Buxboim A, Harada T, Dingal PCDP, Pinter J, Pajerowski JD, Spinler KR, Shin JW, Tewari M, et al. Nuclear lamin-A scales with tissue stiffness and enhances matrix-directed differentiation. Science. 2013;341(6149):1240104. 23990565 10.1126/science.1240104 PMC3976548

[40] Jiao S, Li C, Guo F, Zhang J, Zhang H, Cao Z, Wang W, Bu W, Lin M, Lü J, et al. SUN1/2 controls macrophage polarization via modulating nuclear size and stiffness. Nat Commun. 2023;14(1):6416. 37828059 10.1038/s41467-023-42187-5 PMC10570371

[41] Buglak DB, Bougaran P, Kulikauskas MR, Liu Z, Monaghan-Benson E, Gold AL, Marvin AP, Burciu A, Tanke NT, Oatley M, et al. Nuclear SUN1 stabilizes endothelial cell junctions via microtubules to regulate blood vessel formation. eLife. 2023;12: Article e83652. 36989130 10.7554/eLife.83652 PMC10059686

[42] Nasr S, Li L, Asad M, Moridi M, Wang M, Zemp FJ, Mahoney DJ, Wang E. A computational pipeline for identifying gene targets and signalling pathways in cancer cells to improve lymphocyte infiltration and immune checkpoint therapy efficacy. EBioMedicine. 2024;104: Article 105167. 38805852 10.1016/j.ebiom.2024.105167 PMC11154126

[43] Ishihara H, Martin BL, Brautigan DL, Karaki H, Ozaki H, Kato Y, Fusetani N, Watabe S, Hashimoto K, Uemura D, et al. Calyculin A and okadaic acid: Inhibitors of protein phosphatase activity. Biochem Biophys Res Commun. 1989;159(3):871–877. 2539153 10.1016/0006-291x(89)92189-x

[44] Ito-Ishida A, Baker SA, Sillitoe RV, Sun Y, Zhou J, Ono Y, Iwakiri J, Yuzaki M, Zoghbi HY. MeCP2 levels regulate the 3D structure of heterochromatic foci in mouse neurons. J Neurosci. 2020;40(45):8746–8766. 33046553 10.1523/JNEUROSCI.1281-19.2020 PMC7643291

[45] Sun C, Zhao Y, Guo L, Qiu J, Peng Q. The interplay between histone modifications and nuclear lamina in genome regulation. J Genet Genomics. 2025;52(1):24–38. 39426590 10.1016/j.jgg.2024.10.005

[46] Madsen-Østerbye J, Abdelhalim M, Baudement MO, Collas P. Local euchromatin enrichment in lamina-associated domains anticipates their repositioning in the adipogenic lineage. Genome Biol. 2022;23(1):91. 35410387 10.1186/s13059-022-02662-6 PMC8996409

[47] Sun L, Xie Y, Zuo Z, Liu J, Yang J, Ali I, Peng Q, Qiu J. Decreasing lamin A triggers cell fate transitions through heterochromatin-nuclear periphery detethering. Biomater Res. 2025;29:0256. 40980288 10.34133/bmr.0256 PMC12444033

[48] Marin HC, Allen C, Simental E, Martin EW, Panning B, al-Sady B, Buchwalter A. The nuclear periphery confers repression on H3K9me2-marked genes and transposons to shape cell fate. Nat Cell Biol. 2025;27(8):1311–1326. 40696106 10.1038/s41556-025-01703-z PMC12339402

[49] Kubo N, Chen PB, Hu R, Ye Z, Sasaki H, Ren B. H3K4me1 facilitates promoter-enhancer interactions and gene activation during embryonic stem cell differentiation. Mol Cell. 2024;84(9):1742–1752.e5. 38513661 10.1016/j.molcel.2024.02.030 PMC11069443

[50] Benarroch L, Madsen-Østerbye J, Abdelhalim M, Mamchaoui K, Ohana J, Bigot A, Mouly V, Bonne G, Bertrand AT, Collas P. Cellular and genomic features of muscle differentiation from isogenic fibroblasts and myoblasts. Cells. 2023;12(15): Article 1995. 37566074 10.3390/cells12151995 PMC10417614

[51] Chal J, Pourquié O. Making muscle: Skeletal myogenesis in vivo and in vitro. Development. 2017;144(12):2104–2122. 28634270 10.1242/dev.151035

[52] Kjaer M. Role of extracellular matrix in adaptation of tendon and skeletal muscle to mechanical loading. Physiol Rev. 2004;84(2):649–698. 15044685 10.1152/physrev.00031.2003

[53] Van Steensel B, Belmont AS. Lamina-associated domains: Links with chromosome architecture, heterochromatin, and gene repression. Cell. 2017;169(5):780–791. 28525751 10.1016/j.cell.2017.04.022 PMC5532494

[54] Li Q, Liu H, Du X, Xie Y, Chen Y, Qiu J, Gao Y, Pen Q. Nanobody-assisted nanoluciferase fragment complementation for in situ measurement and visualization of endogenous protein-protein interaction. Biosens Bioelectron. 2025;272: Article 117102. 39752888 10.1016/j.bios.2024.117102

[55] Sun WW, Jiao S, Sun L, Zhou Z, Jin X, Wang JH. SUN2 modulates HIV-1 infection and latency through association with lamin A/C to maintain the repressive chromatin. MBio. 2018;9(3):e02408–e02417. 10.1128/mBio.02408-17 PMC5930302 29717016

[56] Nardone G, Oliver-de la Cruz J, Vrbsky J, Martini C, Pribyl J, Skládal P, Pešl M, Caluori G, Pagliari S, Martino F, et al. YAP regulates cell mechanics by controlling focal adhesion assembly. Nat Commun. 2017;8:15321. 28504269 10.1038/ncomms15321 PMC5440673

[57] Kovaleva A, Solomatina E, Tlegenova M, Saidova A, Vorobjev IA. How actin polymerization and myosin II activity regulate focal adhesion dynamics in motile cells. Int J Mol Sci. 2025;26(16): Article 7701. 40869022 10.3390/ijms26167701 PMC12386929

[58] Porter L, Minaisah RM, Ahmed S, Ali S, Norton R, Zhang Q, Ferraro E, Molenaar C, Holt M, Cox S, et al. SUN1/2 are essential for RhoA/ROCK-regulated actomyosin activity in isolated vascular smooth muscle cells. Cells. 2020;9(1): Article 132. 31935926 10.3390/cells9010132 PMC7017107

[59] Stricker J, Aratyn-Schaus Y, Oakes PW, Gardel ML. Spatiotemporal constraints on the force-dependent growth of focal adhesions. Biophys J. 2011;100(12):2883–2893. 21689521 10.1016/j.bpj.2011.05.023 PMC3123981

[60] Leopoldt D, HF Y Jr, Rozengurt E. Calyculin-A induces focal adhesion assembly and tyrosine phosphorylation of p125(Fak), p130(Cas), and paxillin in Swiss 3T3 cells. J Cell Physiol. 2001;188(1):106–119. 11382927 10.1002/jcp.1102

[61] Biedzinski S, Agsu G, Vianay B, Delord M, Blanchoin L, Larghero J, Faivre L, Théry M, Brunet S. Microtubules control nuclear shape and gene expression during early stages of hematopoietic differentiation. EMBO J. 2020;39(23): Article e103957. 33089509 10.15252/embj.2019103957 PMC7705455

[62] Briand N, Collas P. Lamina-associated domains: Peripheral matters and internal affairs. Genome Biol. 2020;21(1):85. 32241294 10.1186/s13059-020-02003-5 PMC7114793

[63] Tse JR, Engler AJ. Preparation of hydrogel substrates with tunable mechanical properties. Curr Protoc Cell Biol. 2010;Chapter 10: Article Unit 10.6. 10.1002/0471143030.cb1016s47 20521229

[64] Kaya-Okur HS, Wu SJ, Codomo CA, Pledger ES, Bryson TD, Henikoff JG, Ahmad K, Henikoff S. CUT&Tag for efficient epigenomic profiling of small samples and single cells. Nat Commun. 2019;10(1):1930. 31036827 10.1038/s41467-019-09982-5 PMC6488672

[65] Richards CJ, Wierenga ATJ, Brouwers-Vos AZ, Kyrloglou E, Dillingh LS, Mulder PPMFA, Palasantzas G, Schuringa JJ, Roos WH. Elastic properties of leukemic cells linked to maturation stage and integrin activation. iScience. 2025;28(4): Article 112150. 40201128 10.1016/j.isci.2025.112150 PMC11978321

[66] Luo Q, Kuang D, Zhang B, Song G. Cell stiffness determined by atomic force microscopy and its correlation with cell motility. Biochim Biophys Acta. 2016;1860(9):1953–1960. 27288584 10.1016/j.bbagen.2016.06.010

[67] Wang K, Qin Y, Chen Y. In situ AFM detection of the stiffness of the in situ exposed cell nucleus. Biochim Biophys Acta, Mol Cell Res. 2021;1868(5): Article 118985. 33600839 10.1016/j.bbamcr.2021.118985

