# Supplementary material for: A Mechano-Feedback Loop Orchestrated by SUN1/2 Governs Cellular Mechanoadaptation via Lamina-Associated Domain Remodeling
Source: Research (Wash D C). 2026 May 14;9:1259. doi: 10.34133/research.1259 (PMC13172581; doi:10.34133/research.1259)
Supplement: Supplementary 1 — Figs. S1 to S21 [file research.1259.f1.zip › Supplementary Materials.docx]

**SUPPLEMENTAL INFORMATION**


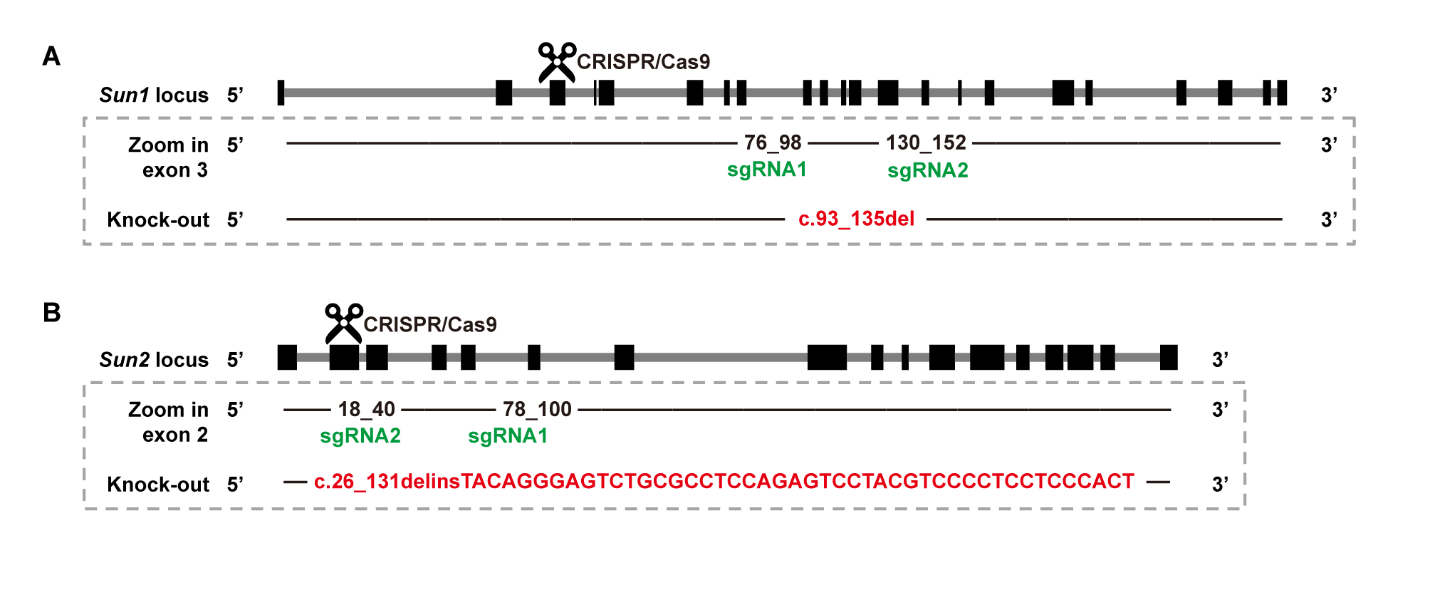


**Supplementary Fig. S1. Schematic of *Sun1* and *Sun2* genes knockout in C2C12.**

(A) Schematic diagram of *Sun1* gene knockout. Black blocks represent the exons of *Sun1*. Green indicates two sgRNA sequences targeting the exon 3 of *Sun1*. Red marks the deletion mutation sites.

(B) Schematic diagram of *Sun2* gene knockout. Black blocks represent the exons of *Sun2*. Green indicates two sgRNA sequences targeting the exon 2 of *Sun2*. Red marks the insertion and deletion mutation sites.


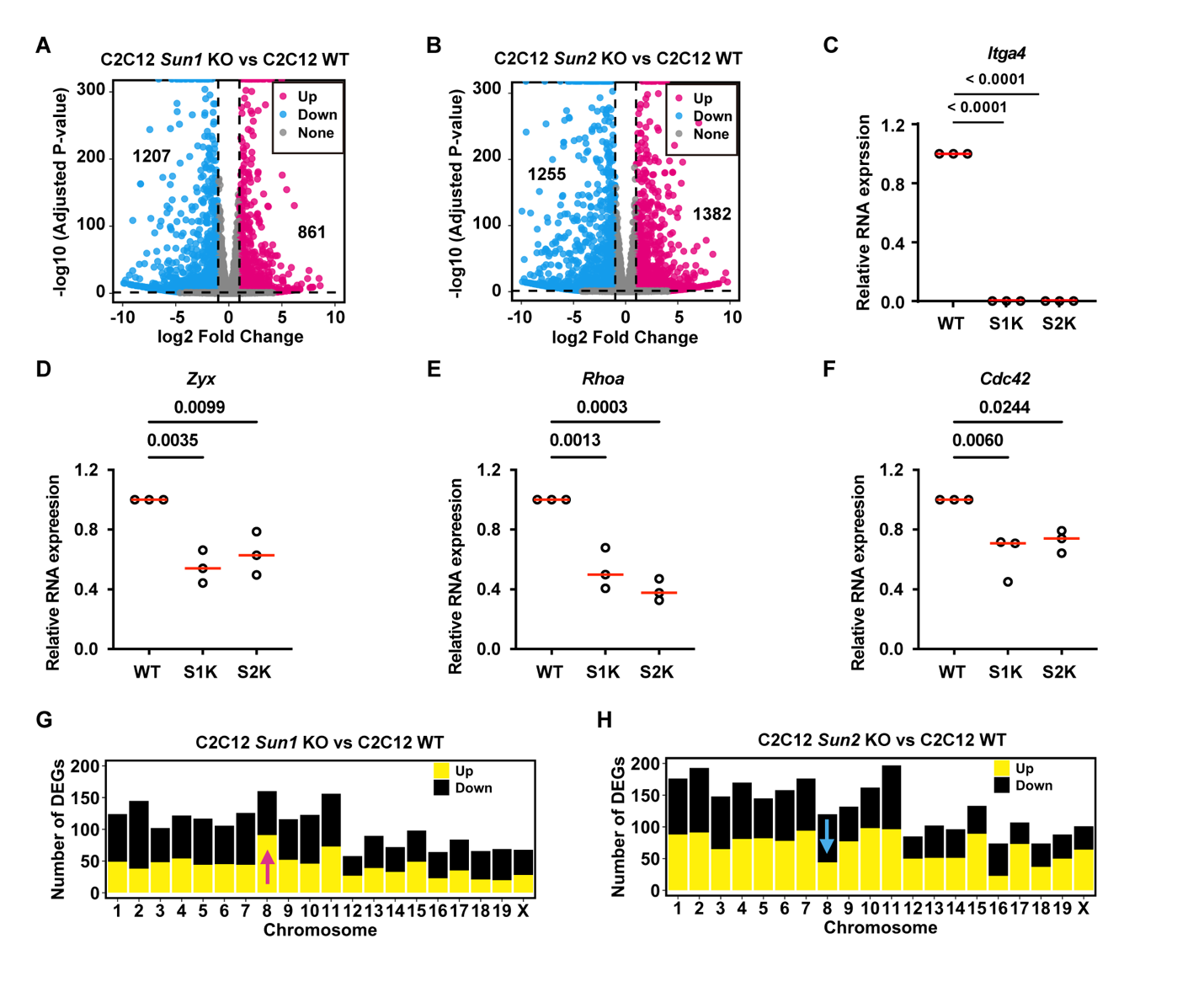


**Supplementary Fig. S2. Loss of SUN1 or SUN2 affects downstream gene transcription.**

(A and B) Volcano plot showing differentially expressed genes in the *Sun1* KO group compared to the C2C12 WT group (A) and in the *Sun2* KO group compared to the C2C12 WT group (B) with three replicates per group. Each dot represents a gene. The black dashed lines indicate the thresholds for filtering DEG: FDR < 0.05 and Fold Change ≥ 1.2. Red color indicates significantly upregulated genes, blue color indicates significantly downregulated genes, and gray indicates genes with no significant differences. The number of genes in each region is labeled accordingly.

(C-F) Quantification of *Itga4* (C), *Zyx* (D), *Rhoa* (E) and *Cdc42* (F) RNA transcription levels across the three cell groups. Each dot represents a biological replicate, and red line segments indicate the mean values. One-way ANOVA and Tukey's HSD test.

(G and H) The number of DEGs on each chromosome after *Sun1* (G) or *Sun2* (H) knockout.


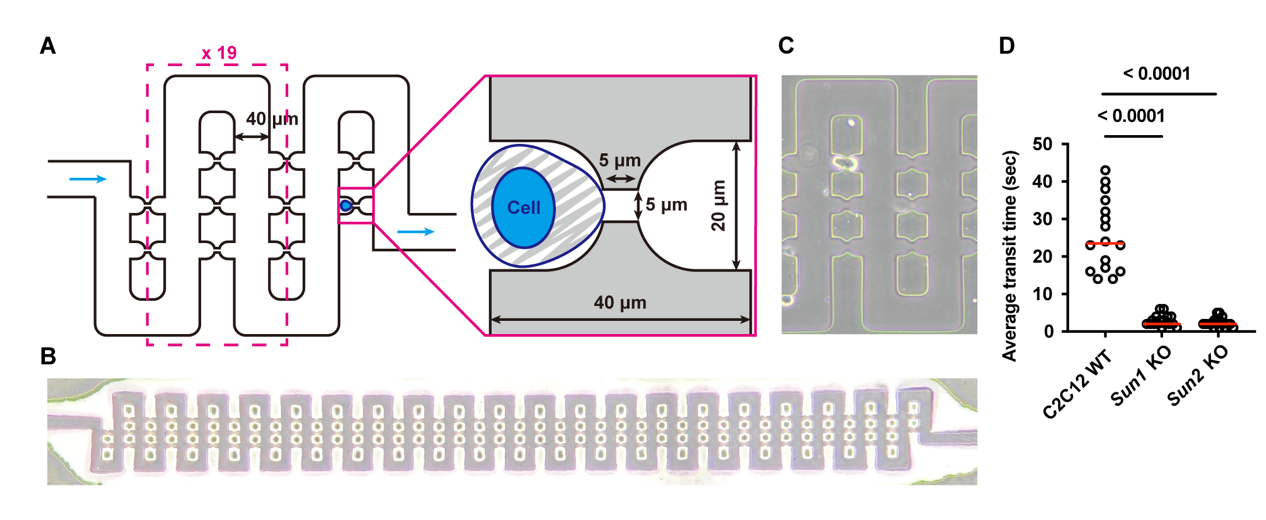


**Supplementary Fig. S3. The cellular deformability of C2C12 cells with *Sun1* KO or *Sun2* KO.**

(A) Schematic of the microfluidic device used to assess cell deformability. Cells flow through an array of constriction units (×19) with defined geometries. The zoom-in shows a single constriction channel with a 5 μm × 5 μm narrow passage connecting two wider chambers (20 μm width, 40 μm length), through which individual cells are forced to transit.

(B) Representative bright-field image of the microfluidic chip containing multiple parallel constriction units for high-throughput measurement.

(C) Representative time-lapse image of cells passing through the constriction channels.

(D) Quantification of average transit time to pass through the constriction for C2C12 WT, *Sun1* KO, and *Sun2* KO cells (n = 16, 17, 18). Each dot represents a single cell, and red lines indicate mean values. One-way ANOVA and Tukey's HSD test.


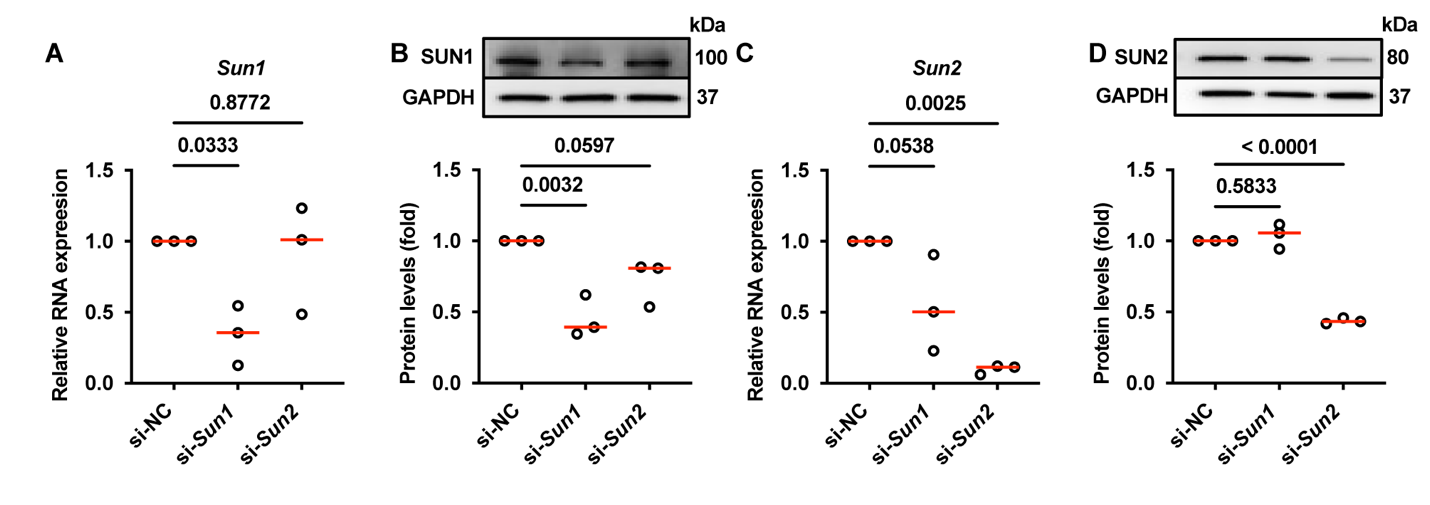


**Supplementary Fig. S4. The knockdown of *Sun1* or *Sun2* in mesenchymal stem cells.**

(A and C) Quantification of *Sun1* (A) and *Sun2* (C) RNA transcription levels across the three groups. Each dot represents a biological replicate, and red line segments indicate the mean values. One-way ANOVA and Tukey's HSD test. Si-NC represents the non-targeting control group.

(B and D) WB analysis of the SUN1 (B) and SUN2 (D) protein levels in *Sun1* or *Sun2* knockdown group (three biological replicates per group). Quantitative data to the bottom are presented as the mean with SD. One-way ANOVA and Tukey's HSD test. Si-NC represents the non-targeting control group.


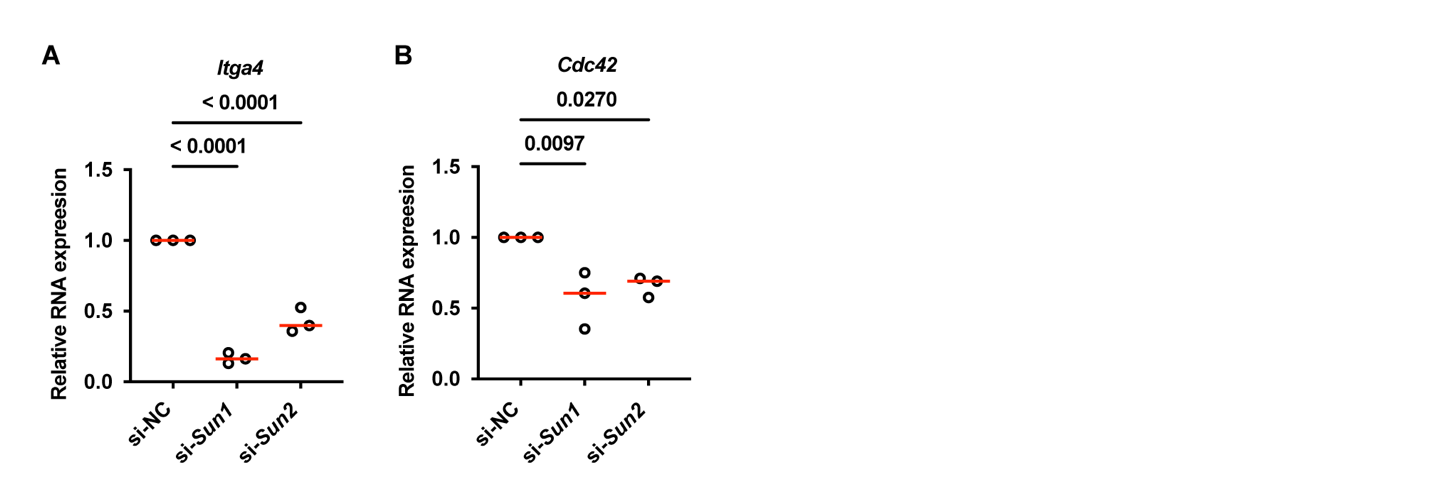


**Supplementary Fig. S5. *Sun1* or *Sun2* knockdown downregulates adhesion-related genes in mesenchymal stem cells.**

(A and B) Quantification of *Sun1* (A) and *Sun2* (B) RNA transcription levels across the three cell groups. Each dot represents a biological replicate, and red line segments indicate the mean values. One-way ANOVA and Tukey's HSD test. Si-NC represents the non-targeting control group.


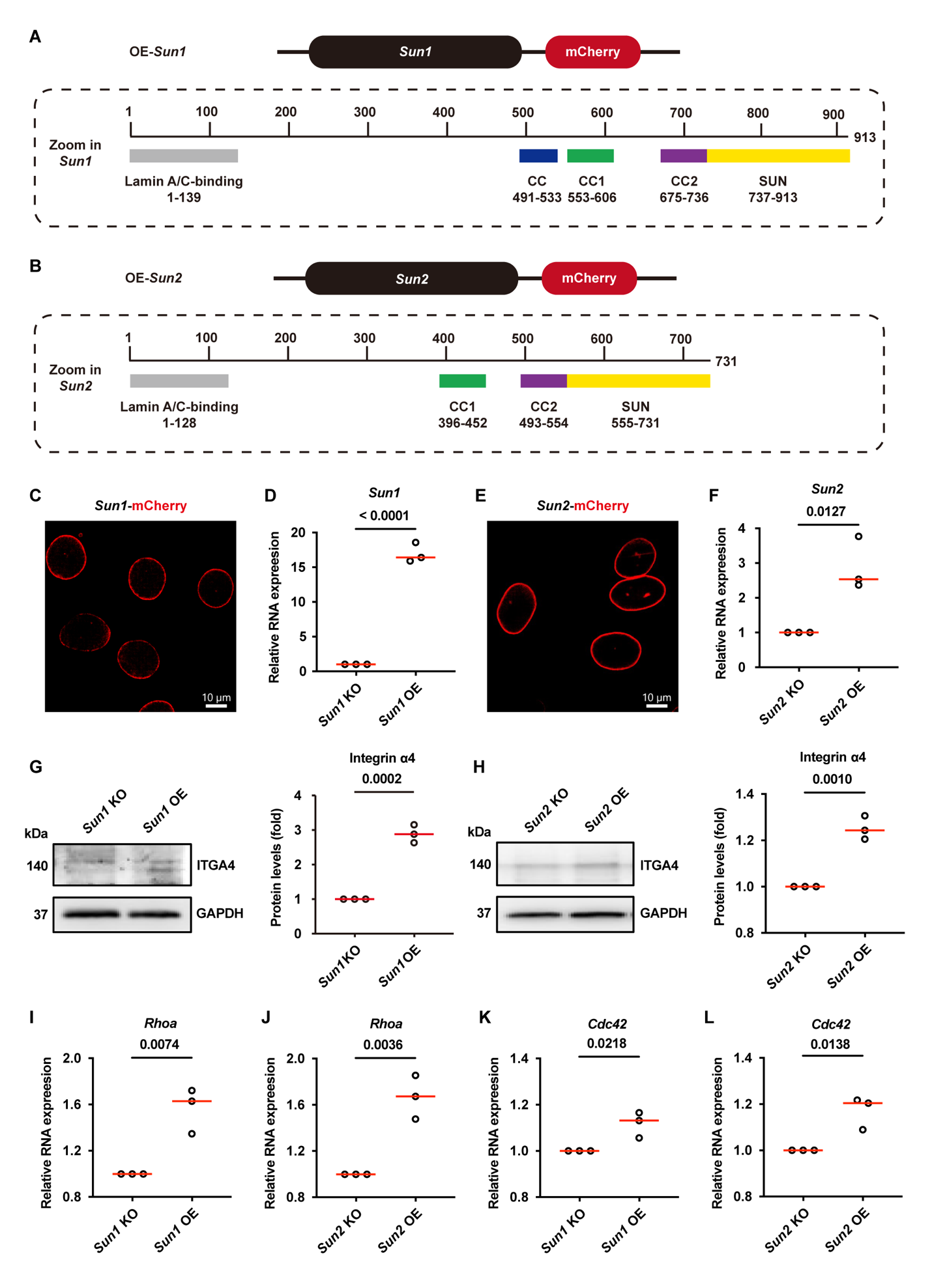


**Supplementary Fig. S6. Overexpression of SUN proteins rescue the downregulated expression of adhesion genes in *Sun*1/2 knockout cells.**

(A and B) The protein architecture and domain composition of *Sun1* (A) and *Sun2* (B) used for overexpression. A zoomed-in view annotates the key functional domains of each protein, including the lamin A/C-binding region, the coiled-coil (CC) domain, and the SUN domain, along with their corresponding amino acid residue numbers. mCherry was fused to the C-terminus of the proteins for live-cell imaging and subcellular localization analysis.

(C and E) Representative fluorescence images of *Sun1* KO (C) and *Sun2* KO (E) cells stably expressing mCherry-tagged *Sun1* (A) and *Sun2* (B) via viral infection. Scale bar, 10 μm.

(D, I and K) Quantification of *Sun1*, *Rhoa* and *Cdc42* RNA transcription levels in *Sun1* KO and *Sun1* OE cells. Each dot represents a biological replicate, and red line segments indicate the mean values. Two-tailed unpaired Student t-tests.

(F, J and L) Quantification of *Sun2*, *Rhoa* and *Cdc42* RNA transcription levels in *Sun2* KO and *Sun2* OE cells. Each dot represents a biological replicate, and red line segments indicate the mean values. Two-tailed unpaired Student t-tests.

(G and H) WB analysis of ITGA4 protein levels in *Sun1* OE and *Sun2* OE cells. (three biological replicates per group). Quantitative data to the right are presented as the mean with SD. Two-tailed unpaired Student t-tests.


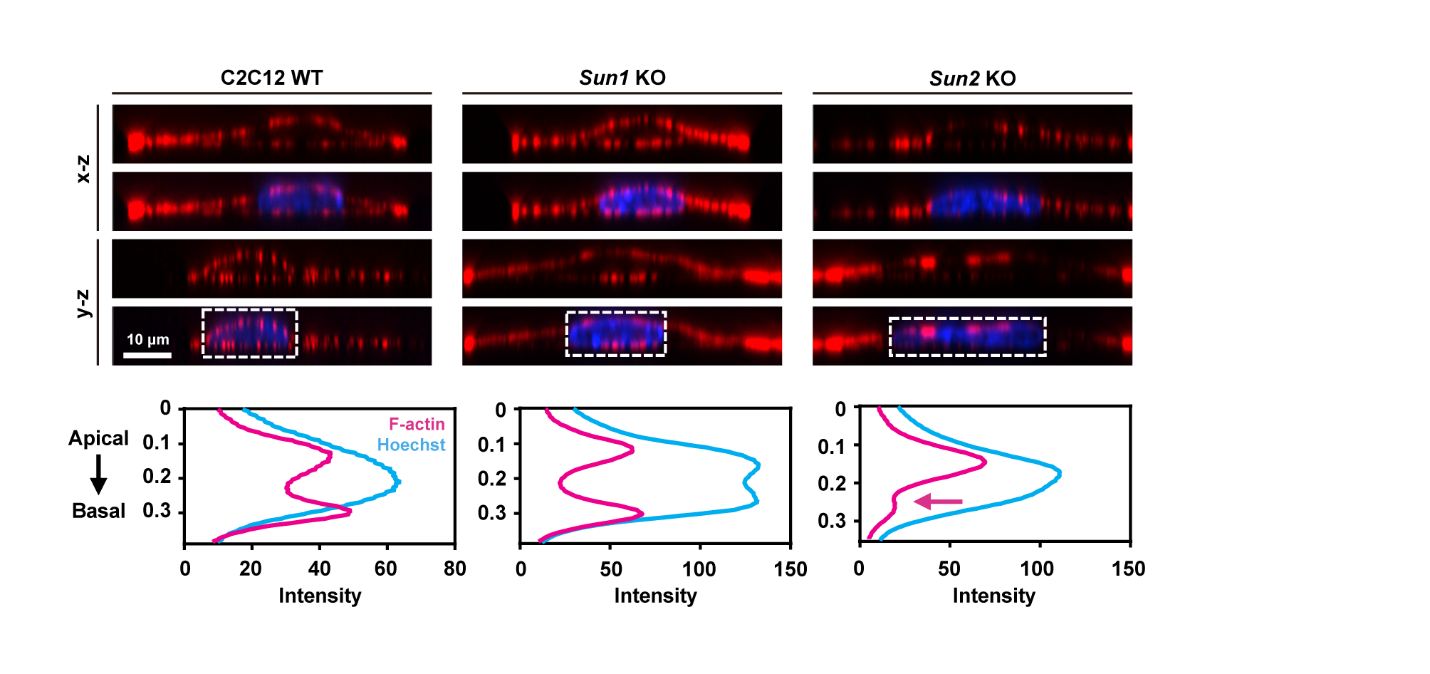


**Supplementary Fig. S7. Alterations in C2C12 cell cytoskeleton and nuclear morphology after knocking out *Sun1* or *Sun2*.**

Orthogonal views in the x-z and y-z planes, F-actin in red and nuclei in blue. Scale bar, 10 μm. The bottom graphs show quantitative fluorescence intensity profiles within the white boxed regions.


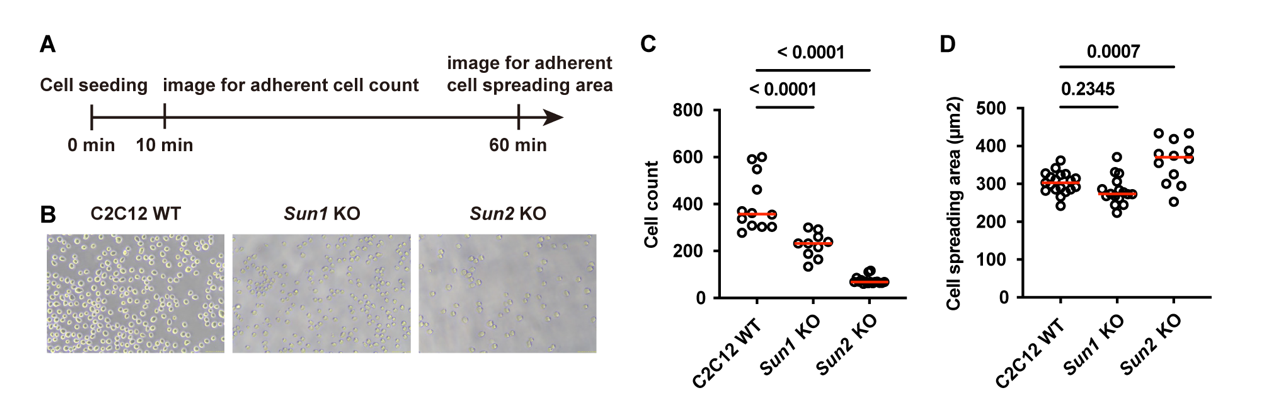


**Supplementary Fig. S8. The cellular adhesion and spreading of C2C12 cells with *Sun1* KO or *Sun2* KO.**

(A) Schematic of the experiment design and time point.

(B) Representative bright-field image of cell adhesion 10 min after planting.

(C) Quantification of cell adhesion for C2C12 WT, *Sun1* KO, and *Sun2* KO cells. Each dot represents the cell number from one image, and red lines indicate mean values. One-way ANOVA and Tukey's HSD test.

(D) Quantification of average cell spreading area for C2C12 WT, *Sun1* KO, and *Sun2* KO cells (n = 19, 15, 12). Each dot represents a single cell, and red lines indicate mean values. One-way ANOVA and Tukey's HSD test.


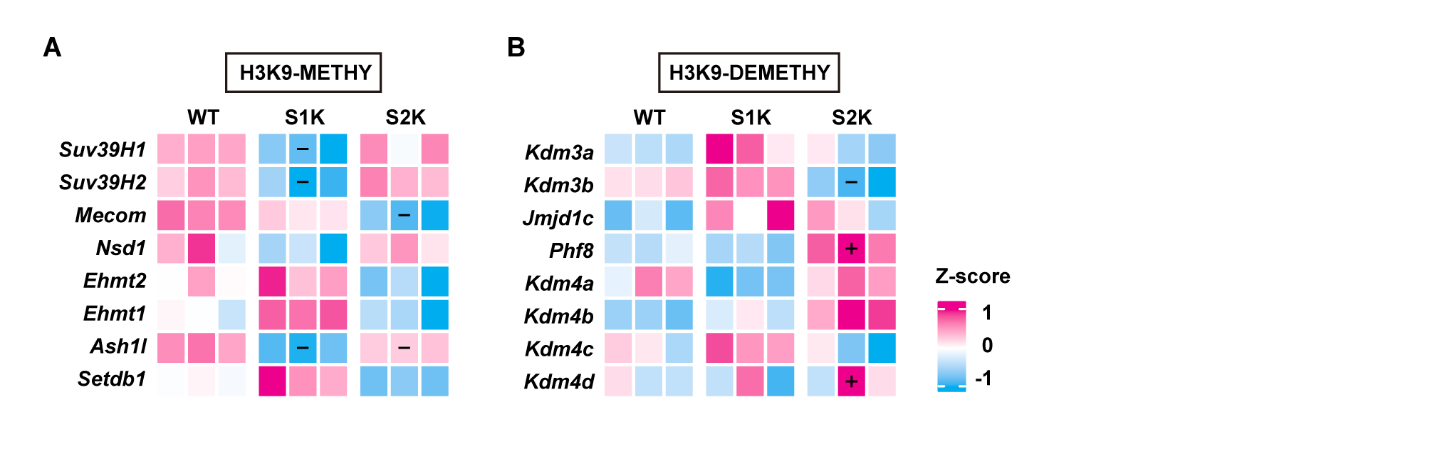


**Supplementary Fig. S9. The heterochromatic state also changes after knocking out *Sun1/2*.**

(A and B) The heatmap shows the expression change levels of H3K9 methylation-related (A) and H3K9 demethylation-related (B) genes in C2C12 WT, *Sun1* KO (S1K), and *Sun2* KO (S2K) cells (three replicates per group). Each row is standardized using the Z-score method. A “+” indicates significantly upregulated genes, and a “-” indicates significantly downregulated genes.


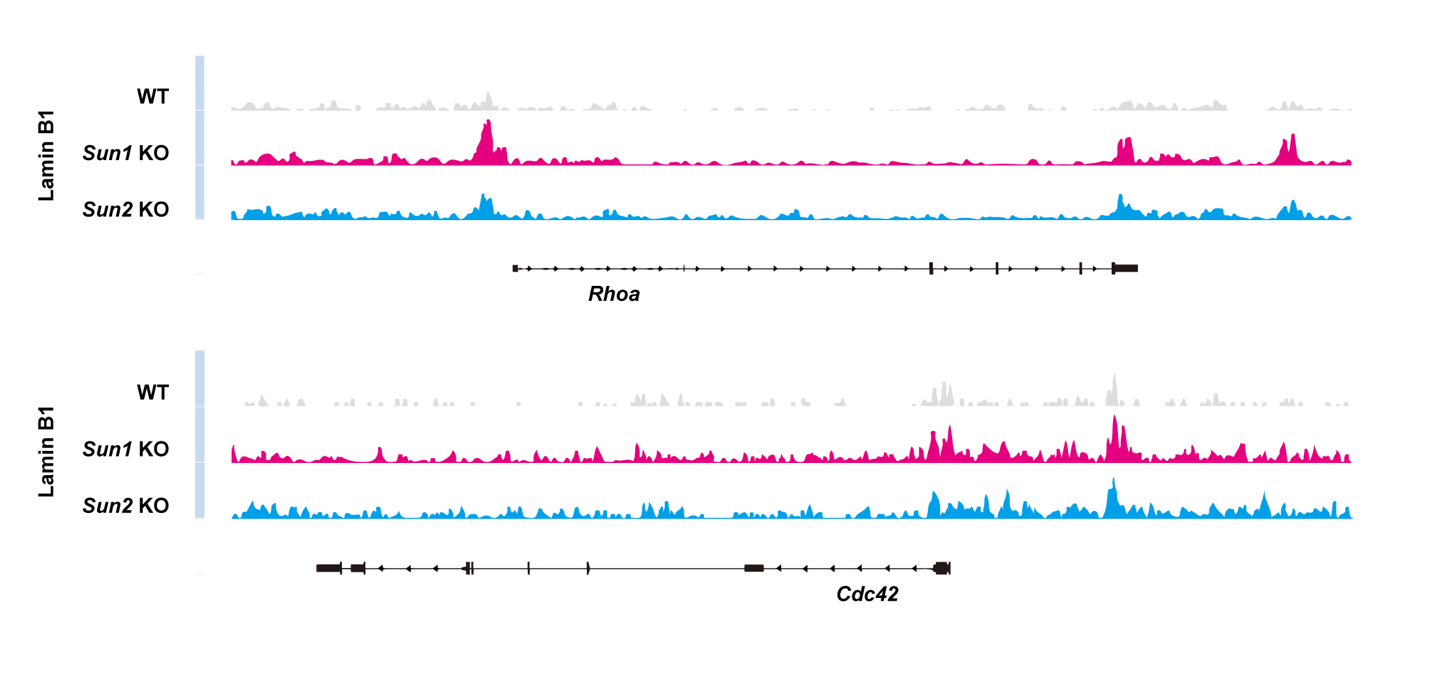


**Supplementary Fig. S10. The alteration of lamin-bound chromatin on adhesion-related genes.**

Gene view of lamin B1 signals in C2C12 WT, *Sun1* KO and *Sun2* KO cells for adhesion-related genes *Rhoa* and *Cdc42*.


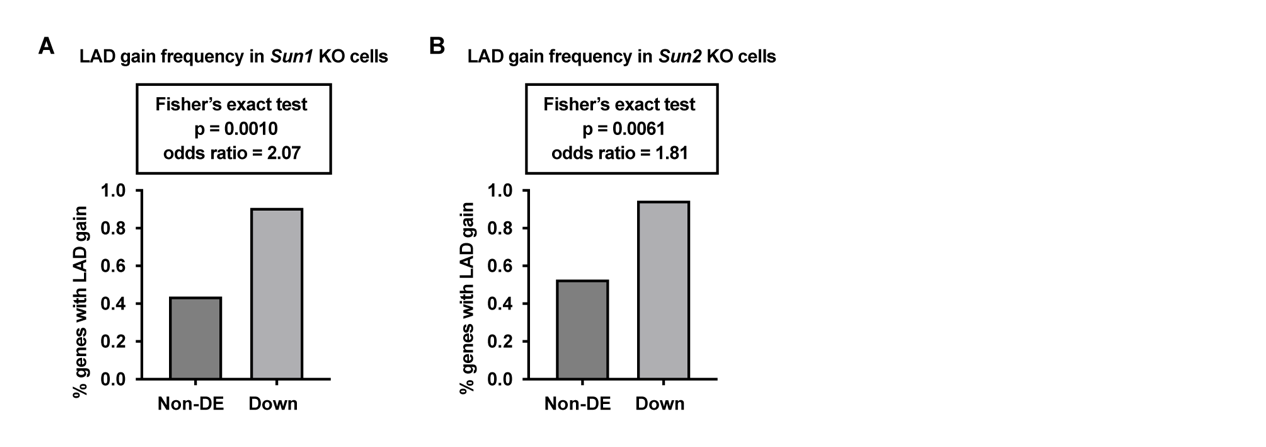


**Supplementary Fig. S11. Enrichment of lamin B1 gain among downregulated genes.**

(A and B) Frequency of lamin B1 gain in downregulated genes compared with non-differentially expressed genes in *Sun1* KO (A) or *Sun2* KO (B) groups. Statistical significance was assessed using Fisher’s exact test.


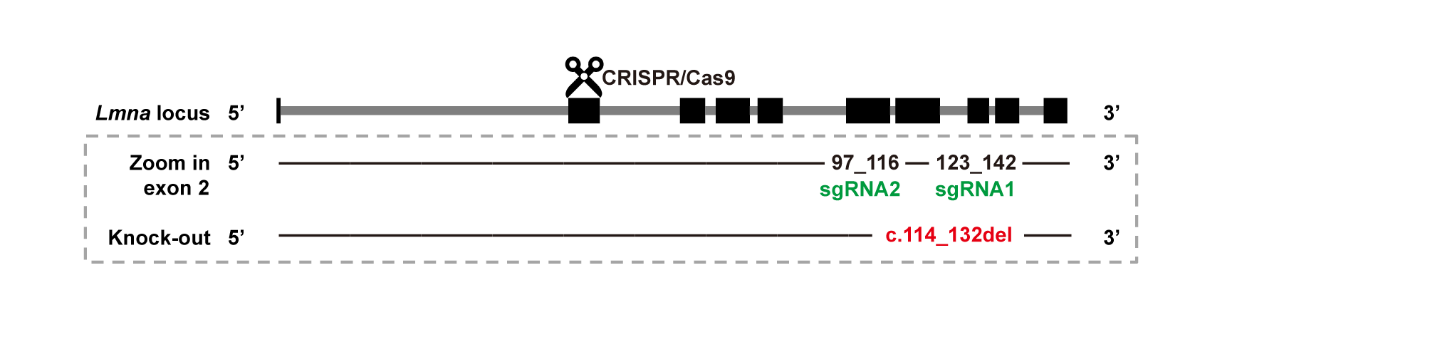


**Supplementary Fig. S12. Schematic of *Lmna* gene knockout.**

Black rectangles represent the exons of *Lmna*. Green indicates the two sgRNA sequences targeting the exon 2, while red marks the deletion mutation site.


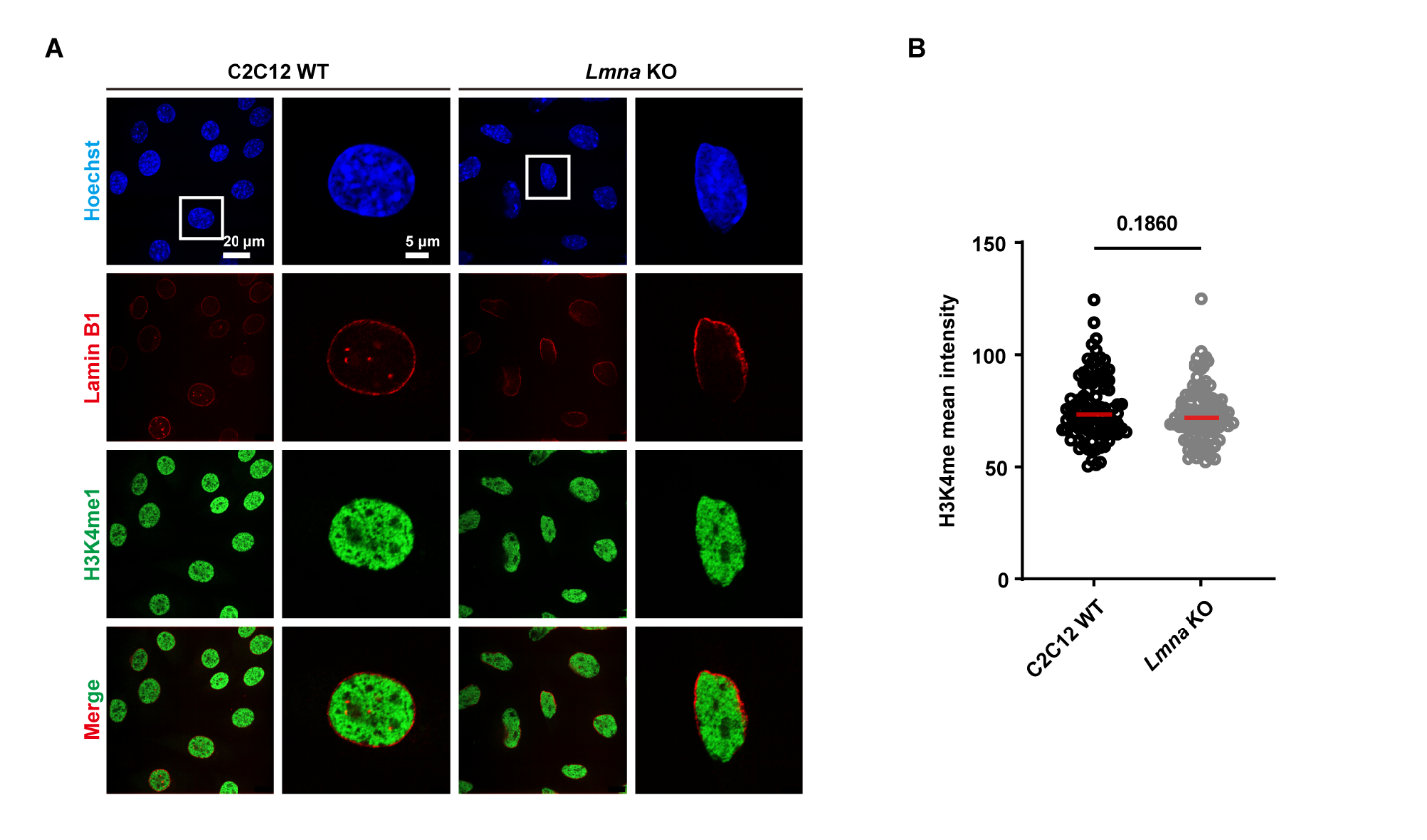


**Supplementary Fig. S13. Lamin A/C loss causes perinuclear defects of lamin B1 but does not change the intensity of H3K4me1.**

(A) Immunofluorescence staining and imaging of the nucleus, H3K4me1, and lamin B1 (H3K4me1, n = 95, 95 cells). Scale bar, 20 μm, 5 μm.

(B) Quantitative analysis of the average fluorescence intensity of H3K4me1. Each dot represents one cell, and the red line segment indicates the mean value. Two-tailed unpaired Student *t*-tests.


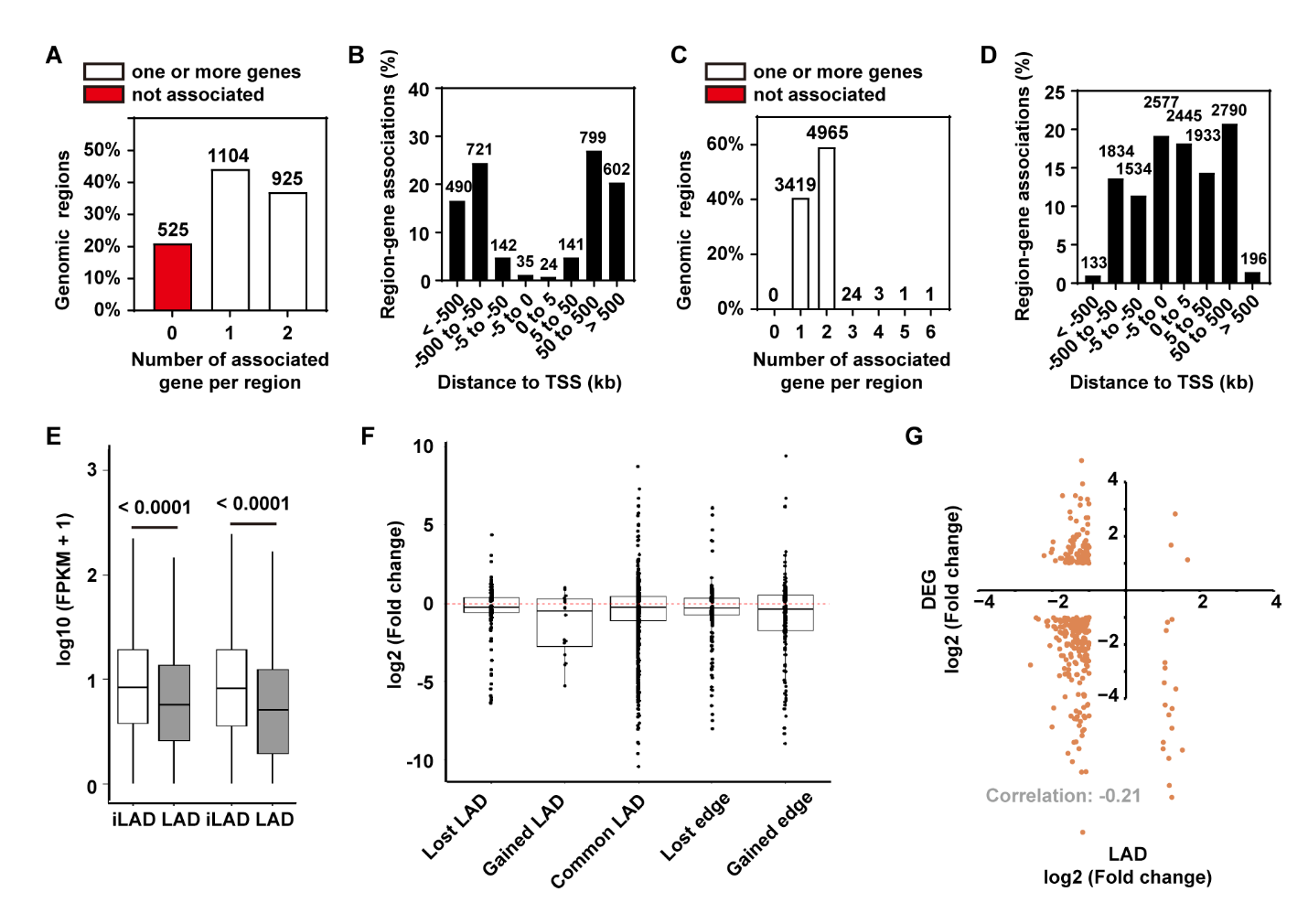


**Supplementary Fig. S14. Alteration in gene characteristics of LAD after *Lmna* knockout.**

(A) Bar chart showing the number of associated genes per sites with significantly increased lamin B1 binding affinity after *Lmna* knockout.

(B) Distance distribution plot showing the distance of each site from the nearest gene region in (A).

(C) Bar chart showing the number of associated genes per sites with significantly decreased lamin B1 binding affinity after *Lmna* knockout.

(D) Distance distribution plot showing the distance of each site from the nearest gene region in (C).

(E) Quantitative analysis of gene expression levels in LAD and i-LAD between C2C12 WT and *Lmna* KO cells. Data are presented as the mean with SD. Two-tailed unpaired Student *t*-tests.

(F) Quantitative analysis of gene expression change fold in the five classes of LAD. Data are presented as the mean with SD. Each dot represents a LAD, and red line segments indicate the 0 values.

(G) The quadrant plot displays genes with significant changes in lamin B1 affinity (LAD) and genes with significant changes in transcriptional expression (DEG).


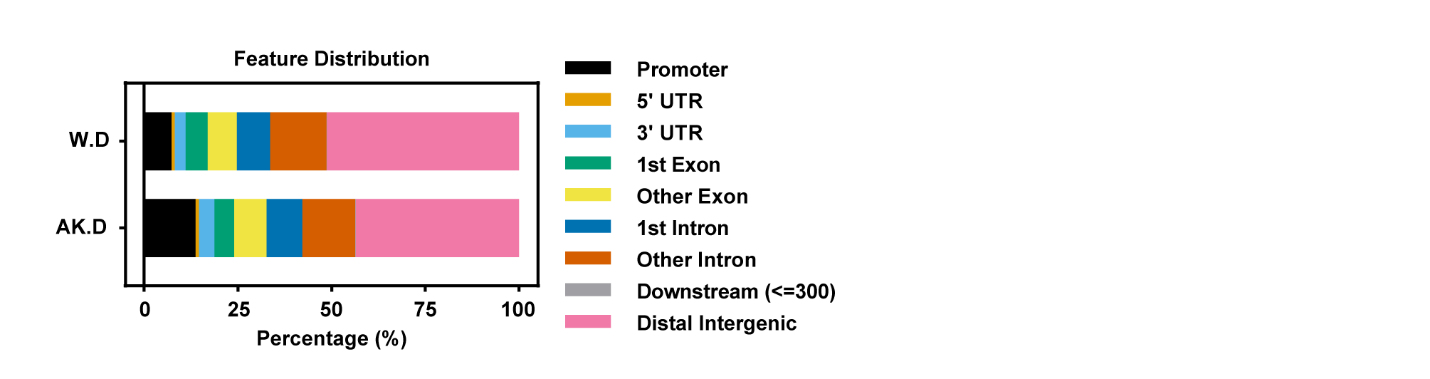


**Supplementary Fig. S15. The proportion of H3K9me2 binding in promoter region increases after *Lmna* knockdown.**

Percentage distribution of gene characteristics associated with H3K9me2 binding sites in C2C12 WT and *Lmna* KO cells. Gene characteristics include promoters, UTRs, exons, introns, and distal intergenic regions, among others. W.D represents H3K9me2 binding sites in C2C12 WT, and A.D represents H3K9me2 binding sites in *Lmna* KO cell.


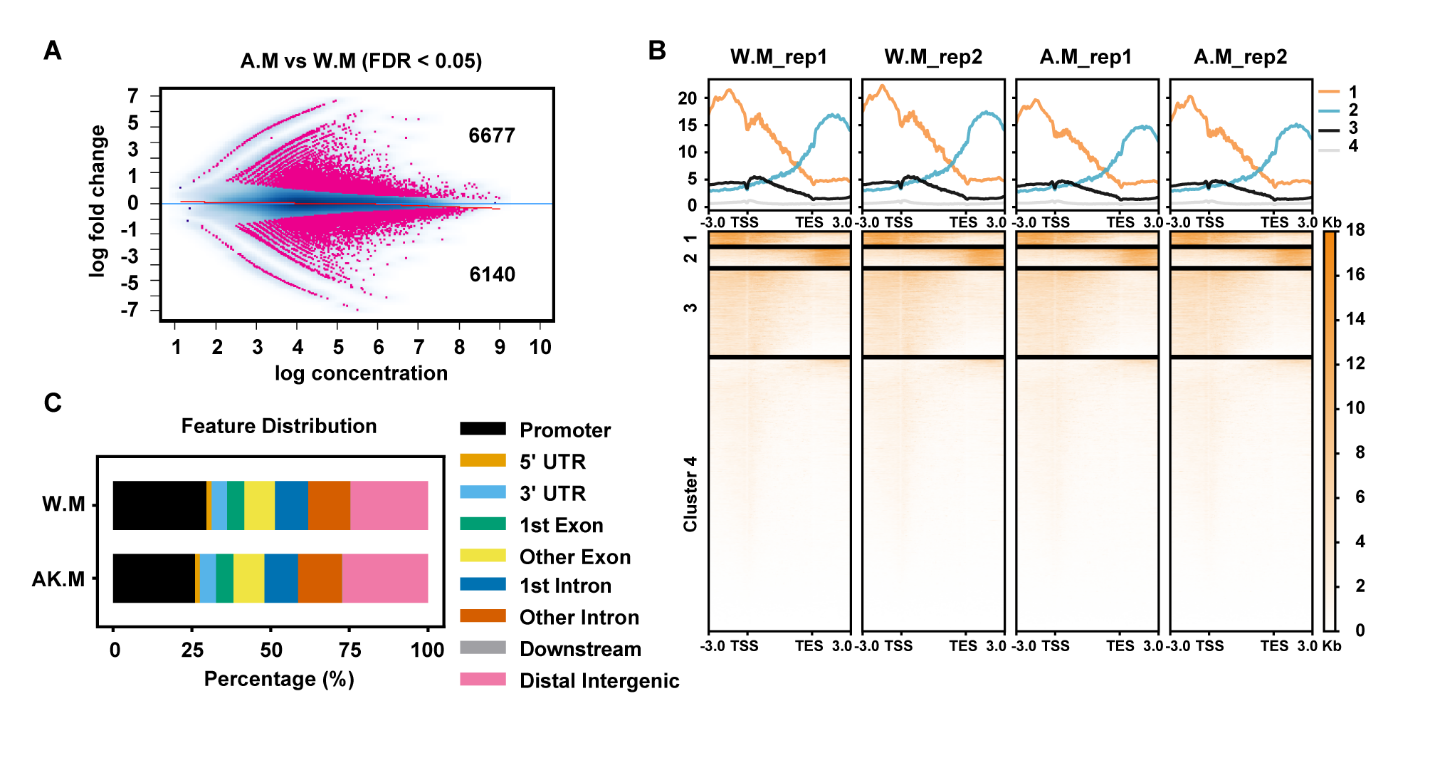


**Supplementary Fig. S16. The proportion of H3K4me1 binding in promoter region decreases after *Lmna* knockout.**

(A) The Bland-Altman plot illustrates the H3K4me1 binding sites with significant changes in affinity after *Lmna* knockout. Each dot represents a site. The number of sites in each region is labeled accordingly.

(B) The line graph above shows the signal distribution of H3K4me1 across the entire genome and its up/downstream 3 kb regions in C2C12 WT and *Lmna* KO cells (two replicates per group). The total signals were clustered into four categories. Below, the heatmap displays the H3K4me1 signal values around each gene within each cluster, sorted from highest to lowest.

(C) Percentage distribution of gene characteristics associated with H3K4me1 binding sites in C2C12 WT and *Lmna* cells. Gene characteristics include promoters, UTRs, exons, introns, and distal intergenic regions, among others.

W.M represents H3K4me1 binding sites in C2C12 WT, and A.M represents H3K4me1 binding sites in *Lmna* KO cell in all the figures.


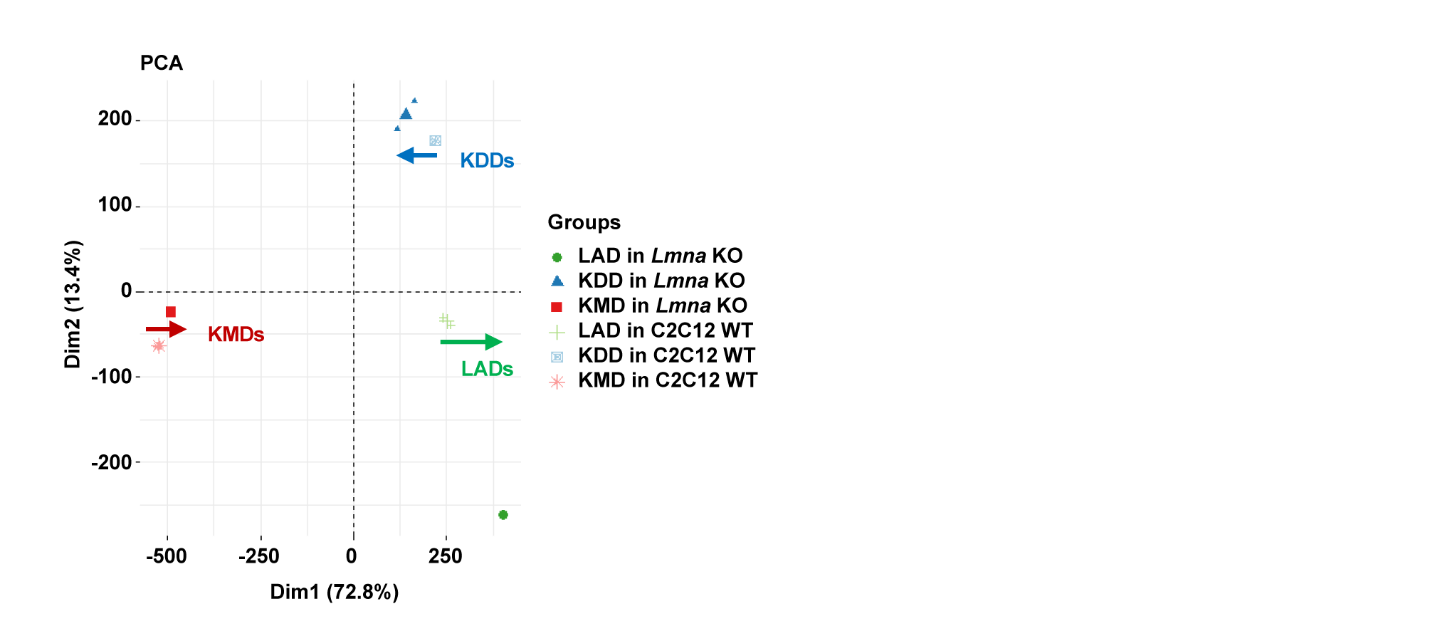


**Supplementary Fig. S17. Alteration of histones and lamin-bound chromatin led to inhibition of adhesion-related genes.**

PCA plot showing the similarity in structural domain features of LADs, KDDs and KMDs between C2C12 WT and *Lmna* cells.


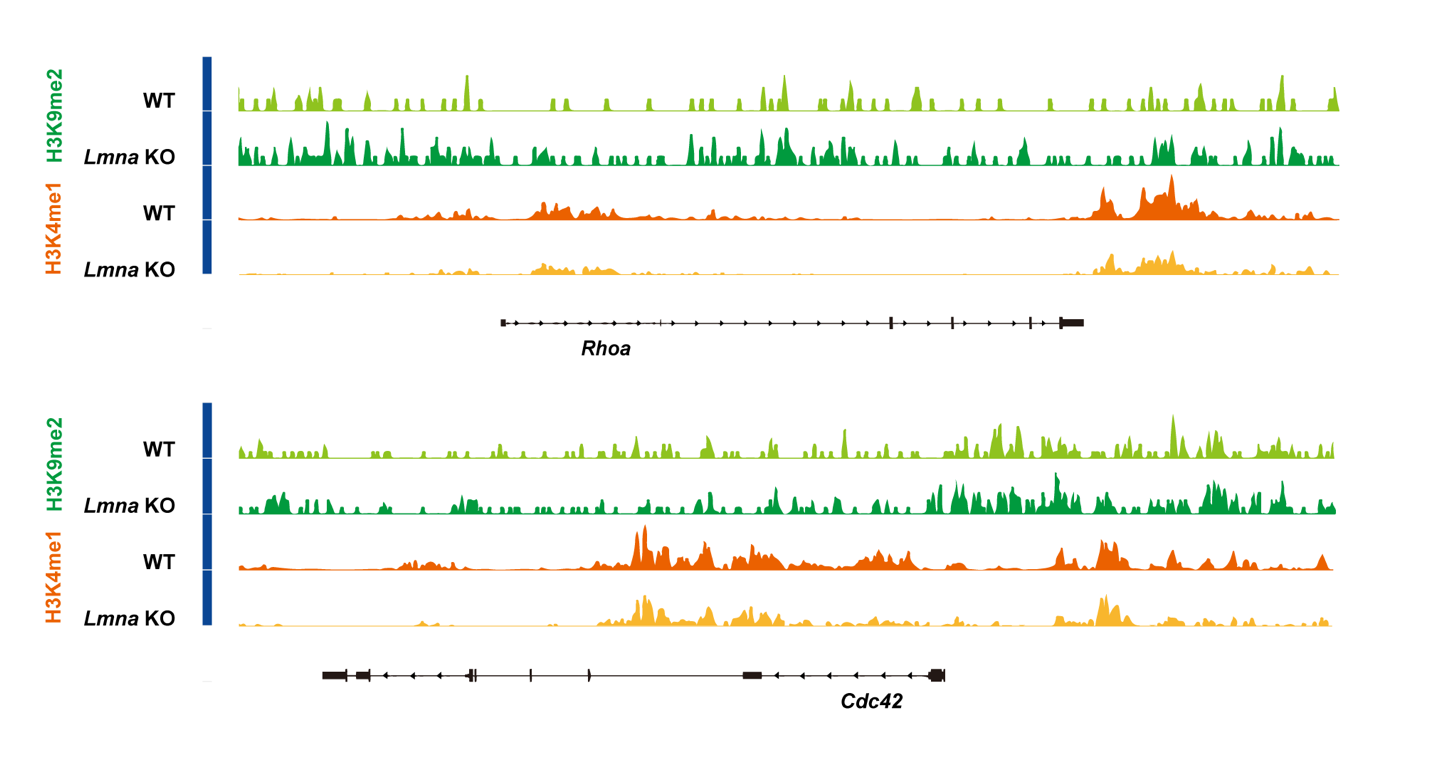


**Supplementary Fig. S18. The histone modifications binding with adhesion-related genes.**

Gene view of H3K9me2 and H3K4me1 signals in C2C12 WT and *Lmna* KO cells for adhesion-related genes *Rhoa* and *Cdc42*.


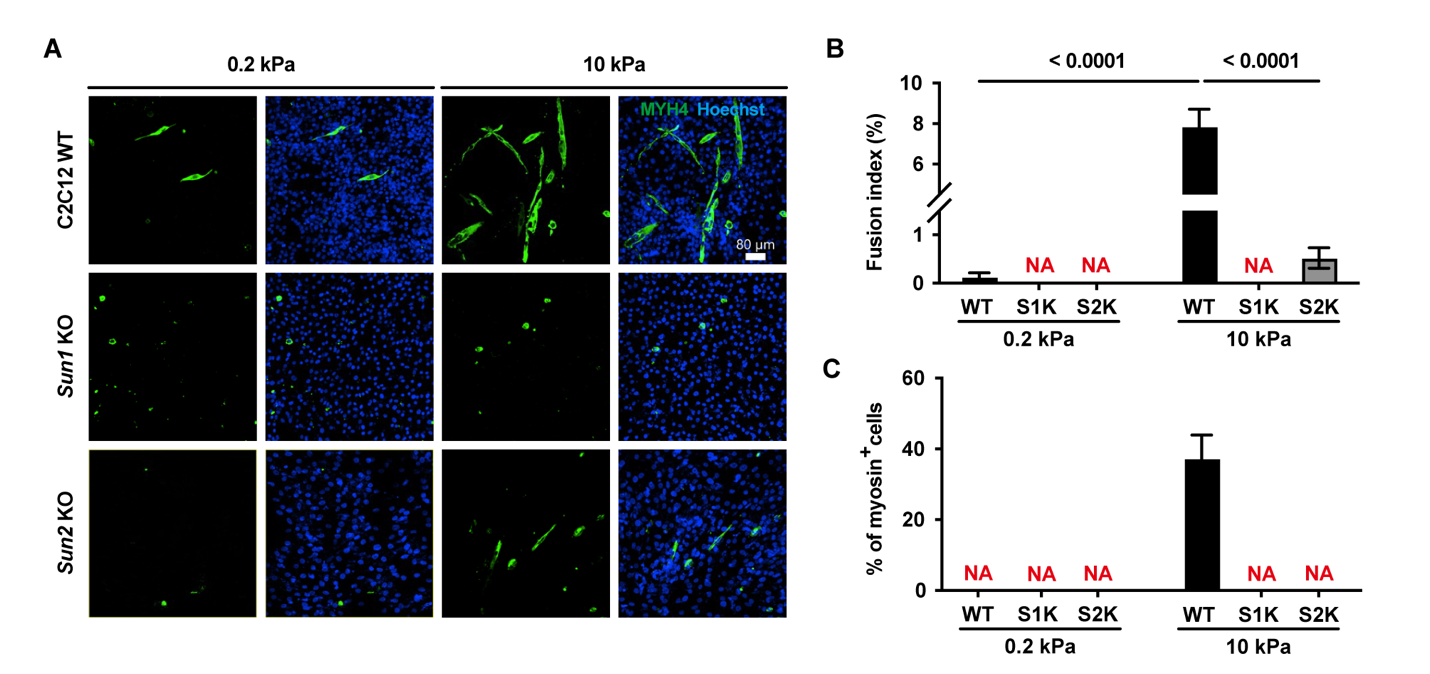


**Supplementary Fig. S19. SUN1/2 prime stiffness-dependent myogenic differentiation.**

(A) Immunofluorescence staining and imaging of nuclei and MYH4 in C2C12 WT, *Sun1* KO, and *Sun2* KO cells cultured under hard (10 kPa) and soft (0.2 kPa) conditions for six days (n = 5, 5, 5, 5, 5, 5 views). Scale bar, 80 μm.

(B) Quantitative analysis of the ratio of fused myotube nuclei to total nuclei in each group. Data are presented as the mean with SD. Two-tailed unpaired Student t-tests.

(C) Quantitative analysis of the ratio of nuclei within individual myotubes (nuclei count > 3) to total nuclei across all myotubes. Data are presented as the mean with SD.


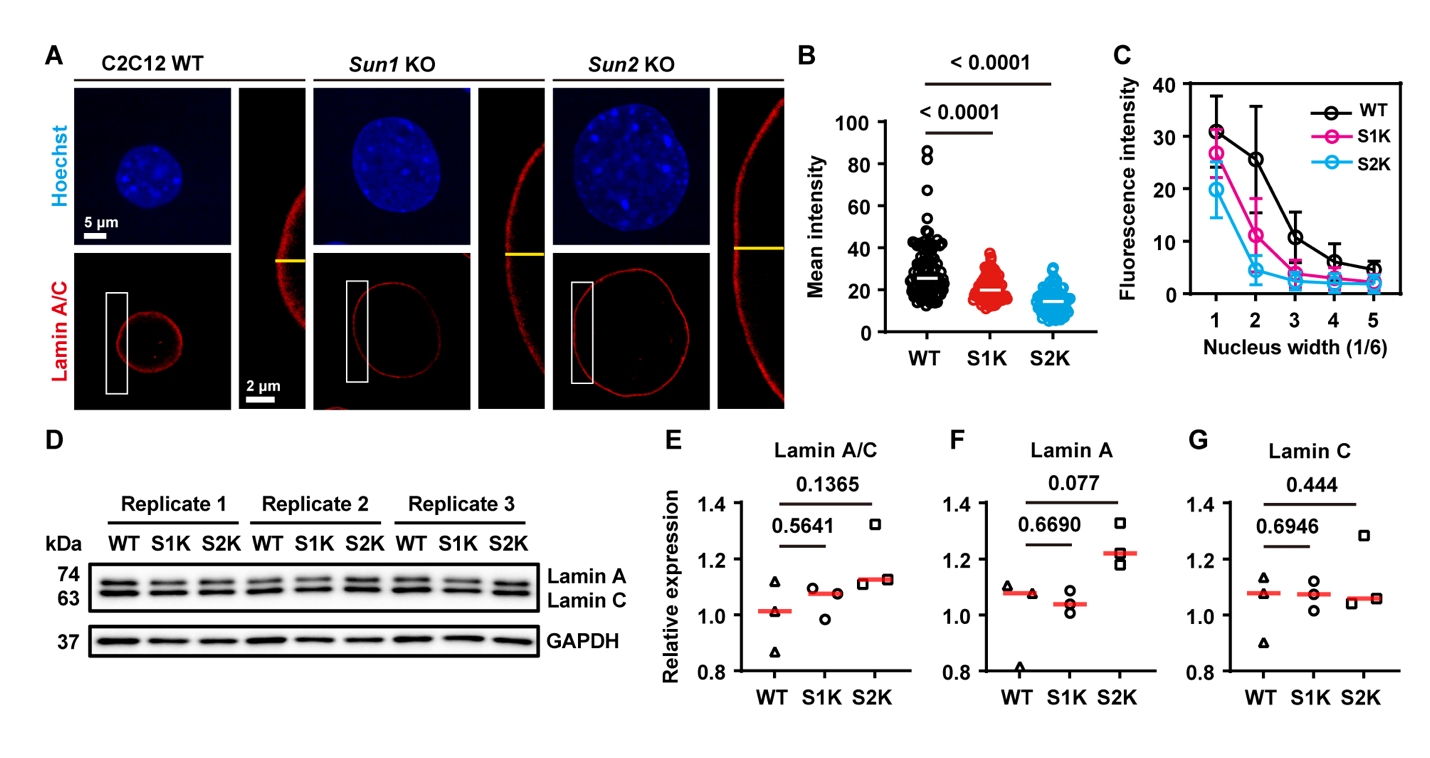


**Supplementary Fig. S20. Perinuclear lamin A/C decreases upon *Sun1* or *Sun2* knockout.**

(A) Immunofluorescence staining and imaging of nuclei and lamin A/C in C2C12 WT, *Sun1* KO, and *Sun2* KO cells (lamin A/C, n = 98, 97, 75 cells). Scale bar, 5 μm, 2 μm. The yellow line is one-sixth of the cell diameter.

(B) Quantification of lamin A/C mean intensity. Each dot represents a biological replicate, and white line segments indicate the mean values. One-way ANOVA and Tukey's HSD test.

(C) Line graph showing the distribution of lamin A/C fluorescence intensity across the nuclear periphery (white line segments). The x-axis divides the white line region into five equal regions. Data are presented as the mean with SD.

(D) WB analysis of lamin A/C protein levels in C2C12 WT, *Sun1* KO, and *Sun2* KO cells (three biological replicates per group).

(E-G) Quantification of lamin A/C (E), lamin A (F), lamin C (G) protein levels. Each dot represents a biological replicate, and red line segments indicate the mean values. One-way ANOVA and Tukey's HSD test.


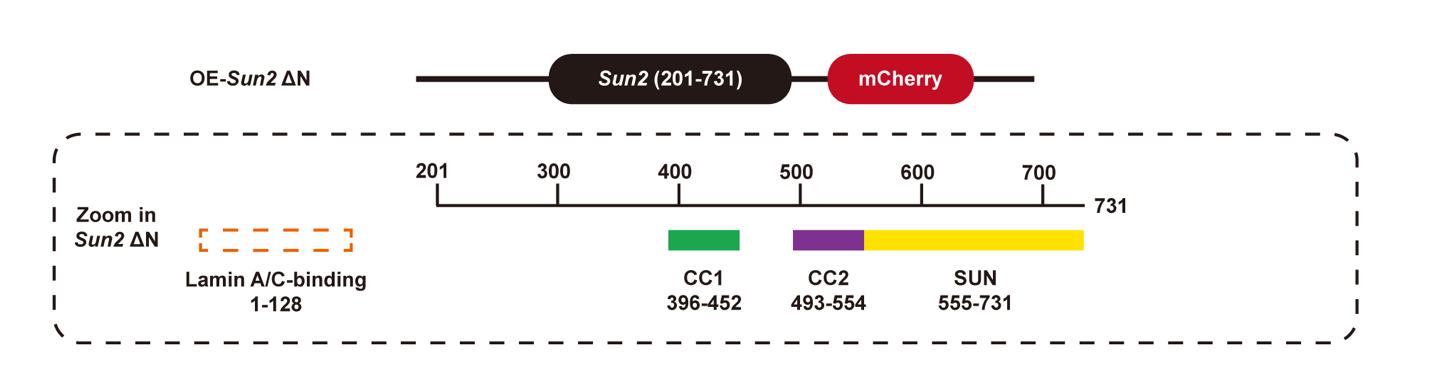


**Supplementary Fig. S21. Disruption of the SUN2-Lamin A/C interaction in the expression of upstream mechanotransduction-related genes.**

(A) The protein architecture and domain composition of the N-terminally truncated mutant *Sun2* ΔN used for overexpression. The *Sun2* ΔN (residues 201–731) overexpression construct (OE-*Sun2* ΔN), which lacks the N-terminus, loses the ability to interact with lamin A/C. A zoomed-in view annotates the key functional domains of each protein, including the lamin A/C-binding region, the coiled-coil (CC) domain, and the SUN domain, along with their corresponding amino acid residue numbers. mCherry was fused to the C-terminus of the proteins for live-cell imaging and subcellular localization analysis.
