# Supplementary figures and images for: A Mechano-Feedback Loop Orchestrated by SUN1/2 Governs Cellular Mechanoadaptation via Lamina-Associated Domain Remodeling
Source: Research (Wash D C). 2026 May 14;9:1259. doi: 10.34133/research.1259 (PMC13172581; doi:10.34133/research.1259)

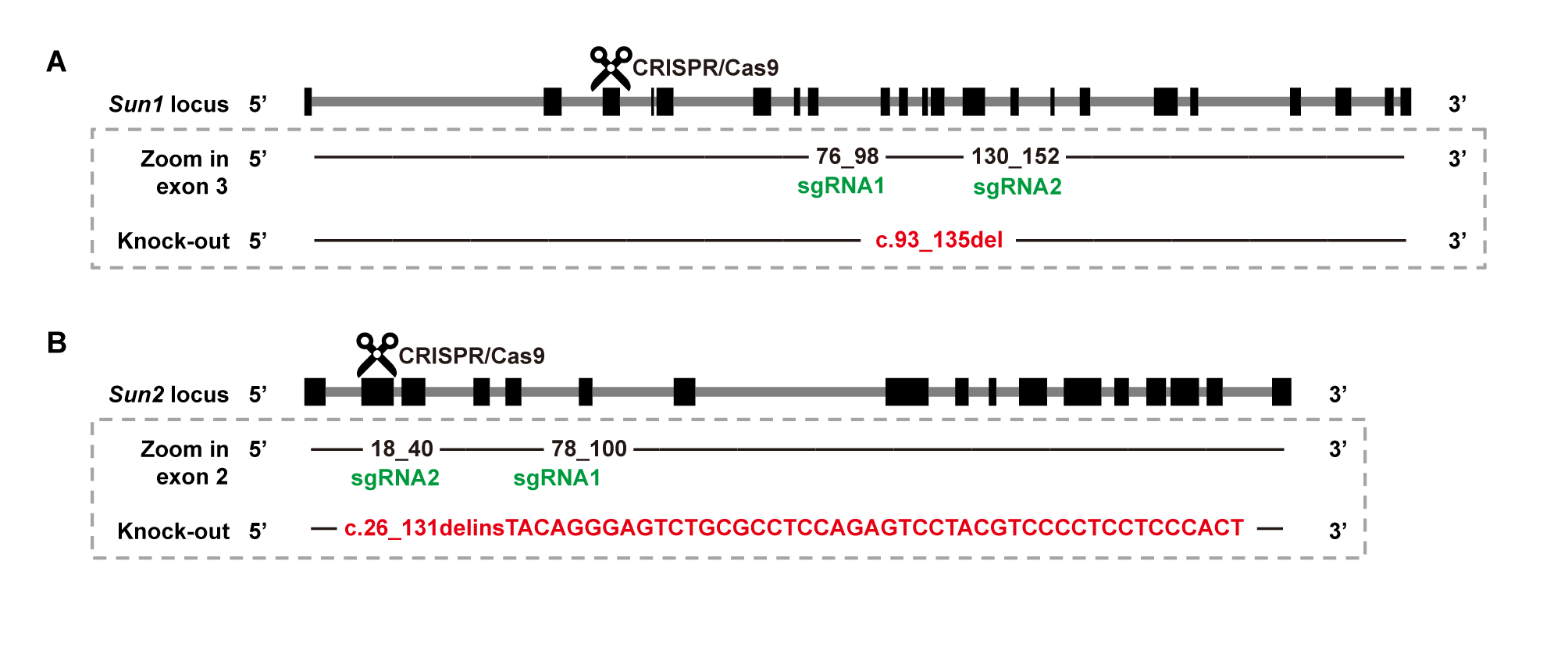

Supplement: Supplementary 1 — Figs. S1 to S21 [file research.1259.f1.zip › FigS1.tif]

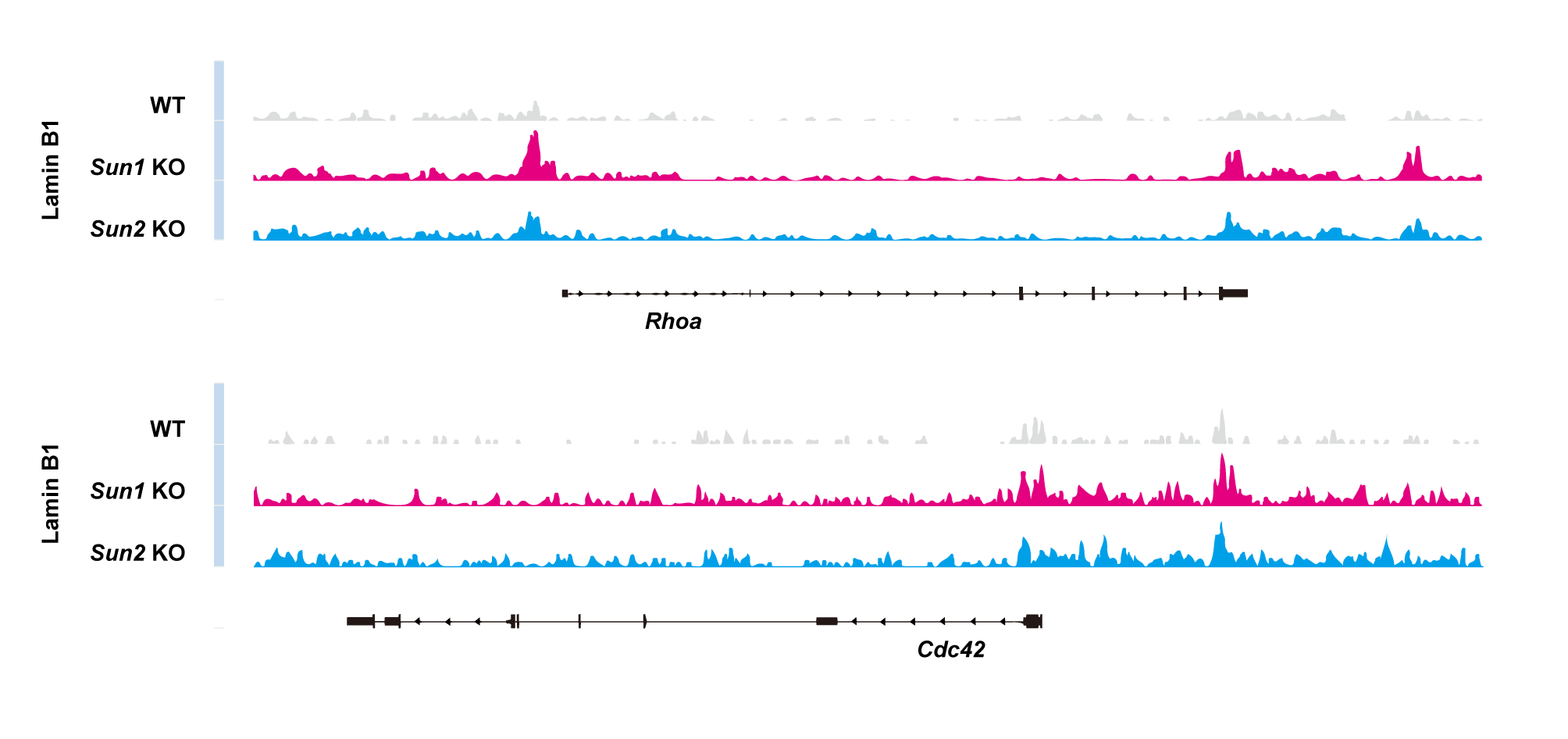

Supplement: Supplementary 1 — Figs. S1 to S21 [file research.1259.f1.zip › FigS10.tif]

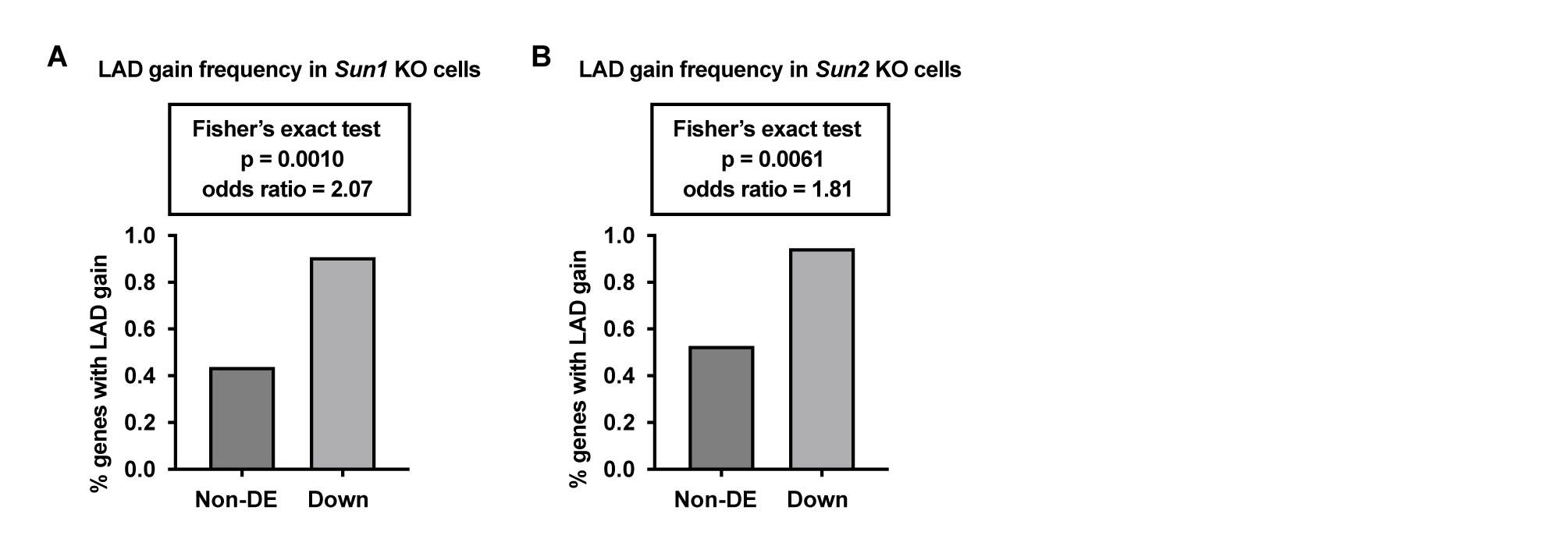

Supplement: Supplementary 1 — Figs. S1 to S21 [file research.1259.f1.zip › FigS11.tif]

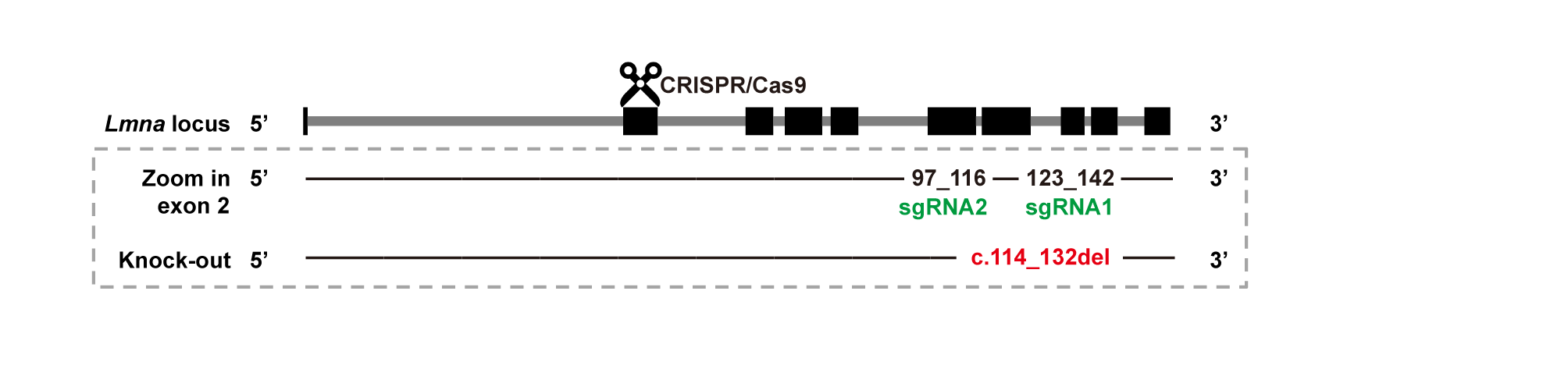

Supplement: Supplementary 1 — Figs. S1 to S21 [file research.1259.f1.zip › FigS12.tif]

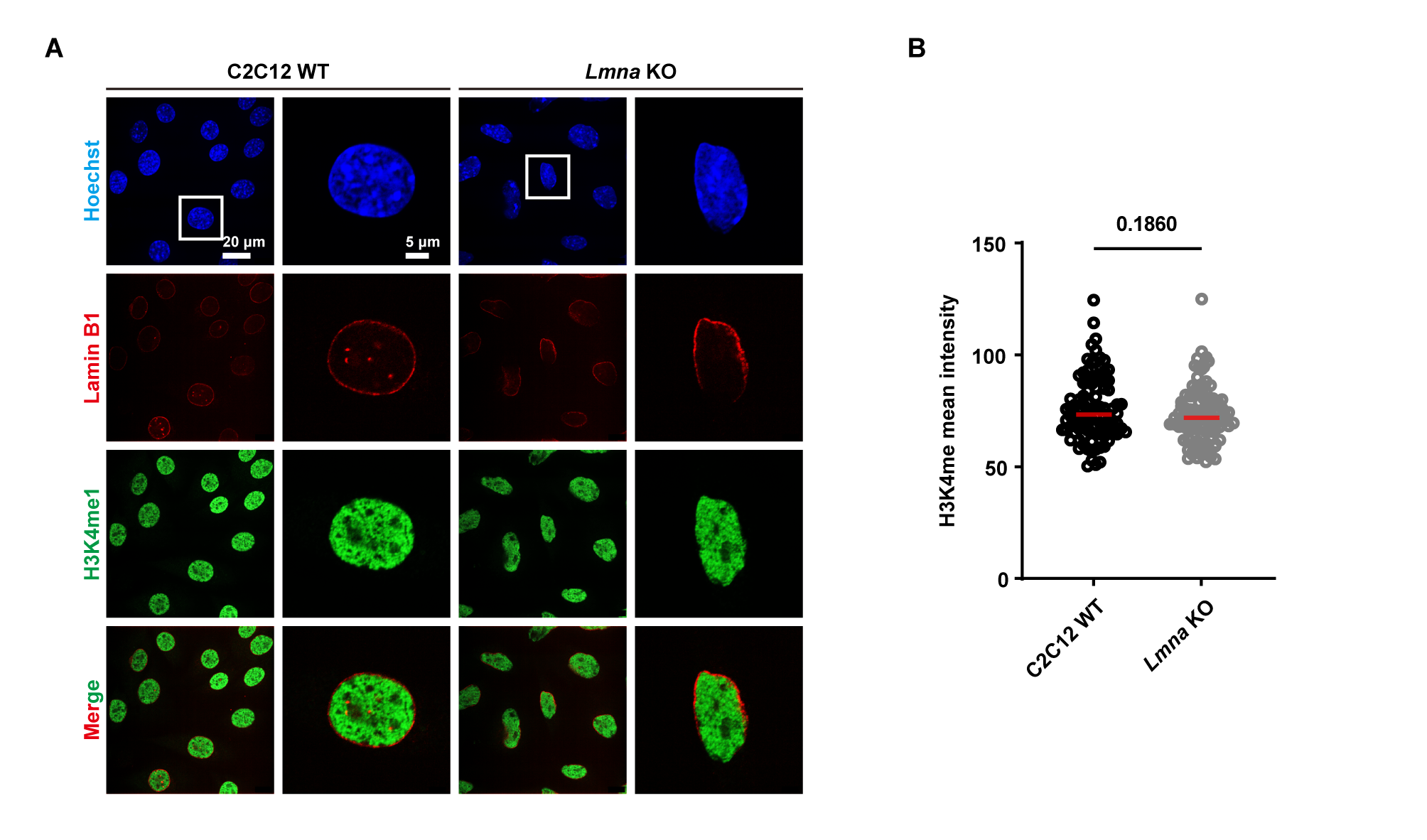

Supplement: Supplementary 1 — Figs. S1 to S21 [file research.1259.f1.zip › FigS13.tif]

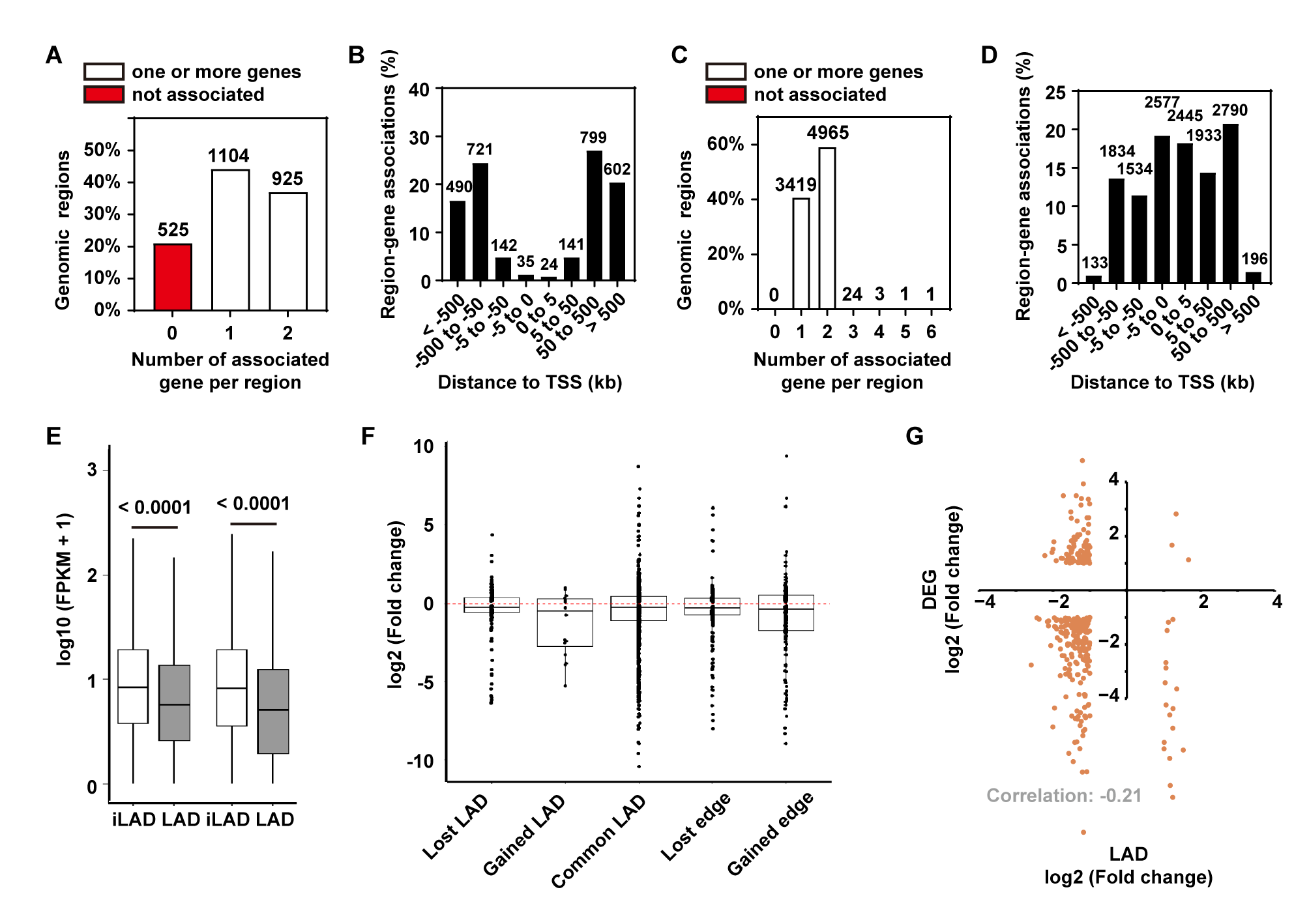

Supplement: Supplementary 1 — Figs. S1 to S21 [file research.1259.f1.zip › FigS14.tif]

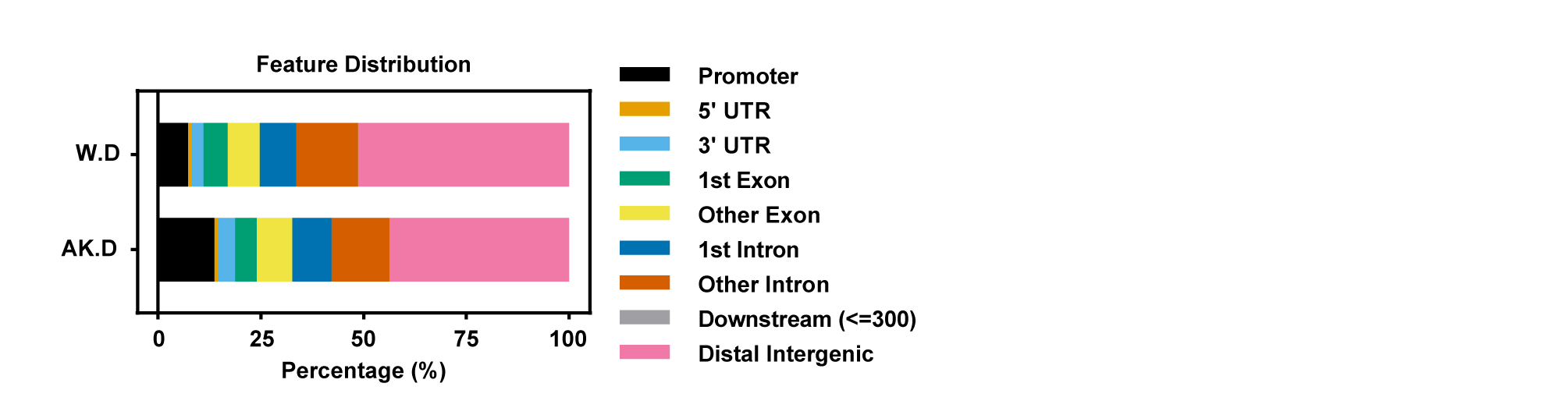

Supplement: Supplementary 1 — Figs. S1 to S21 [file research.1259.f1.zip › FigS15.tif]

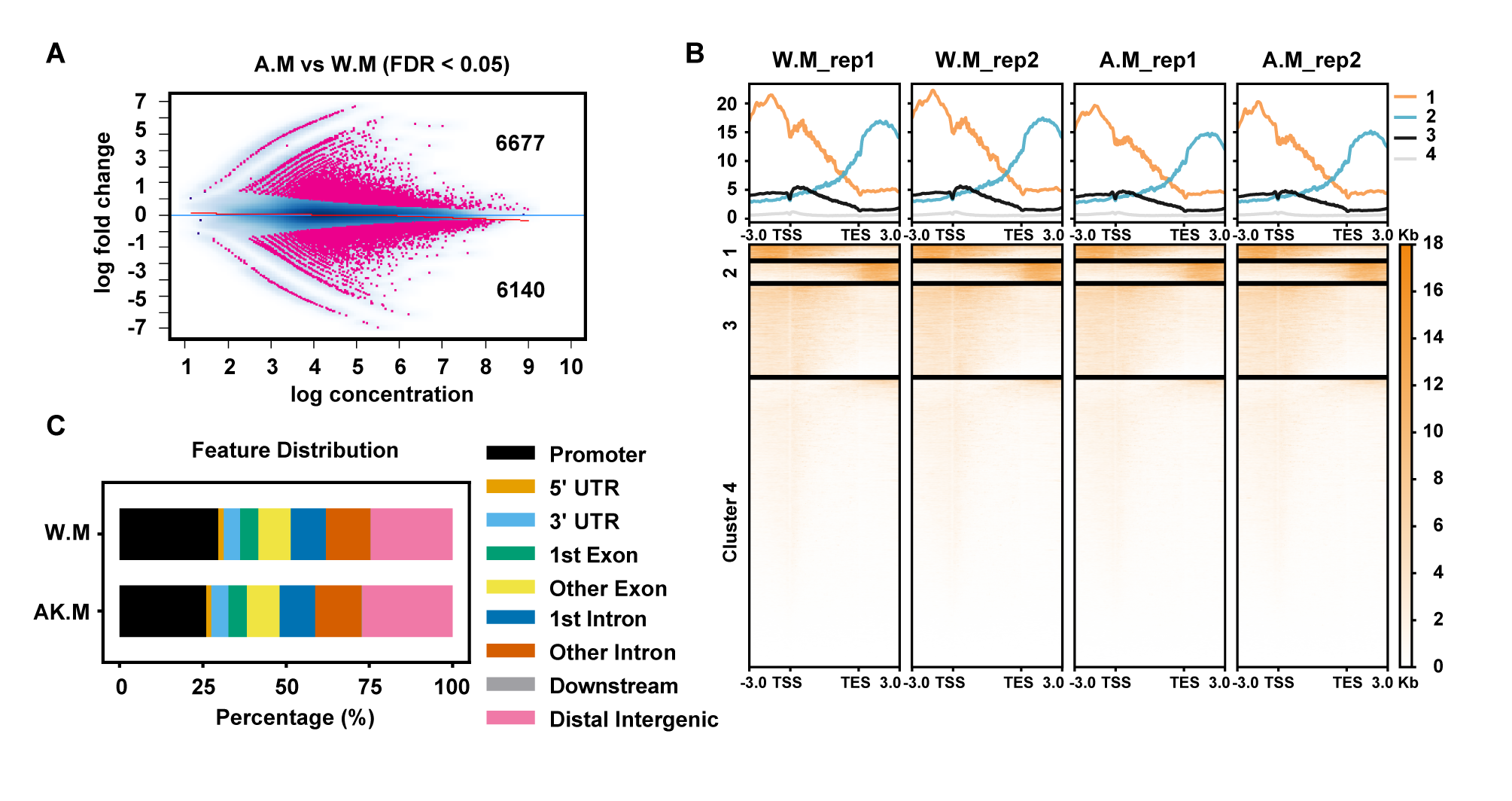

Supplement: Supplementary 1 — Figs. S1 to S21 [file research.1259.f1.zip › FigS16.tif]

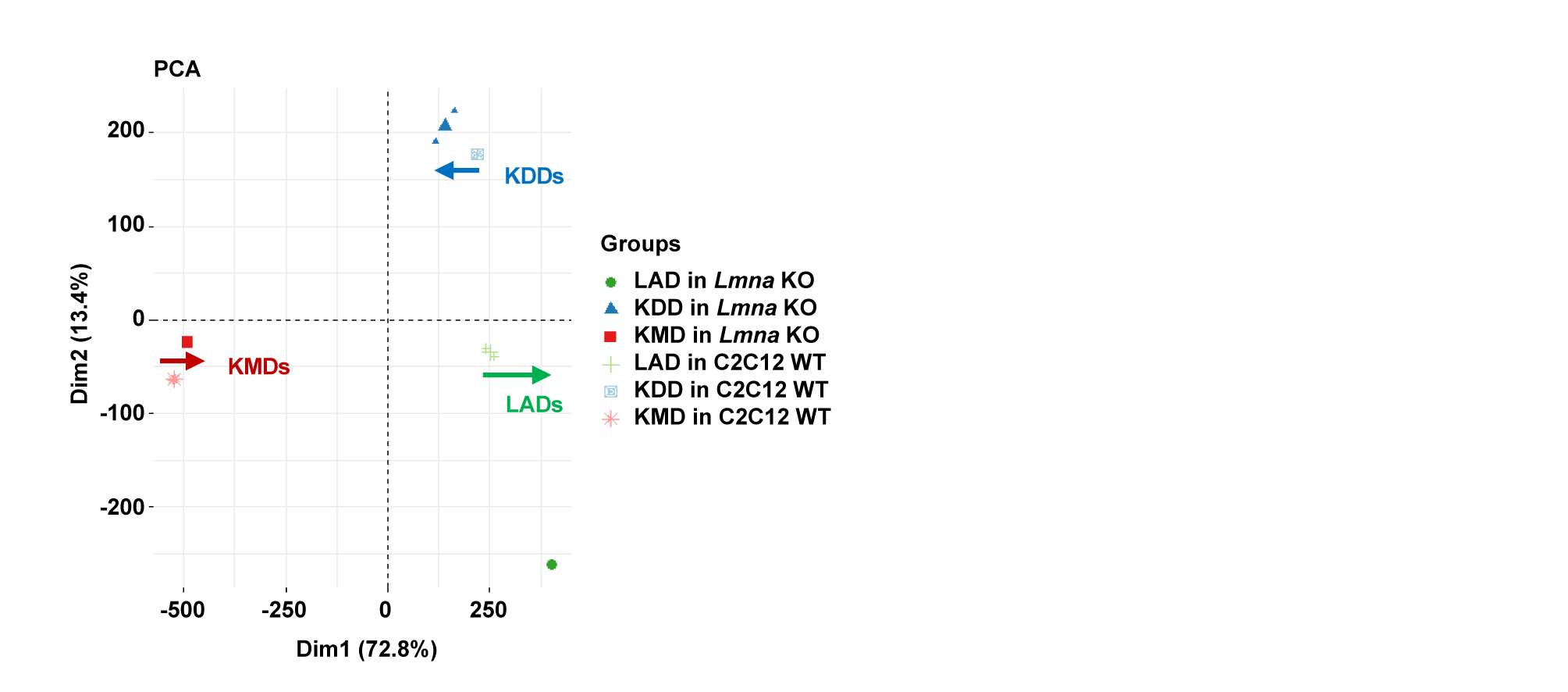

Supplement: Supplementary 1 — Figs. S1 to S21 [file research.1259.f1.zip › FigS17.tif]

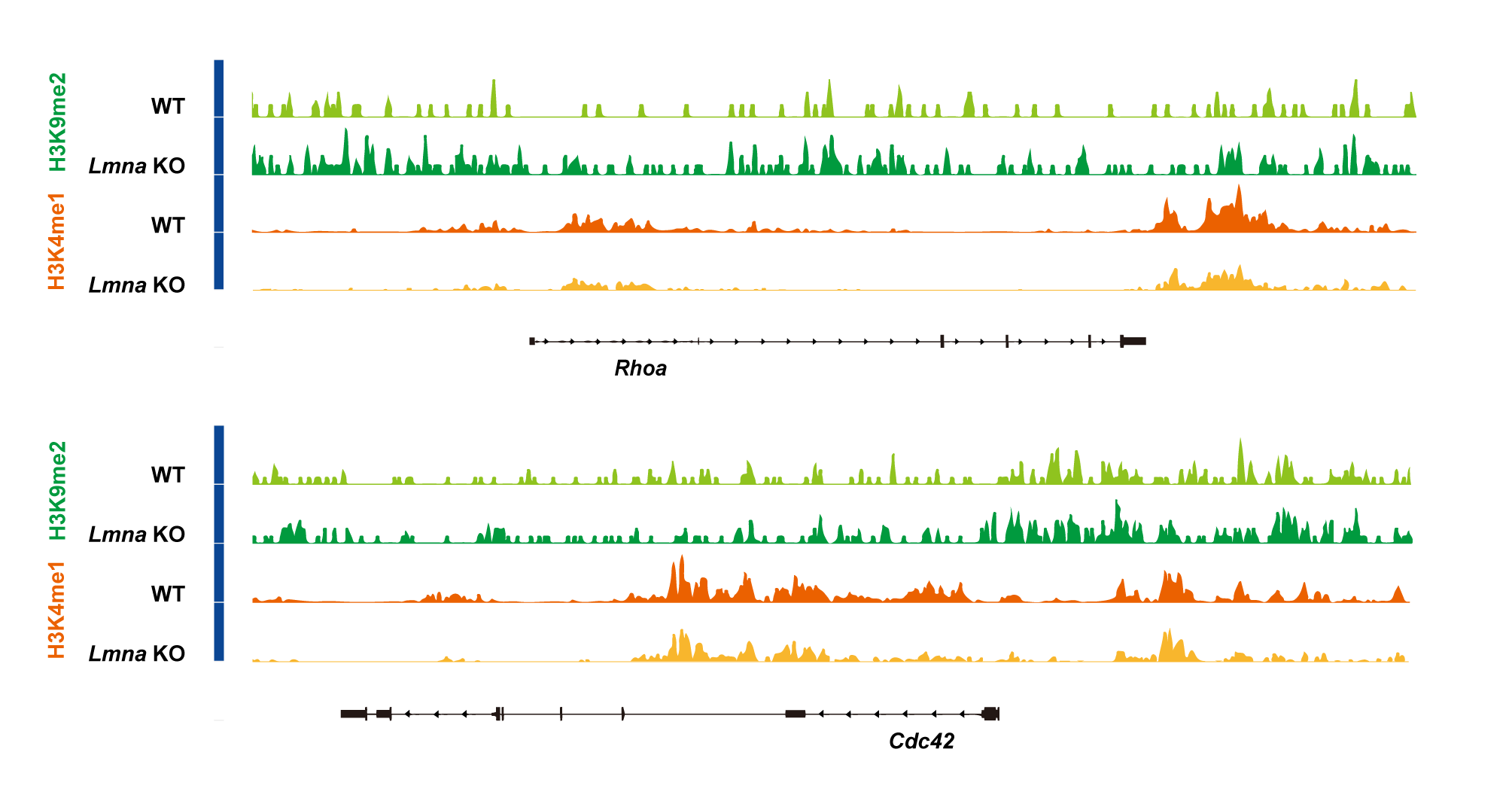

Supplement: Supplementary 1 — Figs. S1 to S21 [file research.1259.f1.zip › FigS18.tif]

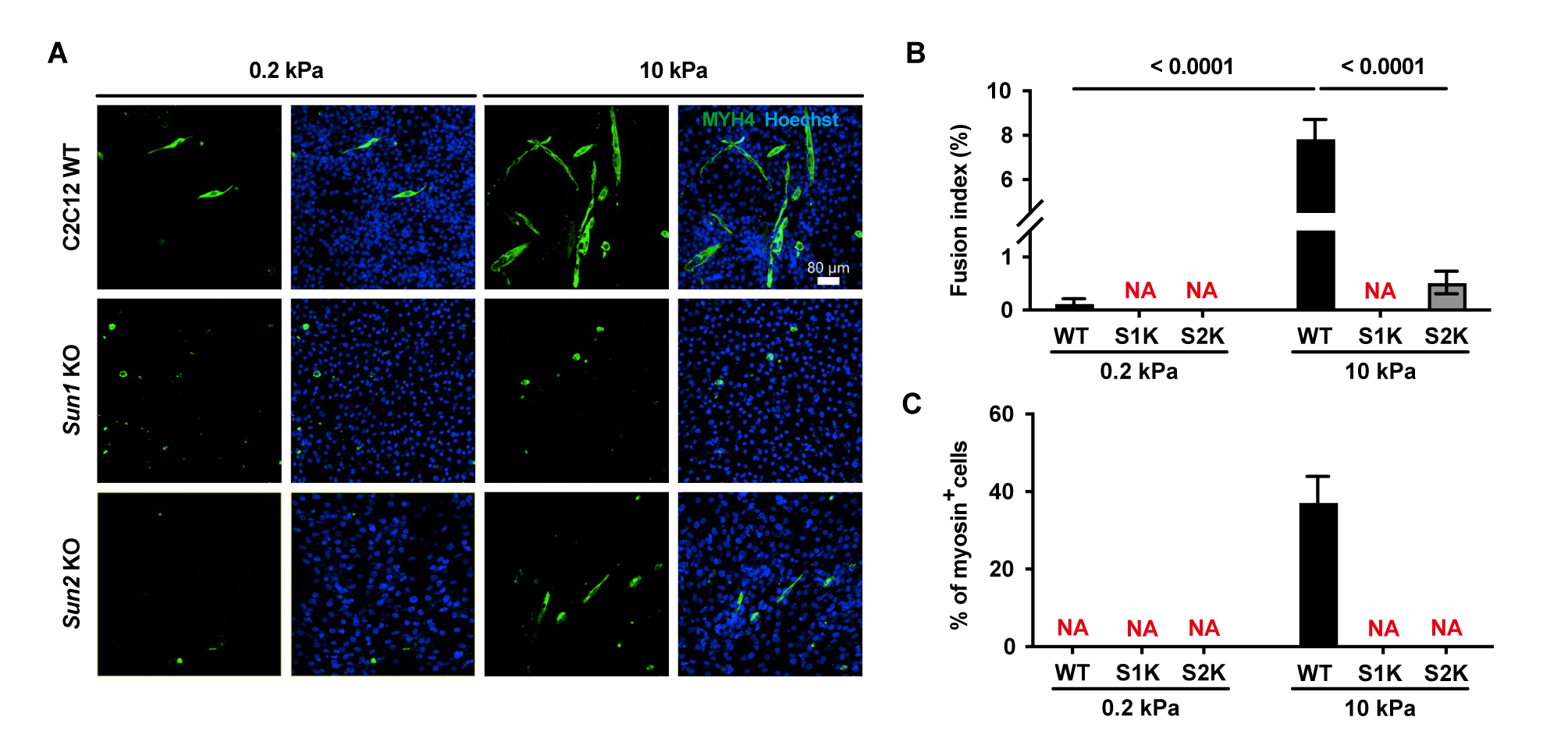

Supplement: Supplementary 1 — Figs. S1 to S21 [file research.1259.f1.zip › FigS19.tif]

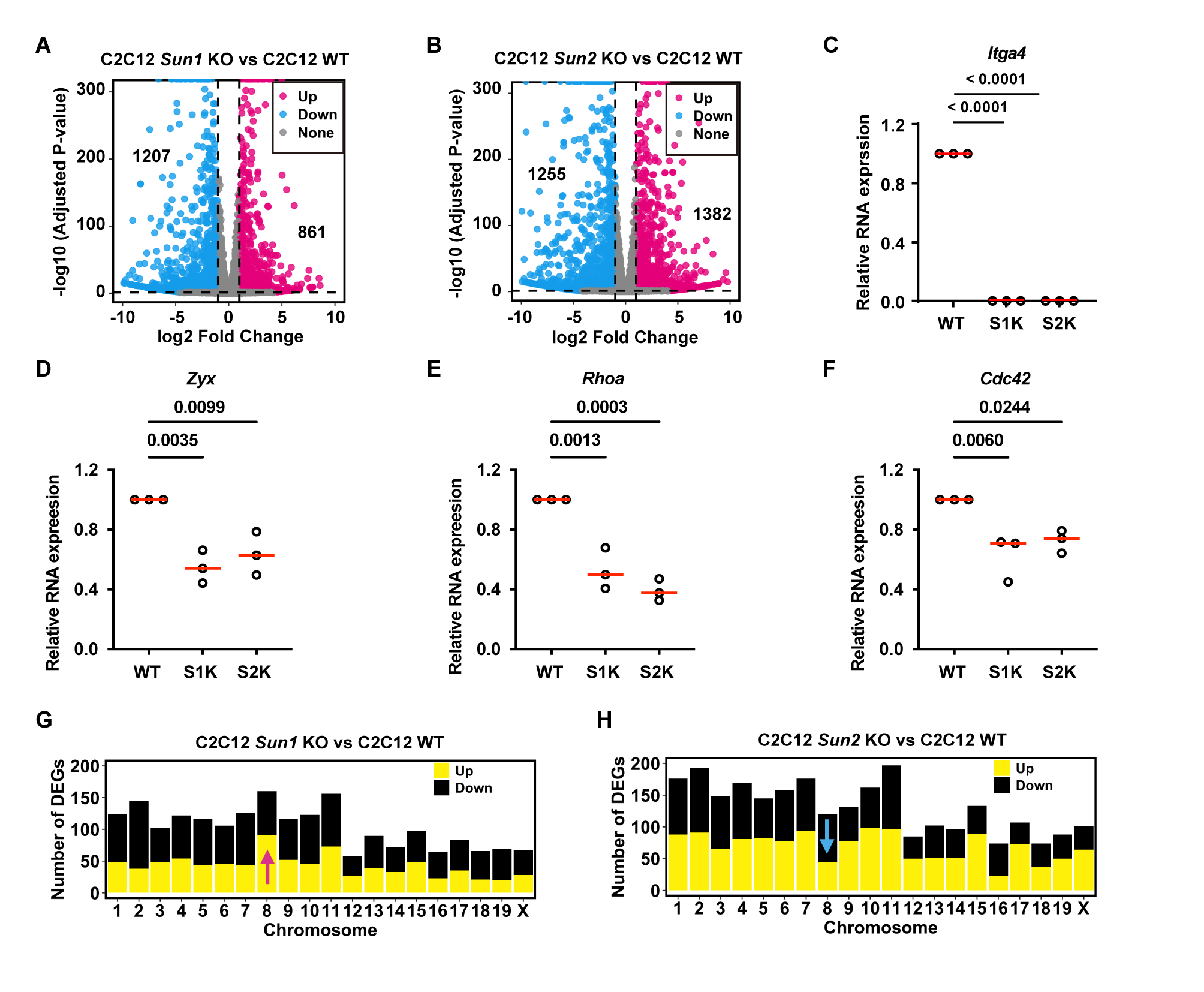

Supplement: Supplementary 1 — Figs. S1 to S21 [file research.1259.f1.zip › FigS2.tif]

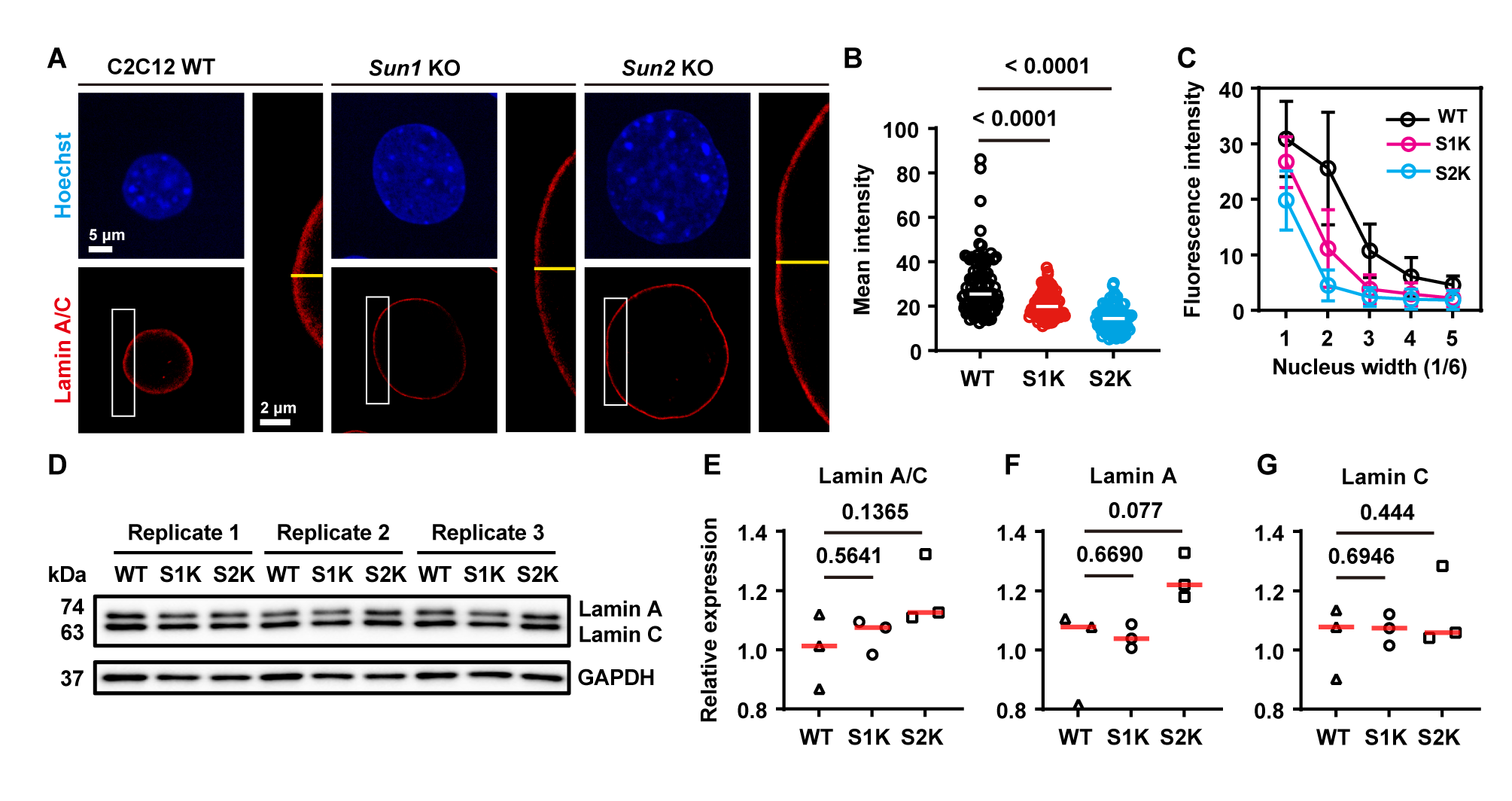

Supplement: Supplementary 1 — Figs. S1 to S21 [file research.1259.f1.zip › FigS20.tif]

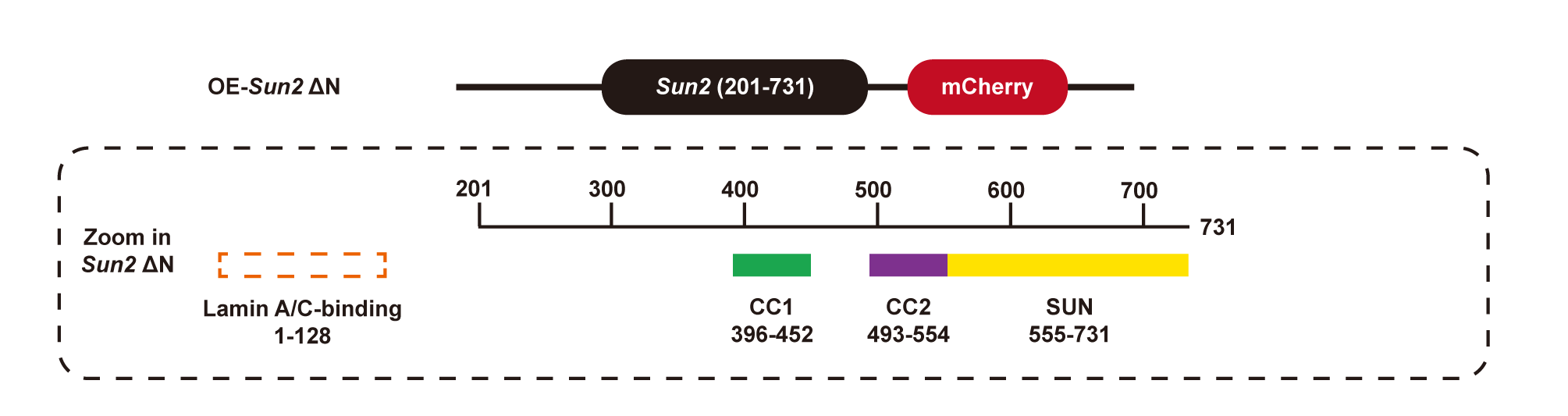

Supplement: Supplementary 1 — Figs. S1 to S21 [file research.1259.f1.zip › FigS21.tif]

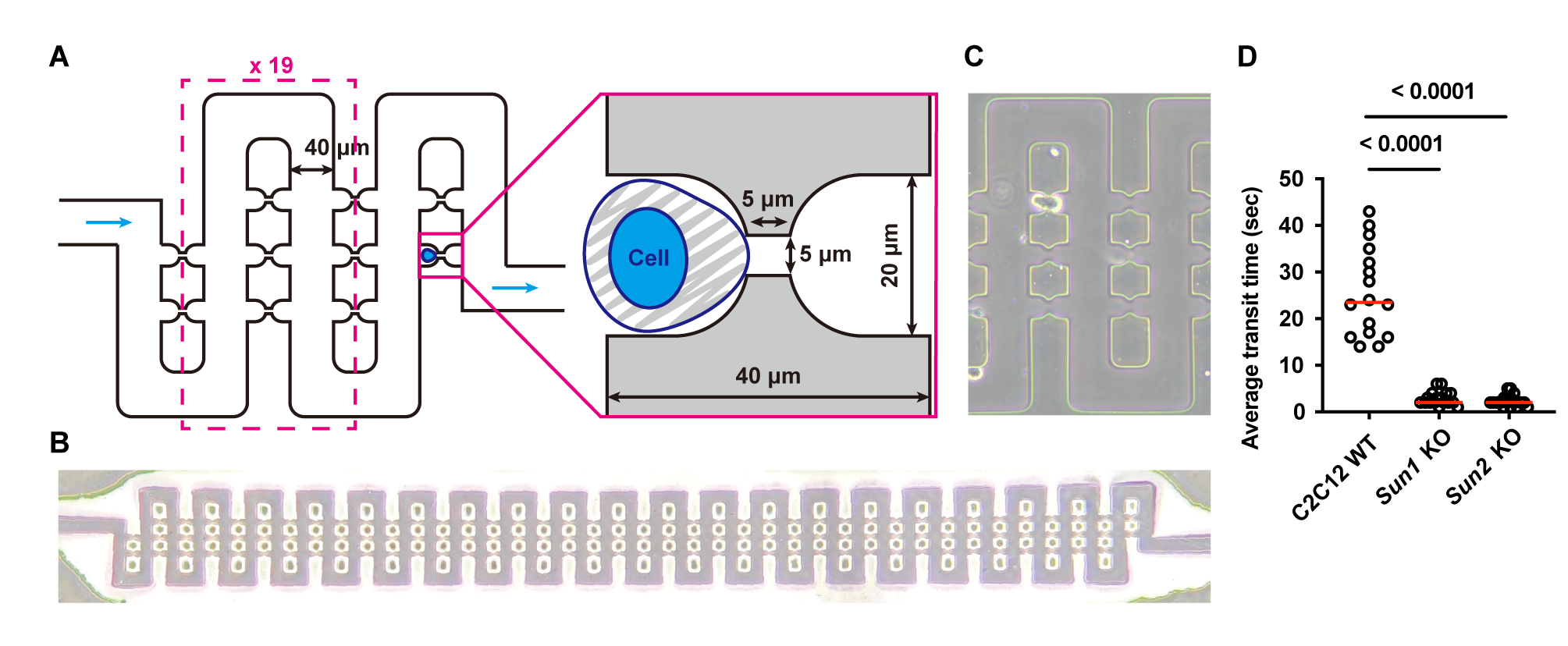

Supplement: Supplementary 1 — Figs. S1 to S21 [file research.1259.f1.zip › FigS3.tif]

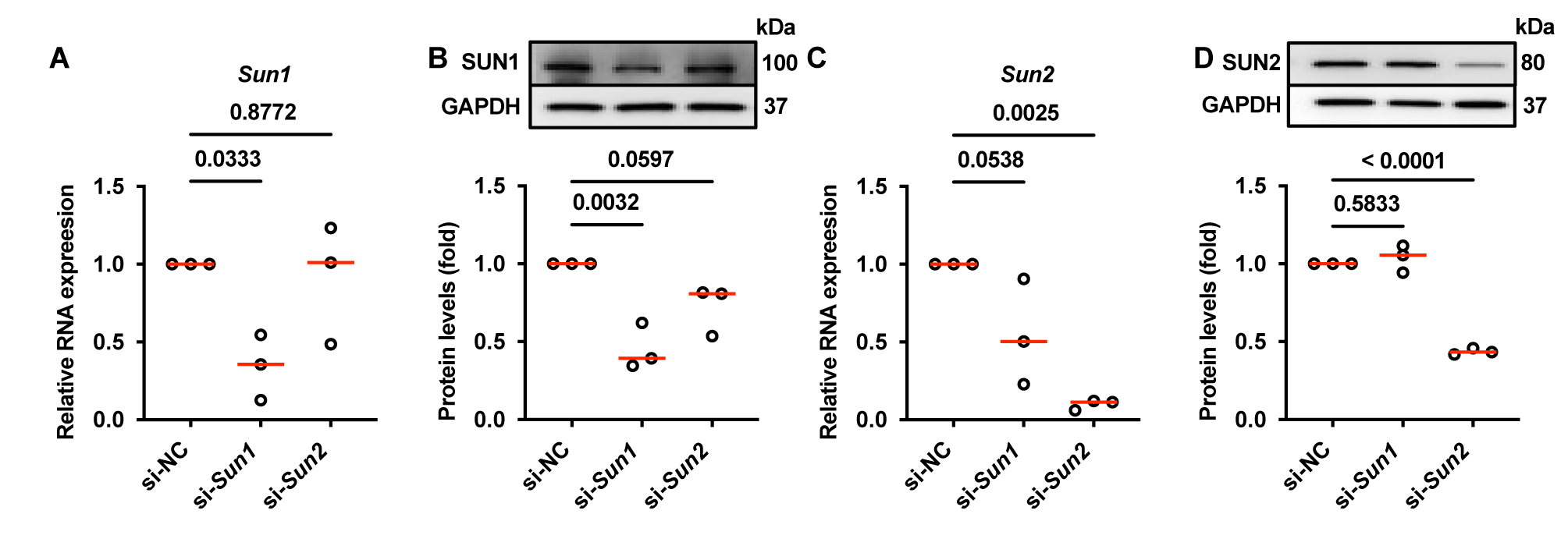

Supplement: Supplementary 1 — Figs. S1 to S21 [file research.1259.f1.zip › FigS4.tif]

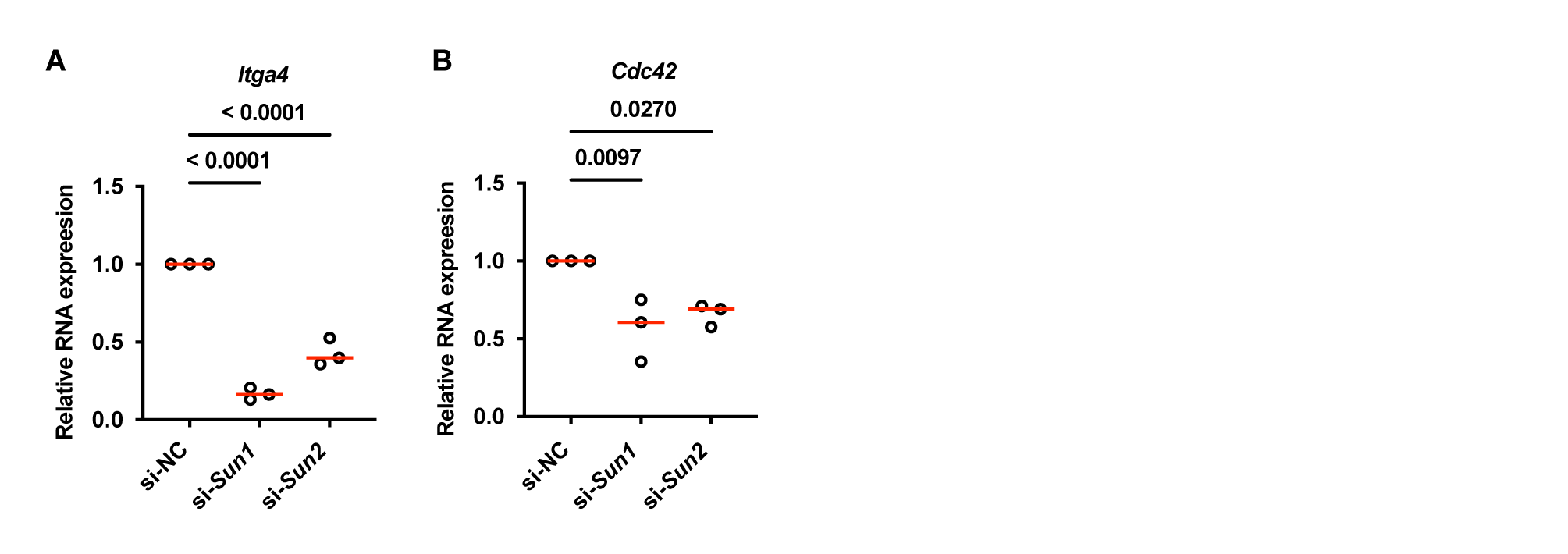

Supplement: Supplementary 1 — Figs. S1 to S21 [file research.1259.f1.zip › FigS5.tif]

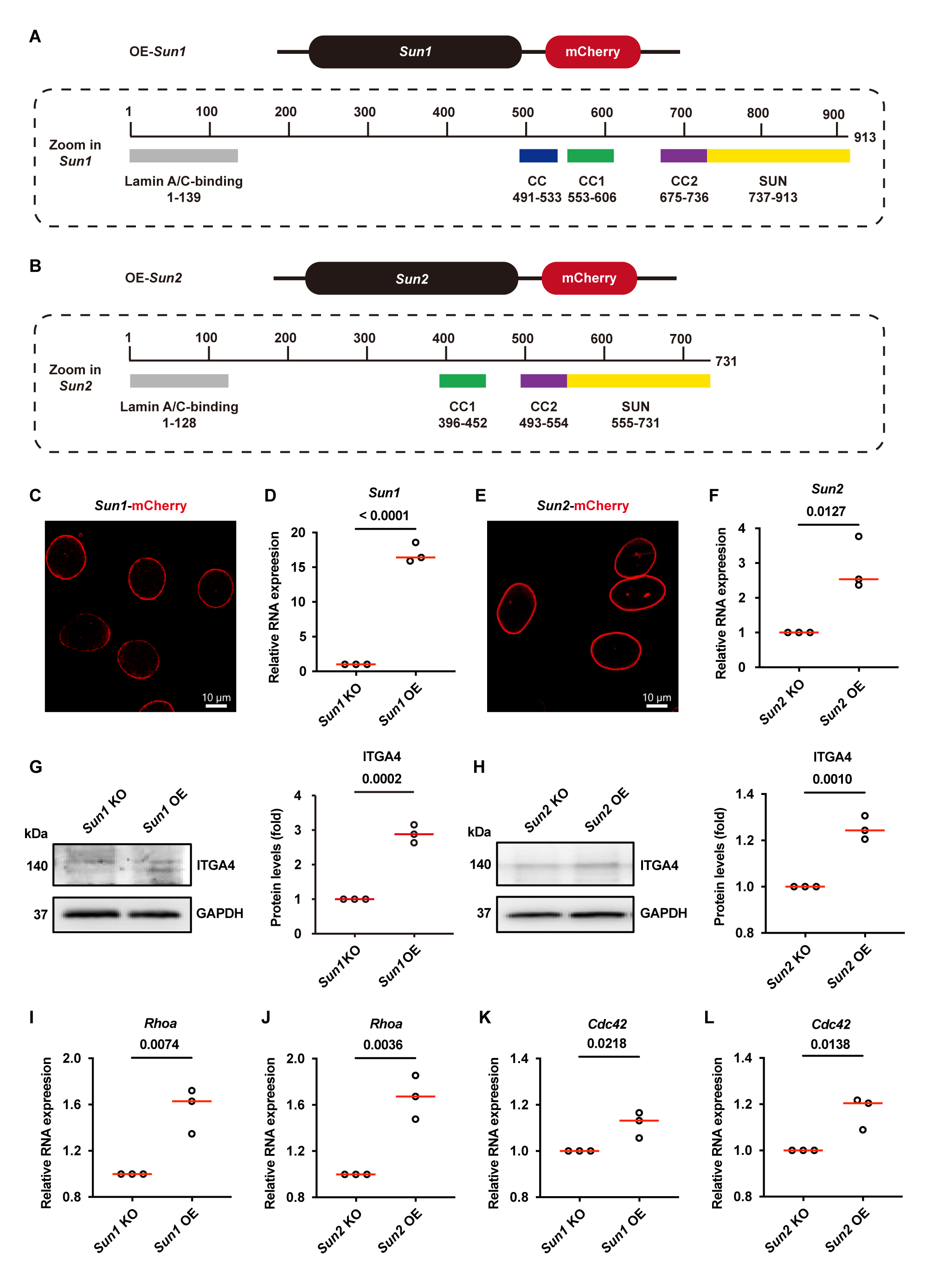

Supplement: Supplementary 1 — Figs. S1 to S21 [file research.1259.f1.zip › FigS6.tif]

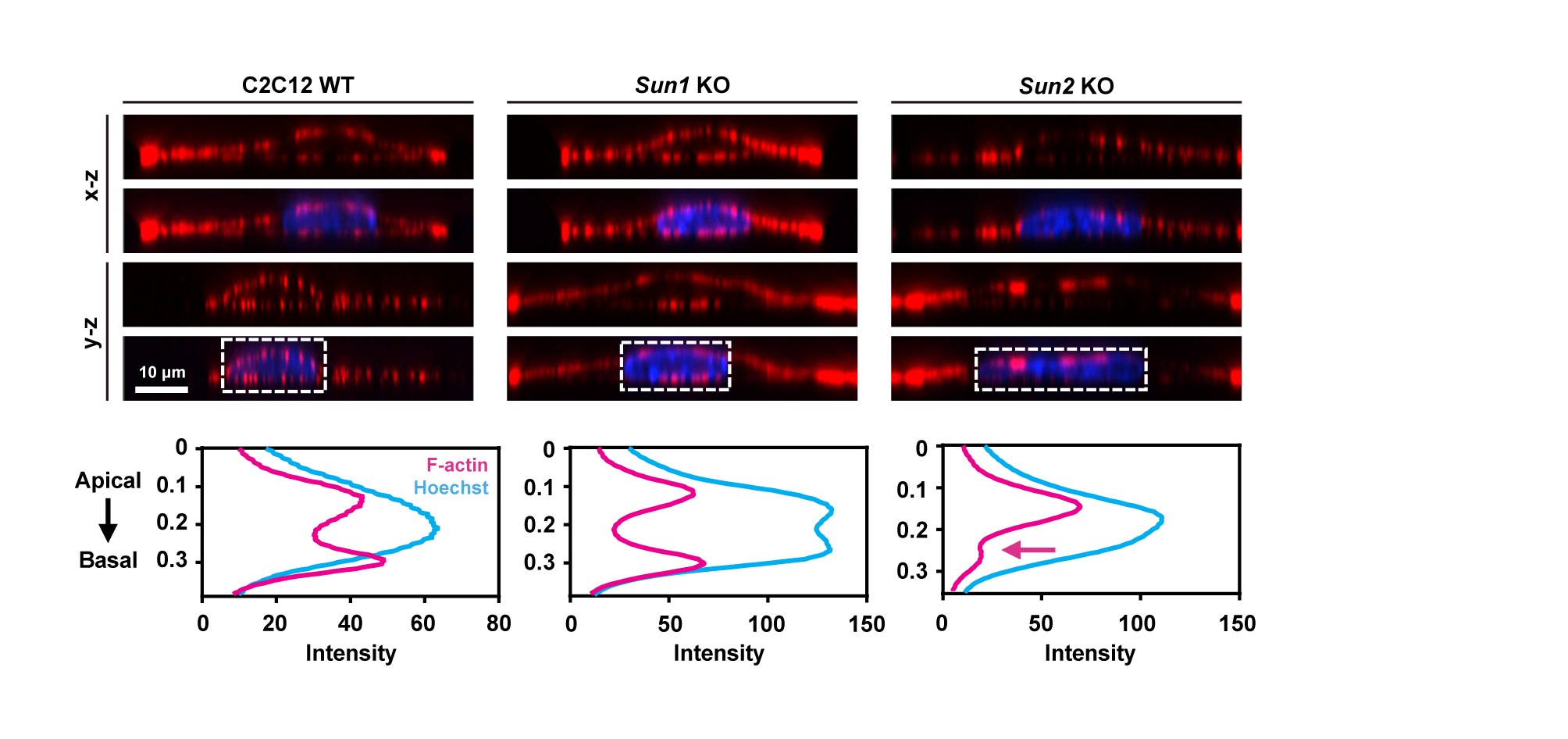

Supplement: Supplementary 1 — Figs. S1 to S21 [file research.1259.f1.zip › FigS7.tif]

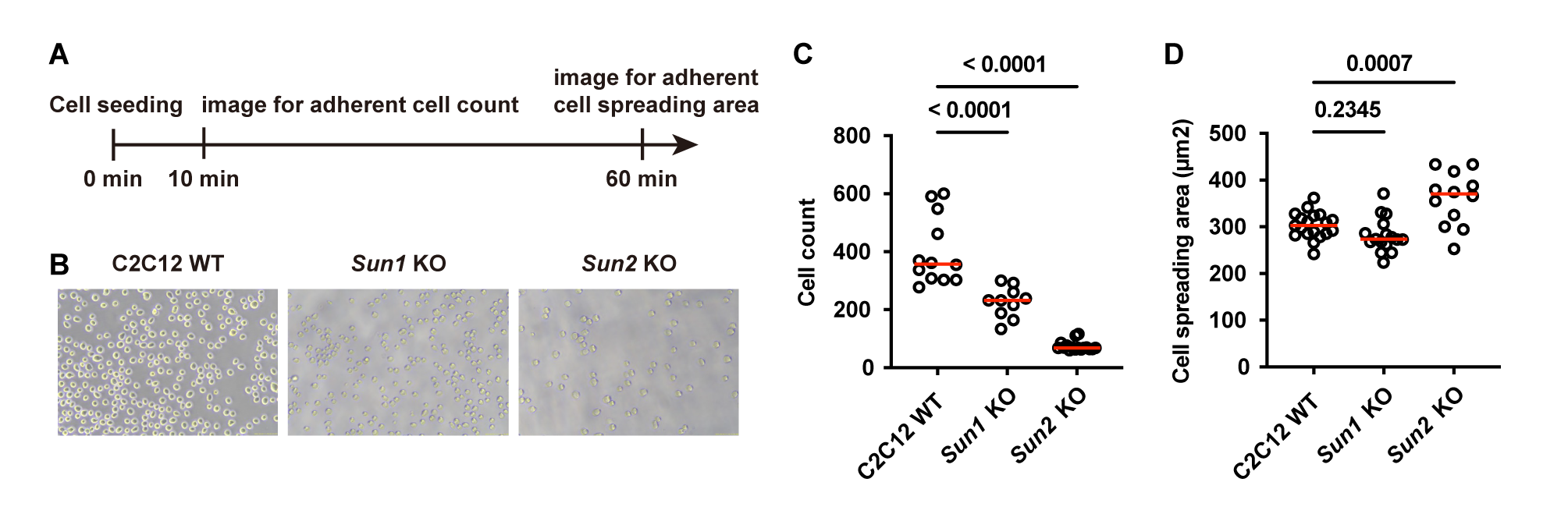

Supplement: Supplementary 1 — Figs. S1 to S21 [file research.1259.f1.zip › FigS8.tif]

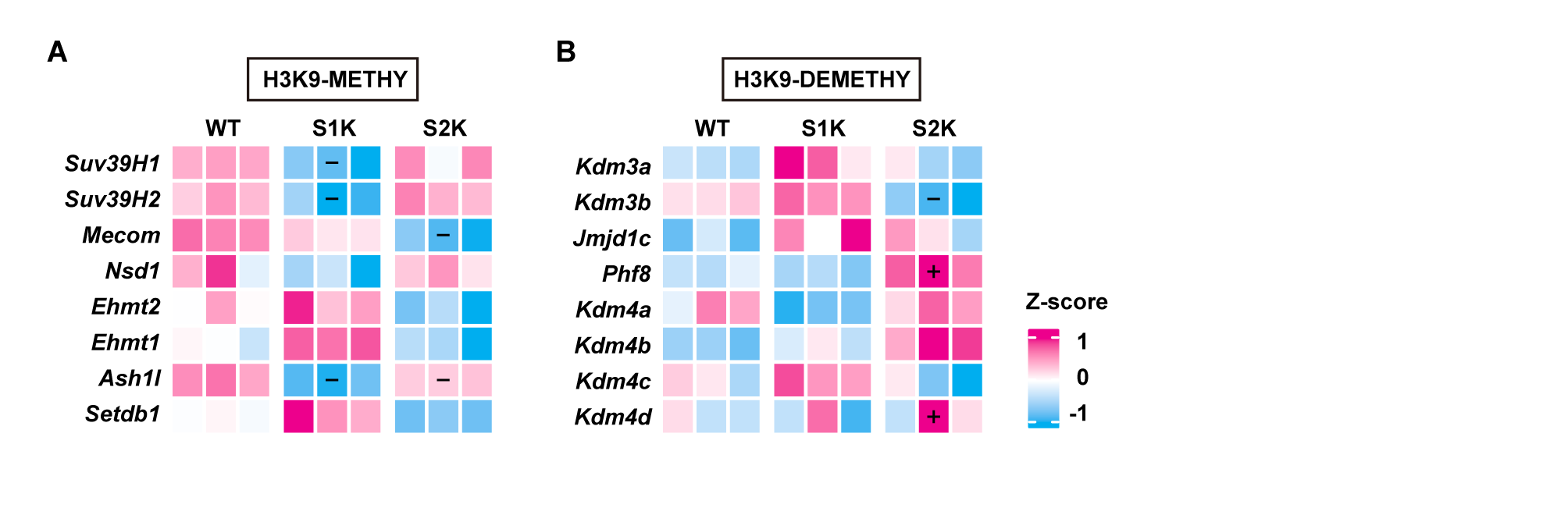

Supplement: Supplementary 1 — Figs. S1 to S21 [file research.1259.f1.zip › FigS9.tif]
